# Illumination of Targeting Nanotherapeutics with Precision Eyes: From Optical to Radio-nanotheranostics

**DOI:** 10.7150/thno.130824

**Published:** 2026-06-25

**Authors:** Maharajan Sivasubramanian, Yao-Chen Chuang, Chia-Hui Chu, Yu Hsia, Li-Jie Lin, Leu-Wei Lo

**Affiliations:** 1 Institute of Biomedical Engineering and Nanomedicine, National Health Research Institutes, Zhunan 35053, Taiwan.; 2 Department of Radiation Oncology, Taipei Medical University Hospital, Taipei 110301, Taiwan.

**Keywords:** nanotheranostics, phototherapies, radiotherapy, radionuclides, programmed cell death, immunomodulation and stimulator of interferon genes

## Abstract

Cancer continues to rank among the deadliest diseases globally, claiming numerous lives due to its high mortality rates. Chemotherapy, a primary form of cancer treatment, offers significant benefits but is hampered by drawbacks that compromise patients' quality of life. Consequently, developing minimally invasive treatment alternatives remains a formidable challenge. Among these, nanomaterial-based, light-activated phototherapies—including photodynamic therapy, photothermal therapy, and radiotherapy—emerge as promising options, providing precise spatial and temporal control with reduced invasiveness. Advances in nanoscience and engineering have led to the creation of nanoparticles (NPs) that integrate therapeutic and diagnostic capabilities, known as theranostics, which enhance the effectiveness of clinical cancer management. This review summarizes recent advancements in nanotheranostics, spanning optical to radio-based approaches. We discuss the roles of various nanomaterials, such as upconversion NPs, gold NPs, nano-scintillators, and mesoporous silica NPs, among others, highlighting their dual diagnostic and therapeutic functionalities. Furthermore, NP-mediated induction of programmed cell death mechanisms—ferroptosis, pyroptosis, and cuproptosis—underscoring their significance in targeted cancer therapies and immune modulation were also explored. Additionally, we discuss how NPs can activate the stimulator of interferon genes pathway, amplify antitumor immunity and offer new avenues for improving cancer treatment outcomes.

## Introduction

Cancer is one of the leading causes of death worldwide, with mortality rates continuing to increase according to the World Health Organization (WHO) estimates 1. However, systemic chemotherapy (CMT), a main clinical treatment modality for cancer, is associated with setbacks, such as poor bioavailability and heterogeneous drug distribution within tumors as well as drug resistance 2, 3. Phototherapies, such as photodynamic therapy (PDT) and photothermal therapy (PTT), have emerged as alternatives primarily owing to the following reasons: 1) reduced toxicity and selective activation for penetration into various tissues and 2) minimal invasiveness with spatiotemporal precision and control of irradiation on the disease site. In PDT, excitation of a photosensitizer (PS) using a suitable wavelength generates reactive oxygen species (ROS) to selectively destroy the cancer cells 4-8. However, classical PDT with ultraviolet (UV) or visible (Vis) light-activated PSs are confined to surface-accessible tumors due to poor tissue penetration. Near-infrared (NIR) light (NIR-I (700–900 nm) and NIR-II (1000–1700 nm)) within the biological transparency window provides deeper tissue penetration because of the minimal absorbance of endogenous chromophores in the NIR range 9, 10. Consequently, there have been significant efforts to develop NIR light-activated PSs to extend the clinical applications of PDT. NIR PDT can be classified into the following three types: 1) direct absorption of NIR light by PS, 2) two-photon (TP) absorption by organic PSs and nanomaterials that have a TP absorption cross-section for TP PDT 11, 12, and 3) upconversion nanoparticles (UCNPs), in which nanomaterials absorb NIR light and emit fluorescence in the UV or Vis wavelength range, thereby enabling the therapeutic potential of classical UV–Vis PS to be realized indirectly. Furthermore, the emission characteristics of UCNPs can be tailor-made according to PS absorption by carefully selecting the type and number of ions doped 13-15. Conversely, PTT is based on the activation of PS by a suitable light to induce local heat to ablate cancer cells. Numerous NIR-induced photothermal conversion nanomaterials have been shown to be excellent PTT agents 16-18. Although the application of standalone NPs for either therapy or imaging has yielded moderate success, recent developments in nanoscience have further prompted researchers to produce multifunctional nanomaterials. This venture resulted in nanotheranostics, an emerging paradigm that implements diagnostic and therapeutic functions in a single nano-entity, and has found potential applications. For instance, NP-mediated PTT/PDT combined with imaging modalities, such as computed tomography (CT), optical imaging, magnetic resonance imaging (MRI), etc., have been reported as useful cancer theranostics 19, 20. Besides the image-guided PTT/PDT, nuclear medicine, which involves the use of radioactive nuclides for therapy and imaging (positron emission tomography (PET) and single-photon emission tomography (SPECT)), has been well received and is rapidly advancing 21-23. Recently, Cherenkov radiation (CR) and Cherenkov luminescence have also been explored as radiotheranostics. CR occurs when charged particles from radioactive decay traverse a dielectric medium with a speed greater than the velocity of light luminesce in the UV–blue spectrum, which in turn can be used to excite PSs to generate ROS 24, 25. Using nanotechnology, radiolabeled NPs can be designed and used for diagnostic and therapeutic purposes when tagged with appropriate radionuclides 26-28.

The transition from optical to radioactive nanotheranostics represents a paradigm shift from qualitative preclinical testing to quantitative clinical application. While optical modalities such as NIR-I and NIR-II fluorescence offer high spatial resolution and real-time intraoperative guidance, they remained inherently limited by tissue penetration depth, where signal attenuation and autofluorescence impede deep-seated tumor detection. The evolution toward radioactive modalities was primarily fueled by three driving factors: First, the high energy of gamma rays and positrons ensures minimal attenuation, allowing for whole-body imaging regardless of tissue depth. Second, unlike optical signals, which are relative, radiotracers allow for absolute quantification of injected dose per gram of tissue. Third, the chemical interchangeability of diagnostic isotopes with therapeutic emitters provided a seamless pathway for 'see-and-treat' protocols.

Optical nanotheranostics rely on externally applied light (e.g., visible/NIR) to activate NP for imaging and therapy, with design focusing on optical absorption, wavelength tuning, and energy conversion efficiency. In contrast, radio-based nanotheranostics include radiotherapy (RT)/radiotheranostic strategies that utilize radionuclides as internal energy sources for tumor imaging and therapeutic radiation delivery, with design considerations centered on radionuclide stability, radiolabeling efficiency, dosimetry control, and *in vivo* pharmacokinetics. We further explain that these two modalities are complementary rather than isolated, as optical systems offer precise spatiotemporal control but are limited by tissue penetration, whereas radiological approaches overcome depth constraints and can also be integrated with optical mechanisms in hybrid platforms such as scintillator-mediated systems.

Herein, we discuss the latest developments in nanotheranostics ranging from “optical to radio” using various NP strategies by describing their design of systems, operational mechanisms, and applications. NP-induced immunogenic cell death (ICD) has emerged as a promising therapeutic approach 29, 30. Cancer cells often develop resistance mechanisms to evade programmed cell death (PCD), thereby reducing the effectiveness of conventional treatments. In this context, nanotechnology provides innovative solutions by providing targeted delivery systems and enabling precise modulation of PCD pathways within the tumor microenvironment (TME). This review also focuses on the role of NPs in promoting PCD in cancer cells such as ferroptosis, cuproptosis, and pyroptosis to improve therapeutic outcomes while simultaneously sparing normal tissues 31, 32. We analyze the design, functionalization, and interactions of NPs within cancer cells to induce PCD and activate innate immune pathways 33, 34. Moreover, this review discusses the therapeutic potential of harnessing these pathways for improved cancer treatment.

## Functional PDT

### NIR PDT

Classical PDT in which a PS that is activated by UV–Vis light is used suffers from poor efficacy due to limited penetration depth and inappropriate activation of the PS 35. A viable alternative to avoid these problems is the use of PSs that are responsive to NIR light (NIR–I (700–900 nm) and NIR–II (1000–1700 nm)). In these biological transparent windows, tissues have relatively low attenuation coefficients compared with that in UV–Vis windows and thus improved penetration depth 36-38. UCNPs comprise an important method to deliver light into deep tissues for PDT. Lanthanide-doped UCNPs, as an energy transducer, emit high-energy photons when excited with lower-energy NIR photons 14, 39. The merits of UCNPs include the following: 1) they can realize the therapeutic potential of well-investigated and confirmed traditional UV light–activated PSs and 2) they are endowed with exciting optical properties, such as improved penetration depth, well–stabilized photochemical properties, and no autofluorescence background 40. Herein, we discuss the advancements of UCNP-mediated deep PDT in the subsequent section.

A hydrogen peroxide-driven black TiO₂ mesoporous Janus nanomotor (HTiPC) was designed to enhance tumor penetration and NIR-triggered PDT 41. Comprising a NIR-responsive H-TiO₂₋ₓ nanosphere and a catalase (CAT)-modified periodic mesoporous organosilica (PMO) nanorod, the nanomotor autonomously navigates through the TME by catalytically decomposing overexpressed H₂O₂ into oxygen and water. This process not only propels the nanomotor but also alleviates tumor hypoxia, improving ROS generation for PDT. The optimized HTiPC-ii nanomotor demonstrated enhanced self-propulsion, increased cellular uptake, and deep tumor penetration, leading to effective intracellular oxidation and apoptosis under 808 nm NIR irradiation. *In vivo* studies confirmed superior tumor inhibition, highlighting the potential of these TiO₂-based mesoporous nanomotors as a promising strategy for deep-tissue cancer treatment.

NIR PS Secy7, a selenium-substituted heptamethine cyanine, was developed to overcome the low singlet oxygen (^1^O_2_) generation in PDT 42. By inducing an intramolecular charge transfer effect and leveraging the heavy-atom effect of Se, Secy7 significantly narrows the energy gap (0.51 eV), enhances spin–orbit coupling (1.44 cm⁻¹), and achieves a high triplet state yield (61%), leading to an ultrahigh ^1^O_2_ quantum yield. Under low-power 850 nm irradiation, Secy7 demonstrated a ~24.5-fold higher ^1^O_2_ generation than indocyanine green (ICG) and exhibited excellent phototoxicity with minimal dark toxicity. *In vivo* studies confirmed its potent antitumor and antimetastatic effect. A NIR photoactivatable nano-PROTAC (NAP) was developed for precise, spatiotemporal control of protein degradation in tumors. NAP consists of a PROTAC linked to an NIR PS via a ^1^O_2_-cleavable linker, forming a self-assembled nanoformulation with initially silent proteolytic activity. Upon systemic administration, NAP accumulates in tumors through the enhanced permeability and retention (EPR) effect. NIR irradiation at the tumor site generates ^1^O_2_, cleaving the linker to activate PROTAC, which then degrades BRD4 and sensitizes cancer cells to PDT 43.

KD1@HPEG NP, a NIR-II PDT platform was designed for repeatable, precise, and durable cancer treatment. This nanoplatform combines deep-tissue NIR-II fluorescence imaging (FLI) with a Type I PS (KD1) that can generate oxygen-independent ROS. KD1 was engineered to improve intersystem crossing, reduce energy loss, and minimize unwanted photothermal heating. The hyaluronic acid-PEG coating improves stability, provides intrinsic tumor targeting, and importantly avoid anti-PEG immune responses, allowing repeated dosing without rapid clearance. Mechanistically, the nanoplatform not only damages tumors through ROS but also triggers mitochondrial dysfunction and ferroptosis-related pathways, for robust tumor killing 44.

NanoPcCu, a NIR-activated nano-photocatalyst, was designed to improve PDT in hypoxic tumors. NanoPcCu can generate Type I reactive species such as O₂•⁻ and •OH even under low-oxygen conditions, making it much more effective in the hypoxic TME. Under 685 nm light, the NPs also generate heat for PTT, so the treatment combines PDT and PTT for robust tumor killing. In addition, NanoPcCu can perform catalytic reactions, which generate more toxic radicals through Cu(II)/Cu(I) photoredox cycling, further amplifying oxidative damage. The system also enables photoacoustic imaging (PAI)-guided therapy, allowing tumor monitoring during treatment. Importantly, NanoPcCu also promoted dendritic cell maturation and systemic antitumor immune responses, that controls the growth of both primary and distant tumors 45.

A NIR-responsive “dark state photocage” nanoplatform that combines PDT and CMT was designed and developed. The system stays largely inactive before irradiation, but under 650–660 nm light, it undergoes efficient photolysis even at low power, allowing deep-tissue activation with reduced off-target effects. When irradiated, methylene blue (MB) phenothiazine/phenoxazine-based cages transition from ground state to excited state, which promotes intersystem crossing and triplet-state formation, allowing cargo release. In the representative system MB-Lv, NIR light triggers the release of both STAT3 inhibitor (Lv) and MB, which produces ^1^O_2_ for PDT. As a result, enhanced PDT + CMT synergistic effect was observed, along with a clear fluorescence turn-on signal for imaging-guided treatment 46. An 808 nm NIR light-activatable prodrug system for photoimmunotherapy of unresponsive tumors was developed. BODIPY-based PS can be activated by 808 nm light, which penetrates tissue more deeply and enables spatiotemporal drug release at the tumor site. In this system, R837 was linked to the NIR PS through a ROS-cleavable linker. Upon laser irradiation, the NPs generated ROS and heat, which not only caused direct phototherapeutic tumor killing but also cleaves the linker to release R837. This dual action promoted ICD, release of tumor antigens and DAMPs, dendritic cell maturation, and stronger CD8⁺ T-cell infiltration 47

A mitochondria-targeting nanoplatform (NZ@TG) was developed to overcome the shallow penetration and hypoxia limitations of conventional PDT by using 808 nm NIR light. The system combines a lanthanide-doped nanocrystal with a phthalocyanine PS and a hypoxia-activated prodrug, enabling dual action: localized oxidative damage in mitochondria and hypoxia-triggered DNA damage. This synergistic effect induces strong ICD, releasing DAMPs that activate systemic anti-tumor immunity, including macrophage polarization toward the M1 phenotype and activation of CD8⁺ T cells. Importantly, NIR-PDT achieved significant tumor suppression in both subcutaneous and orthotopic liver tumor models, demonstrating effective treatment of deep-seated tumors. It also generated long-term immune memory, reduced metastasis and recurrence, and significantly enhanced the efficacy of immune checkpoint blockade (ICB) therapy 48.

Studies have demonstrated that mild hyperthermia improves tumor oxygenation in rodent, canine, and human tumors. The increase in the tumor oxygenation lasted for up to 1–2 days after heating at mild temperatures (about 39–42 °C). The increase in oxygenation occurs mainly due to enhanced tumor blood perfusion 49. In one study, semi-quantitative analysis of hypoxia-positive signals in tumor slices after mild NIR PDT (~45 °C) treatment with DiR-hCe6-liposome showed a significant reduction in tumor hypoxia from about 38% to ~12% 50. PTT can trigger the upregulation of heat shock proteins (HSPs), which protect cancer cells and reduce their sensitivity to heat-induced apoptosis 51. This limitation can be overcome by combining PTT with PDT. In this strategy, the ROS generated during PDT can damage HSPs and directly kill cancer cells, thereby enhancing the overall therapeutic effect 52. Younis *et al.* demonstrated that a nanoplatform composed of AuNRs/MoS₂ loaded with ICG enables simultaneous PDT and PTT under a single low-power NIR laser (0.2 W/cm²). The system showed high photothermal conversion efficiency (PCE) (68.8%), raising the temperature to ~60 °C within 5 min. The heat generated by the dual plasmonic NP triggered the release of ~85% of ICG, leading to ^1^O_2_ production for PDT. As a result, the combined PDT/PTT eradicated cancer cells and tumors *in vitro* and *in vivo*, showing significantly higher antitumor efficacy than either PDT or PTT alone 53.

### UCNPs PDT

UCNPs absorb low-energy NIR light and convert it into higher-energy UV or visible light 54-56. Because NIR light can penetrate deeper into tissues with less absorption by biological components, UCNPs enable activation of light-responsive systems in deeper regions 57. Their lanthanide-doped structure allows efficient energy conversion, making them useful as energy transducers to generate ROS and enhance processes like PDT 58, 59**.**

**Figure 1:**
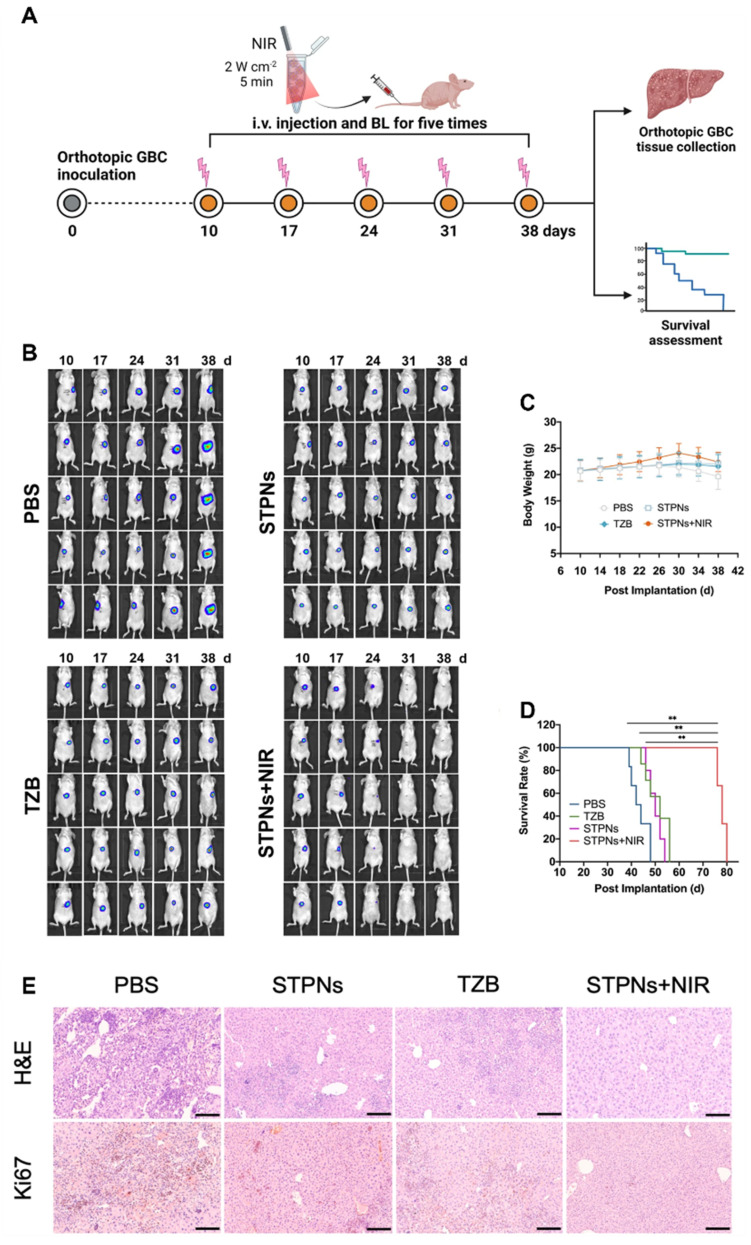
**(A)** Schematic illustration of orthotopic GBC inoculation and treatment, **(B)** *in vivo* bioluminescence images of orthotopic GBC-bearing mice treated with various formulations irradiated by a 980 nm laser (2.0 W cm⁻², 5 min), **(C)** body weight changes, **(D)** Kaplan–Meier survival curves, and **(E)** H&E and Ki67 staining (scale bar = 250 μm). Adapted with permission from 61, Copyright 2023 Springer Nature.

The following study presents an advanced PDT system using core-multishell UCNPs capable of emitting three distinct colors—red, green, and blue—when exposed to different NIR wavelengths (1550 nm, 808 nm and 980 nm) 60. These UCNPs are integrated with PSs and nitric oxide (NO) donors to create a programmable "off–on" nanoplatform for precise and controlled phototherapy. By tuning the luminescent and inert shell thicknesses, the UCNPs independently activate imaging, NO release, and ROS generation, thus allowing for stepwise and targeted treatment. Remarkably, the sequential release of NO before PDT reduces tumor hypoxia, thereby improving the overall therapeutic efficacy (**Figure 1**). A study introduces stimuli-sensitive tumor-targeted photodynamic NPs (STPNs) designed for deep tumor treatment, overcoming challenges such as limited NIR tissue penetration and poor accumulation at target sites 61. These STPNs combine lanthanide-doped UCNPs for improved imaging and photostability with the PS Purpurin 18 (Pu18), which exhibits persistent luminescence (PL). Before intravenous administration, STPNs are preactivated by 980 nm NIR laser irradiation, allowing them to accumulate at tumor sites and enter cells via HER2 receptors. Within the TME, STPNs disassemble, thereby enabling the UCNPs to trigger the photoactivity of Pu18 and produce ROS for effective antitumor therapy. Furthermore, STPNs provide diagnostic capabilities through MRI and intraoperative NIR imaging due to their gadolinium content.

A cold-responsive nanoplatform (UCNPs@SiO_2_-Ce6-HA) that integrates cryotherapy with PDT for enhanced melanoma treatment was developed. The low-temperature environment amplifies upconversion luminescence (2.45-fold) and ^1^O_2_ production (3.15-fold), significantly boosting PDT efficacy. The HA coating ensures efficient transdermal delivery, leading to a remarkable 79% tumor growth inhibition in melanoma-bearing mice, outperforming cryotherapy (17%) and PDT (55%) alone 62. A recent study introduced a programmable NIR–controlled nanosystem (PB@UA) to enhance PDT by overcoming tumor antioxidant defenses 63. Using switchable UCNPs, the system independently triggers berbamine (BBM) release at 980 nm and activates a PS (ZnPc) at 808 nm. BBM inhibits antioxidant enzyme activity and disrupts calcium ion regulation, making tumor cells more vulnerable to ROS induced apoptosis. This sequential activation significantly improves PDT efficiency, achieving an 80.91% tumor inhibition rate, surpassing PDT (31.78%) or BBM (11.29%) alone. UCNPs@AgBiS_2_ core-shell NPs were successfully synthesized using an ion exchange reaction, enhancing PCE from 14.7% to 45% through cross-relaxation between Nd ions and AgBiS_2_ 64. The optimal Nd ion doping (1%) in the inner core facilitated strong upconversion emissions, which excited the AgBiS2 shell to generate ROS for PDT. These NPs demonstrated significant antitumor efficacy *in vitro* and *in vivo* under 808 nm laser irradiation by combining PTT and PDT.

Tumor-targeted upconversion nanospheres (ALUMSNs) that combine upconversion PDT and PTT with multimodal imaging was developed by Palanikumar *et al.* NaYF₄:Yb/Er core converts deeply penetrating NIR light into visible light, which then activates Ce6 to generate ROS for PDT, while Bi₂Se₃ simultaneously generates heat for PTT. The nanosystem was further designed with a mesoporous silica shell for stable Ce6 loading, a lipid/PEG coating, and an ATRAM peptide that helps selective uptake in the mildly acidic tumor environment. In addition, Gd in the core enables MRI, and the system also supports thermal and fluorescence imaging, allowing treatment monitoring 65.

Chu *et al.* developed UCNPs@AgBiS₂ core–shell NPs as an upconversion-based PDT/PTT platform for cancer treatment. The Nd³⁺-sensitized upconversion core converts 808 nm NIR light into higher-energy emission, which then activates the AgBiS₂ shell to generate ROS for PDT. At the same time, energy interaction between Nd ions and AgBiS₂ greatly improved the PCE from 14.7% to 45%, making the system much stronger for PTT as well. 1% Nd in the inner core produces strong upconversion emission and better therapeutic performance 66. A smart upconversion-assisted radiosensitizer (RS) platform (DH&UH NPs) that improves both tumor imaging and RT was developed by Mo *et al.* The upconversion NPs (UH NPs) produce UV light under 980 nm NIR irradiation, which remotely triggers *in situ* NP aggregation through chemical bond formation with the downconversion partner (DH NPs). This aggregation greatly prolongs tumor retention, allowing long-term NIR-II FLI for treatment monitoring and tumor tracking. At the same time, the system carries diselenide-based radiosensitizing groups, which generate strong oxidative products and increase ROS under X-ray exposure, making cancer cells more sensitive to RT 67.

Wang *et al.* developed PURH, a smart upconversion-based PDT immunotherapy nanoplatform that can be controlled by two different NIR light for precise cancer treatment. Under 980 nm irradiation, the system activates rose bengal (RB) to generate ROS, causing photodynamic tumor killing and ICD. Under 808 nm irradiation, the same platform triggers the controlled release of CpG, which promotes dendritic cell maturation and T-cell infiltration. The mesoporous silica shell stores RB, while hyaluronic acid helps tumor targeting 68. A carbon-based upconversion nanocomposite was developed by Yang *et al.* to improve light utilization and therapeutic performance under 980 nm irradiation. By coating mesoporous carbon NPs with a lanthanide oxysulfide layer (Y₂O₂S:Yb³⁺,Er³⁺), the system converts NIR light into visible emission, which enhances photothermal efficiency (from ~59% to ~83%) and supports upconversion-assisted photodynamic effects. This platform enables rapid heating (~50 °C within 150 s) and efficient tumor ablation while also carrying drugs for combined therapy with controlled release via a stimuli-responsive hydrogel. *In vitro* and *in vivo* studies, including subcutaneous and ocular melanoma models, showed strong tumor targeting, significant reduction in cancer cell activity, and marked inhibition of tumor growth. The treatment activates tumor-suppressive pathways and suppresses proliferation-related signaling, improving drug sensitivity 69.

### TP PDT

TP PDT provides deep–tissue penetration by absorbing two low-energy NIR photons simultaneously and emitting high-energy photons. TP PDT involves nonlinear photon absorption, which allows three-dimensional selectivity to target tumor cells, a critical feature that cannot be achieved by conventional single-photon absorption 11, 70. Herein, we discuss various NP-based TP PS approaches for deep PDT. Using the well-ordered mesoporous structure of MSN, Cheng *et al.* chemically attached a donor TP antenna molecule in the silica framework and an acceptor PS molecule in the nanochannels. This precise design allowed an efficient FRET-mediated energy transfer rate of up to 93% to produce cytotoxic ROS that exerted improved PDT effects in a breast cancer model 71.

**Figure 2:**
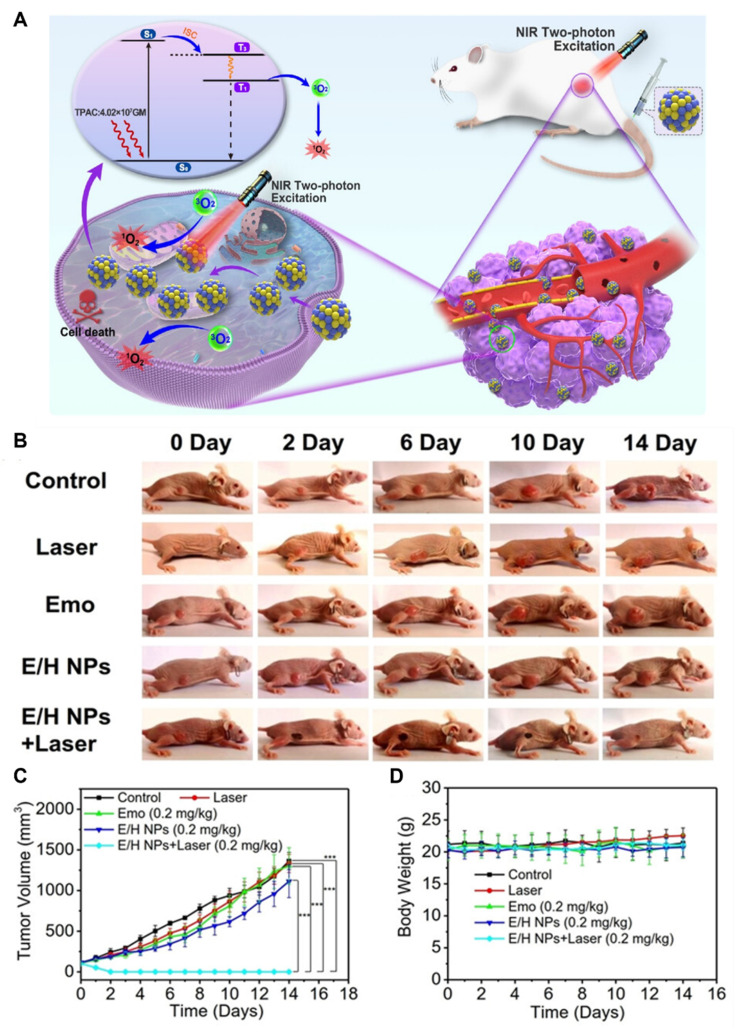
**(A)** Schematic illustration of E/H NPs for efficient anti-cancer TPE-PDT, **(B)** photographs of tumor-bearing mice at various time points after treatment, **(C)** tumor volume changes, and **(D)** body weight changes. Adapted with permission from 73, Copyright 2023 John Wiley and Sons.

This study shows the development of a novel PDT PS, BF_2_DCz, an organic material with room-temperature phosphorescence (RTP) characteristics 72. Encapsulated within a bovine serum albumin matrix, BF_2_DCz-BSA demonstrates high photoluminescence quantum yield (47.7 ± 3%), impressive intersystem crossing efficiency (~90.3%), excellent biocompatibility, negligible dark toxicity, and superior photostability (**Figure 2**). Using an NIR femtosecond laser, the material achieves efficient ROS generation, enabling precise spatial and temporal therapeutic control. In addition to demonstrating selective blood vessel closure and TP imaging in the mouse brain vasculature, this study emphasizes the potential of BF2DCz-BSA for deep-tissue PDT, providing a promising approach for improving the outcomes of cancer therapy. Emo/HSA NPs (E/H NPs), a highly efficient TP-PDT agent derived from emodin, a natural anthraquinone, was developed and studied 73. The NPs exhibited an exceptional TP absorption cross-section (4.02 × 10⁷ GM) and significant ^1^O_2_ quantum yield (31.9%). *In vitro*, the E/H NPs exerted improved anticancer effects against MCF-7 cells, whereas *in vivo* experiments revealed prolonged tumor retention and effective tumor ablation at an ultra-low dosage (0.2 mg/kg) under 800 nm femtosecond laser irradiation.

A novel nanohybrid combining thiolated chitosan-coated gold (Au) nanostars (AuNS-TCS) and riboflavin-conjugated N,S-doped graphene quantum dots (Rf-N,S-GQDs) for synergistic TP-PDT/PTT was synthesized 74. Using a single low-power pulsed laser (760 nm, 200 mW·cm⁻²), the nanohybrid leverages the spectral overlap between the localized surface plasmon resonance (LSPR) of AuNS and the TP absorption of Rf-N,S-GQDs, achieving simultaneous photodynamic and photothermal effects. The thiolated chitosan coating improves colloidal stability and facilitates the anchoring of Rf-N,S-GQDs, and the plasmonic effect significantly boosts ^1^O_2_ generation and photothermal performance. Compared with individual therapies, the combined TP-PDT/PTT system exhibits superior phototoxicity and therapeutic outcomes against 2D cell monolayers and 3D tumor spheroids, highlighting its potential for efficient and simplified cancer treatment under single-laser activation. The first development of metal-free NIR thermally activated delayed fluorescence NPs (NIR-TADF NPs) as effective agents for TP PDT and imaging was presented in this study 75. These NPs exhibit excellent ^1^O_2_ generation, inherent TP excitation, and NIR fluorescence emission, demonstrating remarkable biocompatibility and biosafety. Validated in A549 tumor xenograft models, the NIR-TADF NPs exerted significant antitumor effects and high precision in both single-photon and TP imaging and therapy.

A hypoxia-activated, novel mitochondria-localized iridium (III) endoperoxide prodrug (2-O-IrAn) was designed for synergistic PDT and photoactivated CMT (PACT) was developed 76. Activated by NIR TP irradiation, 2-O-IrAn releases a cytotoxic iridium (III) complex, ^1^O_2_, and alkoxy radicals, demonstrating high phototoxicity in hypoxic tumor cells and multicellular tumor spheroids at low concentrations. Encapsulation in biotin-functionalized phospholipid NPs enhanced tumor selectivity and pharmacological properties, enabling near-complete tumor eradication in a mouse model after a single treatment. The limitations of Ru(II) polypyridine complexes in PDT were addressed by coordinating them with graphitic carbon nitride (g-C₃N₄) nanosheets to create an oxygen-self-sufficient TP PDT immunotherapy 77. The conjugates exhibit increased TPA, which is significantly stronger than that of molecular Ru(II) complexes, and generate a robust ROS storm, even under hypoxic conditions, by catalytically converting H₂O/H₂O₂ into O₂. Encapsulation with an amphiphilic polymer improved the pharmacological properties, thereby enabling mitochondrial and endoplasmic reticulum (ER) accumulation. Upon irradiation with a deeply penetrating 800 nm laser, the nanomaterial induced cell death via apoptosis, paraptosis, ferroptosis, and ICD in both monolayer and multicellular tumor spheroids. In a melanoma-bearing mouse model, it significantly inhibited tumor growth in primary tumors and activated immune responses to secondary distant tumors, exhibiting its potential to treat hypoxic solid tumors and metastatic cancer.

MeTTh, a high-performance TP PS for treating small residual glioblastoma (GBM) was developed by Li *et al.* MeTTh was designed to have a large TP absorption cross-section, strong ROS generation, and both Type I and Type II photodynamic activity, making it useful even in the hypoxic GBM environment. After transferrin conjugation, the modified NPs successfully targeted orthotopic GBM. It also enabled deep TP imaging of brain structures up to 940 µm, allowing image-guided therapy 78. A TP theranostic nanoplatform based on mesoporous silica NPs (MTP–MSNs) for simultaneous deep-tissue imaging and targeted cancer therapy was developed by Wu *et al.* The NPs were engineered to produce multicolor TP fluorescence and were capped with the cancer-targeting aptamer AS1411, which recognizes nucleolin overexpressed on tumor cells. Once internalized by cancer cells, the aptamer gate opens the nanopores and releases the loaded drug, enabling selective intracellular therapy 79. Ke *et al.* developed a biodegradable iridium (III)-based coordination polymer NPs (IrS NPs) to improve PDT against cancer. These NPs remain stable in physiological conditions but break apart in the tumor environment, where they reduce glutathione (GSH) levels and release active iridium complexes. The NPs selectively accumulate in mitochondria, making the treatment more precise at the subcellular level. Upon light activation, they generate both ^1^O_2_ and superoxide radicals, triggering cancer cell death through a combination of apoptosis and ferroptosis rather than a single pathway 80.

A sulfur-containing polymer PS for TP PDT was prepared by a simple catalyst-free thiol–yne click reaction. Nanoplatform in this study were designed with a donor–π–acceptor structure, which gave them strong TP absorption in the 700–800 nm range, making them suitable for deeper tissue light activation. The introduction of sulfur enhanced intersystem crossing and improved ^1^O_2_ generation, while polymerization further amplified this effect. In addition, the aggregation-induced emission unit helped reduce energy loss and improve performance in the aggregated NP state. As a result, the optimized NPs showed strong ROS production and effective cancer cell killing under TP excitation 81.

### X-ray PDT

Although fascinating, NIR-based deep PDT suffers from: 1) limited penetration depth (up to 1.5 cm) and 2) difficulty in synthesizing of NIR PS with a broad energy gap. X-rays, as an ionizing radiation with unlimited penetration depth and photon energy ranging from keV to MeV, have been a mainstay in clinical imaging and treatment of cancer, and could be used as an excitation source for deep PDT 82-84. Nevertheless, direct activation of PSs by X-ray cannot be achieved, which has resulted in the development of scintillating NP (SCNPs) 85 and persistent luminescent NP (PLNPs) 86, 87. X-ray excited PDT uses SCNPs to down-convert high-energy X-rays into UV-Vis light to activate PSs, whereas X-ray excited PLNPs can activate PSs even after the cessation of irradiation. Incorporation of heteroatoms (oxygen, nitrogen, and sulfur) significantly enhanced X-ray energy harvesting, intersystem crossing, and subsequent formation of ROS, particularly oxygen-independent •OH and ¹O₂. Encapsulating TBDCR in a crystalline PEG shell increased particle compactness and further boosted ROS production. Remarkably, TBDCR NPs demonstrated strong NIR fluorescence and potent anticancer efficacy against both standard HeLa and radio-resistant HeLaR tumors *in vitro* and *in vivo*, establishing a new direction for purely organic type I and II X-ray-excited PDT (X-PDT) nanoscintillators. NaYF₄:Tb@NaYF₄ core–shell NPs exhibiting enhanced PL were successfully synthesized for improved X-PDT 88. The optimized synthesis significantly enhanced X-ray excitation optical luminescence and persistent luminescence by over 5.2 and 3.5 times, respectively. Covalent conjugation of these NPs with the PS RB created an effective nanocoupling system. A novel hyperfractionated irradiation strategy further amplified ROS production, achieving 85% tumor inhibition in B16–F10 tumor-bearing mice at a low irradiation dose (2 Gy) without notable side effects.

**Figure 3:**
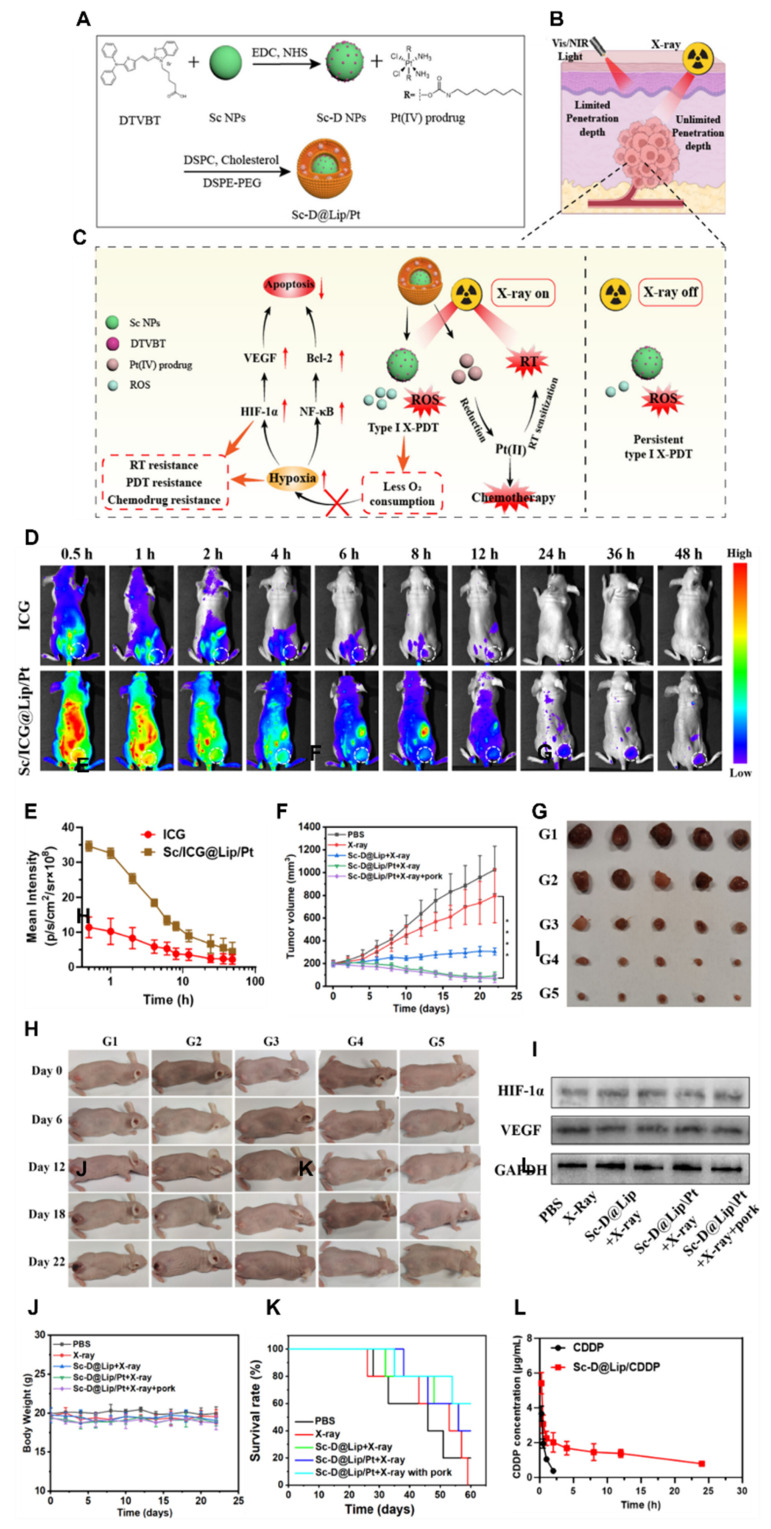
Schematic illustration of the **(A)** design and **(B, C)** mechanism of X-ray activated persistent type I PDT nanoplatform, **(D)** *In vivo* biodistribution of free ICG and ICG-loaded NPs at various time points (white circles indicate tumors), **(E)** Quantitative analysis of fluorescence intensity in tumor regions at different times, **(F)** Tumor growth curves for each treatment group, **(G)** Photographs of tumors from different treatment groups, **(H)** Representative images of mice bearing 4T1 tumors after treatments at different time points, **(I)** Western blot analysis of HIF-1α and VEGF expression in 4T1 tumors after treatments, **(J)** Body weight changes of tumor-bearing mice in each group, **(K)** Survival curves of treated mice, **(L)** Blood circulation profiles of CDDP and Sc-D@Lip/CDDP measured by HPLC following intravenous injection. Adapted with permission from 90, Copyright 2025 American Chemical Society.

Although studies using SCNPs for X-ray PDT produced encouraging results, substantial room remains for research in the following areas. First, improving SCNP parameters, such as size and luminescence properties, could promote efficient energy transfer between SCNPs and PS to enhance ROS production. Second, targeting blood vasculature rather than cancer cells is an attractive strategy to enhance tumor accumulation. Third, validation of *in vivo* properties of NPs using orthotopic tumor models is promising to minimize systemic toxicity. A novel purely organic phosphorescent nanoscintillator was developed to achieve effective X-PDT without relying on heavy-metal-containing inorganic agents 89. These organic nanoscintillators efficiently transferred energy to produce substantial ^1^O_2_ under low-dose X-ray irradiation (0.4 Gy), effectively combining the advantages of RT and PDT (**Figure 3**). *In vivo* experiments demonstrated robust therapeutic performance against deep-tissue tumors with minimal side effects, highlighting the potential for safe, low-dose cancer treatment. A novel nanoplatform (Sc-D@Lip/Pt) was developed to overcome limitations of PDT such as tumor hypoxia, insufficient tissue penetration, and transient ROS generation. Incorporating a type I PS with core–shell scintillator NPs enabled sustained production of less oxygen-dependent ROS (•OH and O₂•–) under X-ray irradiation, continuing for over 4 h even after irradiation ceased 90. Additionally, a Pt(IV) prodrug was integrated and selectively converted into cisplatin within tumors, enhancing chemotherapeutic and radiosensitizing effects. *In vitro* and *in vivo* studies confirmed the platform’s efficacy without aggravating hypoxia or increasing hypoxia-related factors (HIF-1α, VEGF), presenting a promising strategy for persistent type I X-PDT.

In a study, low-dose X-PDT using purely organic phosphorescent nanoscintillators, offering a safer alternative to traditional therapies that rely on heavy-metal-containing agents was developed 91. These nanoscintillators serve both as scintillators and PS, effectively generating ^1^O_2_ through energy transfer when exposed to X-rays. This approach enhances deep-tissue cancer treatment by combining the strengths of PDT and RT, while minimizing adverse effects on healthy tissues. The low dosage of 0.4 Gy used in this therapy demonstrates significant efficacy in treating deep tumors, highlighting its potential for broader applications. A novel UV PL material, particularly Bi and Sb co-doped LaGaO_3_, capable of ultra-long UV emission lasting over 2000 h was developed 92. These materials, activated by X-rays, exhibit high energy collection and storage efficiency due to oxygen vacancies acting as energy traps within the perovskite structure. The UV emission peaks at 372 nm, making these materials highly effective for various applications, including X-ray imaging, phototherapy, and photocatalysis. A key application is in PDT, where the LaGaO_3_,Sb@g-C_3_N_4_ platform shows excellent *in vitro* and *in vivo* results using low-dose X-ray irradiation (0.51 Gy), overcoming traditional tissue penetration challenges while significantly reducing X-ray dosage.

This study presents a non-invasive X-PDT strategy combined with real-time NIR-II imaging for bladder cancer treatment. The system uses engineered nanotransducers with lanthanide-doped nanoscintillators, which convert X-ray energy into visible light to activate PSs for PDT, while simultaneously emitting NIR-II signals for deep-tissue imaging. Tumor-targeting peptides help these nanotransducers selectively accumulate in tumors, enabling efficient treatment. Under X-ray irradiation, this approach achieves strong tumor regression, reduced recurrence, improved survival, and restoration of immune balance. Importantly, NIR-II imaging allows continuous monitoring from diagnosis to treatment and prognosis, and enables on-demand, fractionated therapy by adjusting radiation dose based on imaging signals 93.

A pure organic aggregation-induced emission (AIE) nanoscintillator (TBDCR NPs) was developed that can directly absorb X-rays and efficiently generate both type I •OH and type II ROS without external energy converters. The incorporation of heteroatoms improves X-ray absorption and promotes excited-state processes, enhancing ROS production, while the AIE property and a rigid PEG shell further boost efficiency. This system showed strong fluorescence, deep tumor penetration, and high therapeutic performance, effectively suppressing both regular and radio-resistant tumors *in vitro* and *in vivo*. Overall, this strategy introduces a new class of pure organic nanoscintillators for efficient, hypoxia-tolerant X-PDT 94. A biocompatible organic nanoscintillator (BPT-HOF@PEG) for X-PDT was developed to treat hepatocellular carcinoma (HCC). This nanoplatform uses a hydrogen-bonded organic framework that acts as both a scintillator and PS, directly converting X-ray energy into ROS. Upon X-ray irradiation, it produces strong oxidative damage to cell membranes and mitochondria, while X-rays also cause direct DNA damage, leading to enhanced tumor cell death. This dual action significantly improves treatment outcomes, achieving strong tumor suppression *in vivo*. Mechanistically, the therapy promotes apoptosis and inhibits tumor growth pathways such as NF-κB, MAPK, and TNF signaling 95. **Table 1** shows a list of deep tissue PDT nanosystems and their experimental parameters in animal models.

### TME modulating PDT

TME is highly dynamic and is mildly acidic (pH ~6.5–6.9) due to high lactate production from altered cancer metabolism and poor blood supply. This acidity increases with distance from blood vessels and is more pronounced in hypoxic regions. Low pH promotes tumor growth by inducing abnormal vasculature, activating matrix-degrading enzymes, and enhancing invasion. It also damages nearby normal cells while tumor cells adapt, and drives immune suppression by polarizing macrophages toward a tumor-supportive phenotype 96-99. GSH levels are higher in tumor cells than in normal tissues, typically in the millimolar range (about 5–10 mM), while normal cells have lower levels (around 1–5 mM) 100, 101. ROS, particularly hydrogen peroxide (H₂O₂), are present at much higher levels in tumor tissues than in normal cells. Tumor cells generate excess ROS through altered metabolism, mitochondrial activity, and enzyme systems such as NADPH oxidase, with concentrations reaching up to ~100 μM—far higher than the low nanomolar levels in normal tissues 102. This elevated ROS environment supports tumor development, progression, and metastasis, and also contributes to immune suppression by influencing macrophage behavior. At the same time, cancer cells adapt to this oxidative stress and rely heavily on antioxidant systems for survival 103. Matrix metalloproteinases (MMPs) are zinc-dependent enzymes that degrade extracellular matrix (ECM) components and regulate tissue structure and signaling 104. In normal tissues, their activity is very low and tightly controlled, appearing mainly during physiological processes such as wound healing and angiogenesis. In contrast, within the TME, MMP activity is significantly elevated due to an imbalance between proteases and their inhibitors (TIMPs), leading to excessive ECM breakdown. MMP-9 is strongly upregulated across many cancers compared to normal tissues, whereas MMP-2 shows variable expression. Both enzymes degrade type IV collagen in basement membranes, promoting tumor invasion and metastasis. Overall, elevated MMP activity in the TME drives ECM breakdown and cancer progression, unlike the tightly controlled activity in normal tissues 105, 106.

Hypoxia is a key feature of solid tumors, where oxygen levels are much lower than in normal tissues due to rapid tumor growth and abnormal blood vessel formation. While normal tissues typically have oxygen levels around 5% (≈38 mmHg), tumors often show much lower values, sometimes dropping to 0.3–2.2% (≈2–17 mmHg), with even more severe oxygen depletion in tumor cores. This low-oxygen environment arises from poor and irregular blood flow, which limits oxygen delivery and diffusion. In response, tumor cells adapt through hypoxia-inducible factors (mainly HIF-1), which regulate many genes involved in metabolism, angiogenesis, and survival. These adaptations help cancer cells thrive under stress but also promote aggressive behavior, metastasis, and resistance to PDT 107-109. Hypoxia is a vital factor in the TME that promotes metastatic progression, and hence leads to poor survival in several tumor types. Alleviating hypoxia can be an attractive strategy for improving the diminished PDT efficacy, for instance, hyperbaric oxygen therapy (HBOT) increases the amount of oxygen a patient breathes by raising ambient pressure, which leads to more oxygen dissolving in the blood plasma and reaching tissues 110. In PDT, this can enhance treatment by supplying more oxygen for ^1^O₂ production, as long as the oxygen can effectively diffuse to the illuminated tumor region 111. However, systemic approaches like HBOT are not the only way to improve oxygen availability. An alternative strategy is to generate oxygen directly within the tumor. Nanozymes, which are nanomaterials with enzyme-like activity, can mimic catalase and convert endogenous hydrogen peroxide (H₂O₂) into oxygen (2 H₂O₂ → 2 H₂O + O₂). This approach links oxygen production to the tumor’s own redox environment, although its effectiveness depends on the availability of H₂O₂ and the stability of the nanozyme in biological conditions 112-114.

Another widely explored method involves oxygen carriers. Perfluorocarbons (PFCs) can dissolve large amounts of oxygen physically, with oxygen loading and release driven by partial pressure gradients 115-117. Because of their high oxygen solubility, PFC-based nanoemulsions or nanodroplets can act as efficient oxygen reservoirs for PDT 118. In contrast, hemoglobin-based systems rely on reversible oxygen binding, similar to natural red blood cells, and release oxygen according to physiological dissociation behavior, potentially enabling more controlled and biologically relevant oxygen delivery 119, 120. Finally, gas-filled microbubbles or nanobubbles provide a distinct approach by storing oxygen in a gaseous core. These systems can be externally triggered—most commonly using ultrasound—to release oxygen at a specific time and location 121, 122. This allows precise spatiotemporal control of oxygen delivery, making them particularly useful for synchronizing oxygen supply with PDT irradiation 123, 124. This section reviews NP-based strategies that counter hypoxia to elevate the effects of PDT.

HA, a glycosaminoglycan, is a principal component of ECM that participates in the proliferation, invasion, and metastasis of cancer cells 125, 126. Hyaluronidase (HAdase) is an enzyme that degrades HA, and has been used to improve the permeability of anticancer drugs 127, 128. A novel strategy adopted by Gong *et al.* used HAdase to alleviate hypoxia and improve the efficacy of PDT. Their results demonstrated increased microvascular densities that amplified tumor perfusion to relieve hypoxia. Consequently, improved tumor oxygenation and accumulation of photoactive micelles by the improved EPR effect resulted in PDT-mediated tumor-killing effects in a 4T1 tumor model 129. PDT that leverages ROS faces challenges in the hypoxic TME, where oxygen scarcity limits its efficacy. To address this issue, two NO-releasing zinc(II) phthalocyanines, ZnPc-2NO and ZnPc-4NO, were developed 130. These compounds interact with intracellular GSH to release NO, which inhibits mitochondrial respiration, reducing oxygen consumption and alleviating hypoxia. Both conjugates generate ROS upon light irradiation, exhibiting cytotoxicity under both normoxic and hypoxic conditions. Remarkably, ZnPc-2NO triggers ICD, releasing damage-associated molecular patterns (DAMPs) that stimulate dendritic cells (DC) maturation and anti-tumor immune responses. *In vitro* and *in vivo* experiments have confirmed ZnPc-2NO-mediated PDT as an oxygen-efficient strategy that suppresses tumor growth and improves anti-tumor immunity.

**Figure 4:**
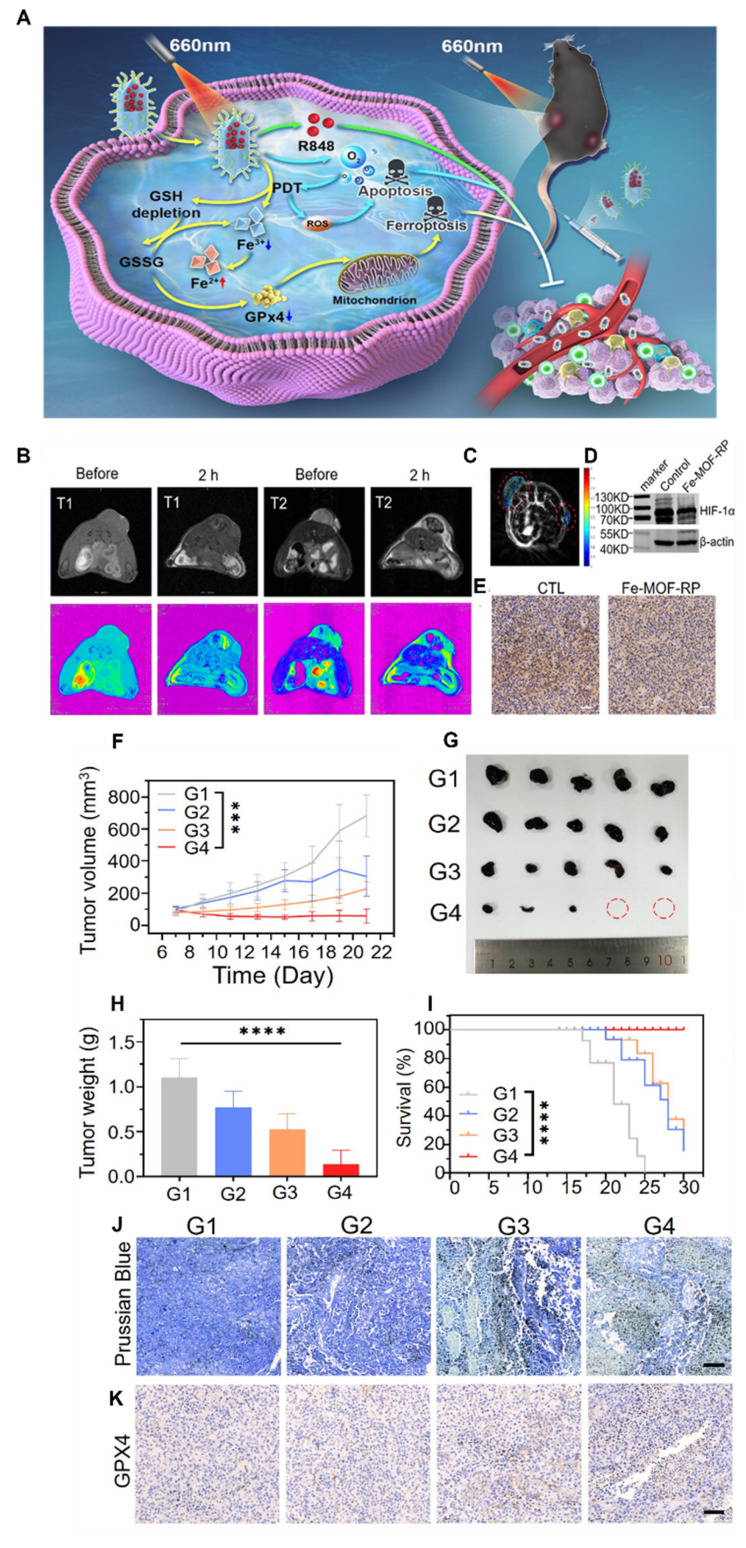
**(A)** Schematic Illustration of mechanism of the light-driven Fe-MOF-RP for efficient tumor therapy, **(B)** MRI imaging of tumor before and after the injection of Fe-MOF-RP, **(C)** Blood oxygen saturation measured by photoacoustic tomography after intratumoral injection of Fe-MOF-RP into the left tumor, **(D)** Western blot analysis, **(E)** immuno-staining of HIF-1α in the tumor. *In vivo* anti-tumor effects of Fe-MOF-RP, **(F)** tumor growth curves of different groups, **(G)** photographs of isolated tumors, **(H)** tumor weights after treatment, **(I)** survival rates, **(J)** iron content in tumor tissues assessed by Prussian blue staining, and **(K)** GPX4 expression in tumor tissues evaluated by immunohistochemistry. Adapted with permisision from 131, Copyright 2024 American Chemical Society.

A novel photodriven nanozyme, Fe-TCPP-R848-PEG (Fe-MOF-RP) was developed to address the immunosuppressive TME through a synergistic approach combining photodynamic, chemodynamic, and immunotherapy 131. Using Fe-TCPP metal-organic frameworks (Fe-MOFs) as both catalytic cores and delivery vectors for the immune agonist R848, the nanozyme enables precise tumor targeting and prolonged circulation (**Figure 4**). It catalyzes the decomposition of hydrogen peroxide at tumor sites to release oxygen, thereby alleviating hypoxia and improving PDT. Concurrently, ferroptosis induced by the Fenton reaction promotes the release of tumor-associated antigens (TAA), triggering ICD and robust anti-tumor immune responses. R848 further stimulates DC maturation and reprograms tumor-associated macrophages (TAMs), reshaping the TME. A recent study synthesized a carbon dot-based bifunctional nanosystem, MnZ@Au, designed to improve PDT by addressing tumor hypoxia and high glucose metabolism. MnZ@Au consists of Mn-doped carbon dots (Mn-CDs) as the core, a ZIF-8 shell, and ultrasmall AuNPs on the surface and acts as both a PS and a cascading nanozyme with glucose oxidase (GOx)-and CAT-like reactivity 132. It catalyzes glucose consumption and hydrogen peroxide generation, which triggers oxygen production to alleviate hypoxia and improve PDT efficacy. *In vitro* and *in vivo* studies have demonstrated enhanced tumor penetration, improved ROS accumulation, and significant tumor growth inhibition in breast cancer models. Furthermore, MnZ@Au enabled PAI and PET imaging to monitor oxygen saturation and reduced glucose uptake in tumors, respectively, thus validating its real-time catalytic activity and therapeutic effects.

A novel light-responsive nanoplatform for targeted PDT in pancreatic ductal adenocarcinoma (PDAC) was developed to address its dense desmoplastic and immunosuppressive TME. The system uses semiconducting polymer NPs (PCP-NPs) modified with midkine-specific nanobodies (MDK Nbs) to achieve precise delivery to PDAC tissues enriched with MDK expression 133. Upon light irradiation, the nanoplatform generates ROS at the tumor site, inducing apoptosis and ICD via ER stress and mitochondrial dysfunction. This process reprograms the TME by promoting DC maturation, T-cell infiltration, and cytokine release. The integration of PDT with programmed cell death protein 1 (PD-1) checkpoint blockade amplifies anti-tumor efficacy, achieving maximal tumor growth inhibition and extended survival in mice. TCPP-TER-Zn@RSV nanosheets (TZR NSs), a two-dimensional nanoplatform, were designed to enhance the efficiency and immunogenicity of PDT while addressing the immunosuppressive TME 134. TZR NSs combine an ER-targeting PS with resveratrol (RSV), which promotes autophagy and inhibits indoleamine-(2,3)-dioxygenase (IND). Upon laser irradiation, TZR NSs generate ROS in the ER, inducing oxidative stress. This process triggers ICD, releasing DAMPs and promoting DC maturation. RSV further regulates T cell abundance, increasing the proportions of CD8^+^ and CD4^+^ T cells while reducing immunosuppressive Foxp3 regulatory T cells, thereby reversing immunosuppressive TME and boosting anti-tumor immunity.

Dai *et al.* synthesized T-NPCe6-L-N, a tumor-targeted self-illuminating supramolecular NP designed to address the limitations of conventional PDT 135. By integrating the PS Ce6, luminol, and NO donor SNAP into a self-assembled NP, this system leverages high levels of tumor-specific H_2_O_2_ to trigger chemiluminescence resonance energy transfer, enabling self-excited PDT without external light. The selective activation of Ce6 in H_2_O_2_ and Fe^3+^-rich cancer cells ensures minimal side effects on healthy tissues, whereas NO release alleviates tumor hypoxia and depletes intracellular GSH levels, further amplifying the production of ROS. This dual mechanism effectively suppresses subcutaneous, deep-seated, and metastatic tumors while simultaneously inducing ICD for sustained anti-tumor immunity. Gao *et al.* developed TDR848@FPB, a TME-responsive antigen-capturing nanoplatform designed for systemic administration. A key point for TME modulation is that the platform is selectively activated by peroxynitrite (ONOO⁻) in the tumor, where it generates quinone methide traps that covalently capture TAA released after therapy. Upon light irradiation, the AIE-based PS produces strong ¹O₂, induces ICD, and releases R848, a TLR7/8 agonist, which helps turn the TME into a more pro-inflammatory and immune-active state. Importantly, this system not only enhances dendritic cell maturation and T-cell activation, but also actively reprograms suppressive immune cells in the TME, including converting M2 macrophages toward the anti-tumor M1 phenotype and shifting myeloid-derived suppressor cells toward antigen-presenting behavior 136. This study by Attar *et al.* presents a tumor-targeted self-illuminating nanoplatform (T-NPCe6-L-N) that improves PDT by combining internal light activation and TME modulation. Instead of relying on external light, the system uses high tumor H₂O₂ levels to trigger chemiluminescence (via luminol), which activates the PS (Ce6) through energy transfer, enabling effective PDT even in deep tumors. At the same time, the release of NO plays a key role in TME modulation by reducing hypoxia through vascular normalization and lowering intracellular GSH levels, which weakens tumor antioxidant defenses and enhances ROS generation. This dual strategy allows selective tumor killing, as activation mainly occurs in cancer cells with high H₂O₂ and Fe³⁺ levels, minimizing damage to normal tissues. Additionally, the treatment induces ICD, leading to sustained immune responses and long-term tumor suppression, including effects on metastatic tumors 137.

Photodynamic “gel-bombs” (DCM@OPR) was designed to improve cancer therapy by enhancing drug delivery and modulating the TME. These gel structures carry a PS (Ce6), MnO₂ NPs, and doxorubicin within a calcium-crosslinked matrix. Upon light irradiation, they generate ROS, producing explosive energy that breaks the gel into small fragments, allowing deep penetration into tumor tissue through physical gaps and receptor-mediated transcytosis. This mechanism ensures better distribution of therapeutic agents inside tumors. Importantly, MnO₂ reacts with tumor H₂O₂ and acidity to generate oxygen, helping to relieve hypoxia—a key TME barrier—and thereby improving PDT efficiency. At the same time, released Mn²⁺ and doxorubicin can activate immune pathways such as stimulator of interferon genes (STING), while excess Ca²⁺ promotes tumor cell death 138. Zhao *et al.* developed a TME-responsive biomimetic nanoplatform (FBFO@HM@aOPN) designed to improve the combination of PDT and immunotherapy for GBM by actively modulating the TME. After systemic injection, the platform accumulates in the tumor and responds to the acidic TME, where it promotes vascular normalization and ECM remodeling, enabling the NPs to penetrate more effectively. At the same time, its nanozyme core generates oxygen to relieve hypoxia, which enhances PDT efficiency. Importantly, the system also targets TAMs, converting them from a pro-tumor state to an anti-tumor M1-like phenotype, while also inducing immunogenic tumor cell death and ferroptosis, which increases neoantigen release. These combined effects strengthen both innate and adaptive anti-tumor immunity, making the tumor more responsive to immune checkpoint therapy such as anti-PD1, and leading to stronger tumor suppression and reduced recurrence 139.

Xiaohui *et al.* synthesized a biomimetic nanosystem (G-IrC8) that combines targeted PDT with TME modulation for stronger anticancer effects. The system was designed by loading a flexible-chain iridium PS (IrC8) into giant plasma membrane vesicles (GPMVs) derived from tumor cells, which improves biocompatibility and homologous tumor targeting. Once inside tumor cells, IrC8 preferentially accumulates in the mitochondria, where light activation triggers strong ROS production and efficient tumor cell killing. Importantly, the treatment induces ICD, which reprograms the TME by promoting antitumor immune activation 140. A TME-responsive small molecule (Ir-Fc) was designed to improve cancer treatment by combining PDT and ferroptosis through effective TME modulation. The molecule contains an acid-sensitive imine bond that breaks in the acidic lysosomal environment of tumors, releasing two active parts: Ir-NH₂, which works as a strong PS producing type I and type II ROS, and Fc-CHO, which drives Fenton reactions to generate highly toxic •OH and trigger ferroptosis. Importantly, this process helps overcome two major TME barriers—high GSH and hypoxia—because ferroptosis and Fenton chemistry consume GSH and also contribute to oxygen generation, both of which enhance PDT efficiency. As a result, Ir-Fc not only kills tumor cells directly but also reprograms the redox and oxygen balance of the TME, creating a self-reinforcing therapeutic effect with strong anti-tumor activity and good safety 141.

A hypoxia-responsive nanotheranostic (NanoPcN8O) was developed to address the oxygen-deficient TME. This system was based on a hydrophilic phthalocyanine that self-assembles into stable NPs and becomes activated specifically in hypoxic tumor regions through bio-reduction, converting into an active form (NanoPcN8). This transformation switches on type I photodynamic activity, generating oxygen-independent radicals, along with a photothermal effect, enabling effective tumor killing even under low oxygen conditions. Importantly, this design allows selective activation within the hypoxic TME, as confirmed by targeted PAI, thereby minimizing damage to normal tissues. The system shows significantly higher ROS generation under hypoxia compared to conventional PSs and achieves strong tumor growth inhibition *in vivo* 142. A dual-mode nanotherapeutic system (HAP@BMPns) was developed to overcome TME limitations and improve PDT outcomes. This system takes advantage of the acidic TME, where the hydroxyapatite-based nanomaterial breaks down after entering tumor cells, releasing Ca²⁺ and the PS. The increased intracellular Ca²⁺ causes mitochondrial damage and triggers apoptosis, representing a biological effect, while simultaneous activation of type I PDT under 800 nm irradiation generates oxygen-independent reactive species for photochemical tumor killing. This coordinated response effectively modulates the TME by exploiting its acidity and inducing mitochondrial stress. As a result, the combined therapy shows much stronger tumor inhibition, with significant reduction in cell survival and improved efficacy in both cell and animal models 143.

A NIR-activated, heavy-atom-free PS system (CHL) that improves cancer treatment through both phototherapy and TME modulation was recently developed. The PS Cy-BF was packed into phospholipids and platelet exosome vesicles, which improved tumor targeting and enabled 760 nm light-activated PTT plus type I PDT, even under hypoxic conditions. After activation, CHL caused mitochondrial damage and ICD, while also reducing tumor lactate production, an important metabolic factor in the TME. This metabolic shift helped remodel the immune environment by decreasing regulatory T cells (Tregs) and increasing CD8⁺ T cells. To further overcome lactate-driven immune suppression, lithium carbonate was added so that lactate could be repurposed as an energy source for CD8⁺ T cells, further strengthening anti-tumor immunity 144. A lactate-fueled self-acting PDT nanosystem for triple-negative breast cancer that works by both killing tumor cells and modulating the TME was introduced. The platform combines a PS (HPPH), luminol, and lactate oxidase (LOx) inside hollow MnO₂ NPs coated with hyaluronic acid for tumor targeting. LOx first consumes excess tumor lactate, converting it into pyruvate and H₂O₂, which helps reduce the harmful lactate-rich, immunosuppressive TME. The generated H₂O₂ then triggers luminol chemiluminescence, which activates HPPH without external light, allowing self-illuminated PDT. At the same time, MnO₂ reacts in the acidic tumor environment to generate oxygen, helping relieve hypoxia, a major barrier to PDT. Mn²⁺ formed during this process further enhances the reaction and also enables MRI tracking 145.

### Image-guided PDT

NIR FLI-guided PDT is promising for detecting and treating deep tumors, but its effectiveness is often reduced by dye aggregation and the trade-off between fluorescence and ROS production. To circumvent this, a pentamethine cyanine dye (C5T) was engineered using a specially designed triphenylphosphine counterion with an oligoethylene glycol chain. This design improves the dye’s balance between hydrophilic and hydrophobic properties and strengthens interactions within the system, preventing aggregation. As a result, the modified NPs (C5T-Pco) show strong NIR-II fluorescence and efficient type I ROS generation, both of which can be tuned by changing the excitation wavelength. Compared to standard dyes, this system provides deeper tissue imaging, higher ROS production, and better tumor targeting, including mitochondrial localization 146.

A series of Au(I)-based complexes were designed by combining Au units with special ligands, enabling a multifunctional system that integrates chemo/photo and immunotherapy. Among the series of synthesized complexes, one optimized complex showed strong tumor-specific imaging ability, including organelle-level targeting such as the endoplasmic reticulum, allowing image-guided therapy with better precision. Its anticancer effect originates from multiple coordinated actions, including inhibition of thioredoxin reductase, high ROS generation, and induction of ICD, which activates immune responses through stress signals and release of damage-associated molecules. Both *in vitro* and *in vivo* studies confirmed that this system can effectively treat tumors through image guidance, demonstrating strong theranostic potential 147. A pentavalent bismuth-based nanoplatform (NaBiVO₃-PEG) for cancer treatment that can generate ROS without requiring external light, oxygen, or hydrogen peroxide. In the acidic tumor environment, the NPs undergo hydrolysis, leading to continuous production of •OH and ¹O₂ through Bi(V) to Bi(III) conversion and lattice changes. Concurrently, sodium ion release triggers pyroptosis and promotes strong immune activation, enabling both direct tumor killing and systemic anti-tumor immunity, including effects on distant tumors and metastasis. Importantly, after i.v. administration, these NPs accumulate effectively at tumor sites and can be monitored in real time using CT imaging, enabling image-guided therapy. The system also combines immunotherapy and RT, showing strong tumor inhibition 148.

Treating deeply located GBM is difficult, especially when both accurate imaging and effective therapy are needed at the same time. To address this, a tumor-targeted europium hexaboride nanoplatform (EuB₆@RGD-K) was developed, where the RGD-K peptide enables specific binding to αvβ3 receptors on GBM cells. This system acts as a theranostic agent by combining MR imaging with NIR-II (1064 nm) and NIR-III (1550 nm) light-triggered phototherapy. It allows clear tumor visualization and real-time monitoring of treatment, enabling image-guided therapy with high precision. Upon light activation, the NPs generate ROS and heat, producing combined PDT and PTT effects. The long-wavelength NIR light enables deeper tissue penetration and non-invasive treatment, reducing damage to surrounding brain tissue. In animal studies, this approach significantly improved survival, especially with NIR-III irradiation. Overall, this platform demonstrates a powerful image-guided strategy for precise diagnosis, monitoring, and treatment of aggressive brain tumors 149. A theranostic nanoplatform (CDSP NPs) was developed by combining carbon dots with a paclitaxel prodrug. These NPs provide strong NIR afterglow imaging, deep tissue penetration, and high signal clarity, enabling real-time, image-guided surgical navigation for precise tumor removal. In addition to imaging, CDSP NPs deliver combination therapy by generating ROS for PDT and releasing paclitaxel for CMT, leading to enhanced tumor suppression 150. A smart dual-activatable nano-immunomodulator (DIR NPs) was designed for cancer treatment using NIR-II FLI-guided photodynamic immunotherapy. The NPs remain dormant during bodily circulation but become activated in the TME under 808 nm laser irradiation. Upon activation, they release a PS (DIR) and R848. The released DIR binds to tumor-associated proteins, which restores strong NIR-II fluorescence for clear tumor imaging and enhances ROS production for effective PDT. The released R848 boosts immune responses by promoting dendritic cell activation and subsequent T-cell-mediated tumor killing. This combined effect not only destroys primary tumors but also suppresses distant tumors and metastasis. 151.

A NIR-II phototheranostic platform based on a mitochondria-targeting moiety (MYM) that combines imaging and therapy for GBM treatment was designed and developed. To improve delivery, MYM was loaded into exosomes and modified with the iRGD peptide, allowing it to cross the blood–brain barrier and selectively accumulate in tumor. The system enables multimodal NIR-II imaging, providing real-time visualization of tumor location and treatment progress, supporting precise image-guided therapy. Upon 808 nm laser irradiation, MYM@iRGD-Exo exhibits phototherapeutic effects, leading to strong tumor inhibition and also enhances immune responses by promoting T-cell infiltration and activating immune-related pathways, as confirmed by RNA analysis 152. This study presents a smart nanoplatform (CDZP NPs) for breast cancer that combines sensitive miR-21 detection with enhanced PDT in a single system. Based on a ZIF-8 MOF, the NPs carry both an imaging module and a therapeutic module and release their components in response to the acidic tumor environment. The system uses a unique signal amplification strategy to detect very low levels of miR-21 with high sensitivity, enabling accurate tumor identification and real-time monitoring for image-guided therapy. The released Zn²⁺ activates DNAzyme that suppresses GPX4, reducing the cell’s ability to remove ROS, thereby boosting PDT effectiveness. 153.

To overcome the drug leakage issues from cell membrane coated NP delivery systems, Zhang *et al.* developed an electrostatically stabilized-light activated membrane delivery platform [Hm]@NPs for pancreatic cancer therapy. [Hm]@NPs was synthesized by the loading of AIE PS into a positively charged polymer with thioketal bonds vulnerable to ROS and coated is with red blood cell and pancreatic cancer cell membranes. When irradiated with white light, the highly stable polymer generated ROS by PDT effects, which disintegrates both ROS-responsive polymer and hybrid membrane, enabling drug release. In a pancreatic tumor model, [Hm]@NPs preferentially accumulated in the tumor and suppressed tumor growth by FLI-guided PDT 154. Conventional fluorescence probes for early diagnosis of pancreatic cancer suffer from poor penetration and accuracy. Zhu *et al.* developed an enzyme-activatable high contrast fluorescence probe using AIE fluorophore (QM) amphiphilic peptides QM–HSP–CPP. It primarily consists of QM, a hydrophobic peptide vulnerable to the enzyme cathepsin E (CTSE) and a cell-penetrating peptide. CPP has the ability to modulate the molecular dispersion properties that masks the fluorescence of QM in bodily circulation and due to CTSE activity in the tumor tissues emanates strong fluorescence. QM–HSP–CPP achieved intraoperative diagnosis of human PC sections, tracking PC in heterotopic nude mouse models with high specificity and long-term tracking ability 155. Conventional PDT systems cannot produce type I and type II ROS simultaneously due to their oxygen dependence. Wang *et al.* synthesized AIE PS Pys-QM-TT with optimal electronic properties capable of generating both type I and II ROS with simultaneous FLI. Notably, the strong donor–π–acceptor (D–π–A) structure of Pys-QM-TT enhances the ICT effect, which promotes emission in the NIR region. In addition, this structure lowers the singlet–triplet energy gap, aiding the system generate ROS more efficiently. In addition, the pyridinium salt group helps the excited PS transfer electrons more easily, which further increases the production of type I ROS. *In vivo*, Pys-QM-TT demonstrated the capability as image-guided PDT agent for cancer 156.

### Image-guided PTT

In contrast to PDT, which has experienced almost 50 years of development, PTT, which uses heat generated from laser irradiation of light-absorbing agents, has only recently been confirmed to achieve promising photothermal ablation (PTA) of tumors. Nevertheless, there have been considerable attempts to explore PTT nanomaterials, with significant achievements obtained in recent years. To date, a large number of photothermal nanomaterials have been explored for PTT and can be classified into the following four categories: 1) noble metal-based materials (e.g., Au) 157, 158; 2) transition metal–based materials (e.g., Cu_2-x_S, FeS, MoOx and WS_2_) 159-162; 3) carbon-based materials (e.g., carbon nanotubes, graphene, and fullerenes) 163-165, and 4) organic nanomaterials (porphyrin, polypyrrole, and semiconducting polymer) 166, 167. Au nanorods exhibit much stronger NIR absorption and scattering than other Au nanostructures due to their shape and aspect ratio. Smaller nanorods are efficient light absorbers, while larger ones enhance scattering. Unlike spherical particles, they enable uniform heat generation across the entire structure. This makes them highly effective for photothermal applications 168-170. Carbon-based nanomaterials arise from the versatile bonding ability of carbon (sp, sp², sp³), leading to a wide range of structures such as carbon dots, fullerenes, graphite, carbon nanotubes (CNTs), and graphene derivatives 17, 171, 172. These materials are attractive for photothermal applications due to their strong light absorption, chemical stability, low density, and cost-effectiveness. Their heat generation originates from excitation and relaxation of π-electrons, enabling efficient light-to-heat conversion across a broad spectrum. Optical and thermal properties depend on factors like size, shape, layer number, and doping, as well as fabrication strategies (top-down or bottom-up). Porous and hierarchical designs further enhance light absorption by reducing reflection and promoting multiple light scattering. In biomedical applications, especially cancer therapy, CNTs and graphene-based materials are widely explored as photothermal agents, often combined with drug delivery, gene therapy, or catalytic functions to improve therapeutic outcomes 173, 174. Palladium-based nanomaterials, especially ultrathin nanosheets, are promising photothermal agents due to strong NIR absorption, high heat conversion, and good stability. Their size and shape can be tuned to optimize optical properties 175, 176. These features make them useful for cancer treatment and imaging applications 177.

Furthermore, phototherapy can be effectively combined with optical imaging diagnostic technologies, such as FLI and PAI to realize the integration of tumor diagnosis and treatment, which will significantly improve the efficiency and accuracy of tumor therapy. This section highlights and discusses the recent progress in optical imaging-guided PTT nanomaterials in biomedical applications, primarily comprising recent developments in different modalities of imaging-guided PTT, further advancements of NP-based PTT, and the potential of PTT in clinical applications in the near future.

### Optical imaging-guided PTT

With the continuous development of optical imaging technology, the biological imaging window has gradually expanded from the visible (Vis) light region (400–700 nm) to the first NIR region (NIR-I, 700–900 nm) and the second NIR region (NIR-II, 1000–1700 nm). When working in the NIR window, NIR light imaging can provide superior performance in spatiotemporal resolution, signal-to-noise ratio, and imaging depth than Vis light imaging. In the past few years, nanomaterial-based NIR-I imaging agents have been widely used in clinical settings, including clinical trials and animal experiments 178, 179. Recent studies on NIR-II have attracted considerable attention, due to the lower tissue absorption/scattering and autofluorescence effects and improved resolution and deeper tissue visualization compared with that of NIR-I imaging, which is expected to be used in the clinic in the future. Here, we introduce the latest progress in the development of NIR-I and II optical imaging agents, including FLI and PAI, which may help readers grasp the trends in development and act as a possible guideline for the future of optical imaging agents.

### NIR-I window

With significant efforts dedicated to developing FLI, NIR FLI agents are particularly useful in research and clinical applications, where operators can directly explore the kinetics of drug movement or identify the intraoperative tumor margin. However, NIR-FLI agents with the “always-on” mode, with high background signals inevitably result in ‘‘false positive” signals in the complex and dynamic biological milieu. Currently, activatable or smart’ FLI agents, capable of changing signals upon interaction with targets of interest, can significantly reduce the background fluorescence, while simultaneously meeting the requirements of absorption to generate heat for PTT. Du *et al.* reported an “on-off” GSH-activable photothermal agent biotin-cystamine-Cys-Lys (Cypate)-CB, which was fabricated by biotin, cypate, 2-cyanobenzothiazole (CBT) and disulfidelinked cysteine (Cys). In the presence of GSH, the disulfide bond of biotin-cystamine-Cys-Lys (Cypate)-CB is reduced to initiate the CBT-Cys click condensation reaction (intramolecular quenching) and self-assembly (intermolecular quenching) into NPs, resulting in strong quenching of their fluorescence due to the FRET effect. Meanwhile, photothermal efficacy of the cypate fluorophore increases significantly and its PTT efficiency on cancer cells and tumors improves significantly 180. In addition to the “on-off” mode, activatable FLI characterized by the imaging signals “off/on” switch is another design strategy 181. Huang *et al.* designed a smart “off-on” system of CyPT–AuNRs nanohybrids, which combined AuNRs, heptamethine cyanine dye (CyPT), and GSH sensitive linker of thiodiglycolic acid 182. Firstly, the system would “sense” the tumor by providing NIR fluorescence, simultaneously exhibiting quenching in normal cells, because the fluorescent CyPT is released from the CyPT-AuNRs due to the stimulation of GSH. Moreover, in this system, the free AuNRs recovered the photoconverted thermal effect and can be excited by a single 808 nm laser and generate PTT and PDT effects simultaneously.

Regarding fluorescence-guided phototherapy, it is well understood that light emission and photosensitization are competing processes. Because most conventional organic dyes with strong intermolecular π–π stacking, exhibit strong competition from nonradiative pathways when their molecules are used as solid films or in the aggregate state. Therefore, the aggregation-caused quenching (ACQ) effect severely restrains their application in nanomaterial systems, because they are often used as aggregates rather than in discrete states. This problem can be resolved by enhancing the AIE. Compared with the planar structure of ACQ molecules, AIE agents generally have rotor structures, which restricts their molecular movements in the aggregate state and avoids nonradactive decay 183, 184. Recently, Yang *et al.* designed an NIR AIE-active probe with a donor-acceptor-donor (D–A–D) structure, T-BDP, by the conjugation of boron-dipyrromethene (BODIPY) and TPA. The AIE effect is attributed to the nonplanar configuration and three freely rotatable benzene rings. Accordingly, the amphiphilic T-BDP could spontaneously self-assemble into NPs (T-BDP NPs), which exhibited good water solubility and strong fluorescence in the NIR region with a maximum peak at around 724 nm. Furthermore, the self-assembled AIE agent T-BDP NP possessed robust photothermal conversion efficiencies (PCEs) of 50.9%. Under irradiation with a single 635-nm laser, the T-BDP NPs could generate ROS and heat simultaneously, facilitating the ablation of cancer cells through photodynamic and photothermal effects 185. More recently, Tian *et al.* designed Ce6-conjugated AuNPs that demonstrated significantly improved one- and TP excitation fluorescence, ^1^O_2_ generation, and photothermal effects. The optimum 1PE and 2PE fluorescence intensities of aggregated Ce6-AuNPs improved by 24.2- and 47.0-fold compared with those of pre-quenched Ce6-AuNPs and by 3.9- and 8.5-fold compared with that of free Ce6. Because the ^1^O_2_ generation efficiency of aggregated Ce6-AuNPs is 13.4-and 2.3-times better than that of pre-quenched Ce6-AuNPs and free Ce6, respectively. The aggregated Ce6-AuNPs exhibited excellent PDT and PTT performance under 660-nm lasers (194 mW/cm^2^) applied below the skin tolerance threshold 184.

In addition to approaches that depend on fluorescence responses, photoacoustic (PA) techniques have been incorporated into the design of precise biomedical imaging systems. During the therapeutic process of PTT, contrast agents or tissues that interact with input light would convert some of the optical energy into heat, and then ultrasound waves would be generated due to the heat-induced thermoelastic expansion. Thus, PAI, which detects phonons instead of photons after light excitation, makes PA and PTT an ideal pair for seamlessly and synergistically combining into theranostics 186. Among photothermal agents, noble metal plasmonic nanostructures, such as Au nanomaterials, are well known to possess strong LSPR and improved PCE at the NIR light region. With numerous photothermal clinical trials currently in progress (e.g., NCT00848042 and NCT01679470), the use of Au nanomaterials in PTT has established the foundation for the development of clinical applications in the near future. They also provide good chemical stability and interesting physical properties, making them powerful agents in imaging-guided PTT. For instance, El-Sayed *et al.* described the simulation prediction of the absorption efficiency of various Au materials. They estimated that Au nanostructures have a low luminescence quantum yield and exhibit absorption efficiency that confers large light-to-heat conversion efficiencies for PTT 187. Accordingly, Chen *et al.* designed biodegradable Au nanovesicle (BGV) assemblies composed of poly(ethylene glycol)-b-poly(ε-caprolactone) (PEG-b-PCL)-tethered AuNPs, which demonstrated LSPR absorbance in the range of 650–800 nm. The PCE was high, reaching 37%, which can be attributed to the high absorption-to-extinction ratio of small AuNPs that convert optical energy into heat with high efficiency. Results showed that tumors on mice were completely eliminated after NIR laser irradiation. Remarkably, noble metal-based nanomaterials can also act as PAI agents, whereas contrast-enhanced PAI can be used to guide and monitor therapies 188, 189.

In addition to Au nanomaterials, semiconductor nanomaterials, such as chalcogenides, as well as metal oxides, are considered the most promising candidates for PAI-guided PTT. Researchers have developed several chalcogenides (e.g., Cu_x_S_y,_ 190, 191, and Ce-doped MoOx 192), all of which exhibited strong LSPR in the NIR region. Therefore, porphysome-based PTT constitutes a promising method for highly selective cancer treatment and monitoring. Furthermore, other researchers have developed efficient heating agents by constructing organic dye-based NPs. For instance, ICG 193 and IR dyes 194 have been used to combine with encapsulating polymer or protein for PTT. Among them, ICG is a Food and Drug Administration (FDA)-approved drug for a number of clinical imaging applications 195. Tong *et al.* developed an improved ICG-mediated PTT platform by loading ICG into porphyrin-based covalent organic framework NPs (ICG@COF NPs). The porous COF structure allows efficient ICG loading and enhances its photostability and light absorption, resulting in a high PCE (~56.7%) under 808 nm irradiation. The composite showed stronger heat generation and better performance in killing cancer cells. Both *in vitro* and *in vivo* experiments confirmed that ICG@COF NPs can effectively ablate tumors, with near-complete tumor regression observed in 4T1 tumor-bearing mice, while maintaining good biocompatibility and minimal toxicity 196. Several research groups have reported the use of conjugated polymers, including polyaniline 197 and polypyrrole-based NPs 198 for PTT due to their high PCE, excellent photostability, and good biocompatibility. Owing to the synthetic methods, polymer NPs could be designed to possess a configurable backbone and side chains, which endows them with tunable photophysical properties. In 2013, Levi-Polyachenko *et al.* designed a nano-PCPDTBT for PTT applications *in vitro* under NIR irradiation. Their pioneering work demonstrated that a donor-acceptor (D-A) structure endowed conjugated polymer NPs with a low band gap and ensured strong absorbance in the NIR window 199. Furthermore, a recent work by Pu *et al.* presented alternating D–A semiconducting polymers containing diketopyrrolopyrrole (DPP) moieties as the acceptor groups. Their study provided evidence of how to finely tune bandgaps by copolymerizing DPP with different electron-donating monomers.

**Figure 5:**
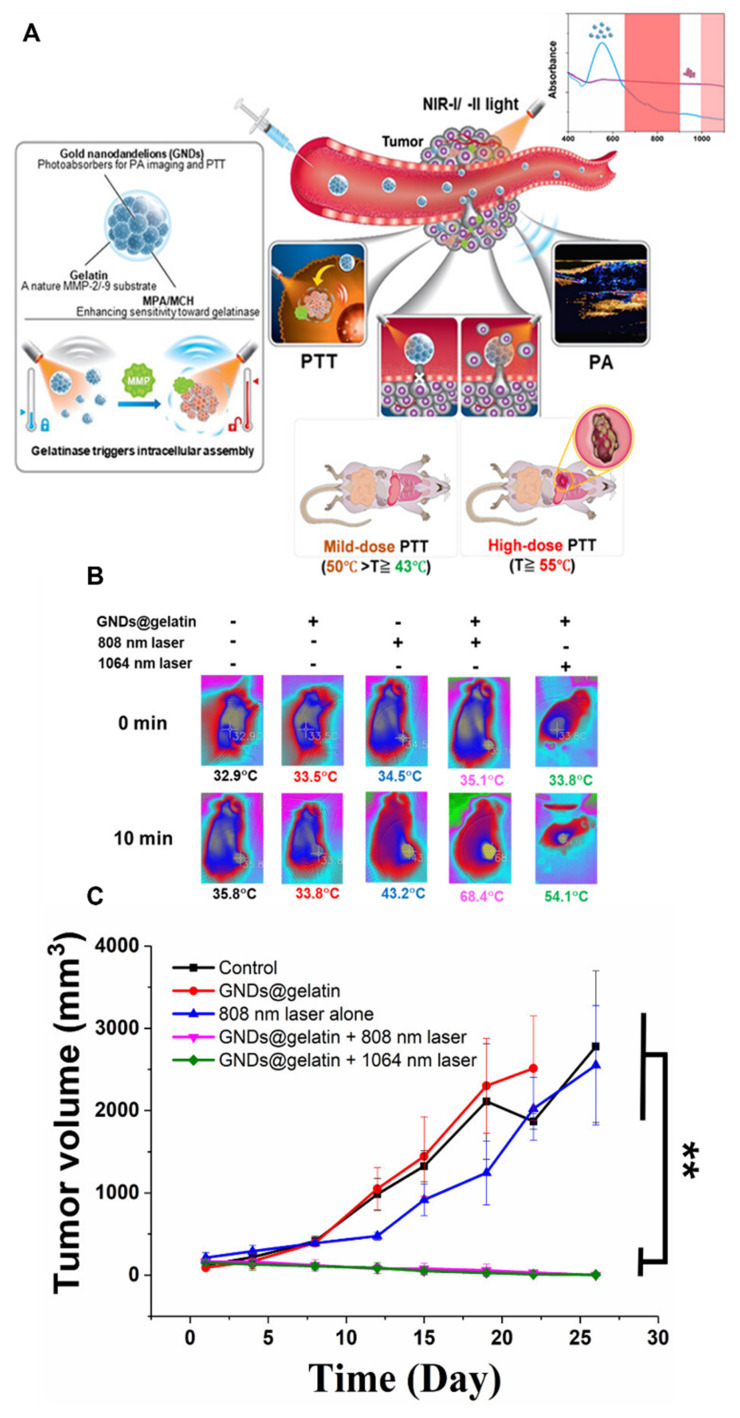
**(A)** Schematic of TME-responsive GNDs@gelatin and PAI-guided PTT and metastasis modulation. *In vivo* NIR-I and NIR-II PTT against tumors, **(B)** representative IR thermal images of C6 tumor-bearing mice injected with GNDs@gelatin and irradiated with an 808 nm (1 W·cm⁻²) or 1064 nm (1 W·cm⁻²) laser at different time points, and **(C)** tumor growth curves after various treatments over 30 days. Adapted with permisision from 202, Copyright 2024 American Chemical Society.

A Nd-Yb co-doped nanomaterial, termed water-heating NIR NPs, were developed for NIR imaging-guided PTT by leveraging the strong absorption of water at 1.0 μm^200^. The incorporation of Tm ions extended the NIR lifetime, enabling precise tumor visualization and targeted heating in deep tissues. In a GBM multiforme (GBM) mice model, anti-CD133-conjugated NPs (Ab-NPs) improved tumor targeting by 2.4 times compared to unconjugated NPs, leading to 78.9% tumor volume reduction under 808 nm continuous-wave laser irradiation. Systematic *in vitro* and *in vivo* studies confirmed effective brain tumor localization, deep-tissue imaging, and PTT, demonstrating the potential of these multifunctional NPs for intracranial cancer treatment with minimal toxicity. Fe²⁺-chelated PFC-encapsulated polyepinephrine (PFC@PEPP-Fe) nanoshells were designed as a theranostic platform for dual-mode imaging and synergistic cancer therapy 201. Structurally, the PFC core functions as an ultrasound (US) contrast agent and oxygen carrier, enhancing chemodynamic therapy (CDT) by self-supplying O₂ and H₂O₂, while the PEPP-Fe shell enables fluorescence (FL) imaging and PTT under NIR light. The NPs exhibit tumor-specific US imaging via microbubble generation and cancer-selective “OFF-ON” FL signals due to Fe²⁺ chelation. NP-mediated PTT can be optimized to prevent metastasis by precisely tuning photothermal temperature 202. A theranostic nanoplatform, GNDs@gelatin, was developed to selectively activate NIR-II photoacoustic image (PA)-guided PTT through MMP-triggered *in situ* assembly. Unlike conventional "always-on" PTT agents, GNDs@gelatin offers tumor-specific activation, precise PA-based treatment planning, and efficient dual-NIR (808 nm and 1064 nm) energy harnessing. Notably, transcriptome analysis revealed that high PTT temperatures (>55 °C) induced metastasis-related pathways, whereas moderate temperatures (43–50 °C) effectively ablated tumors without metastasis (**Figure 5**). This "moderate-is-better" approach demonstrated improved long-term survival in glioma models, highlighting GNDs@gelatin as a promising strategy for metastasis-free, tumor-targeted PTT. A multifunctional NIR-triggered phototheranostic agent, TTNH, was developed for dual-modal imaging and synergistic type-I PDT and PTT in hypoxic tumors. By integrating an electron-deficient 2-cyanothiazole unit, TTNH achieves efficient intersystem crossing, enabling superoxide radical (O₂•⁻) generation under 808 nm laser irradiation. TTNH NPs (TTNH NPs) exhibit strong NIR-II fluorescence (QY = 2.08%), excellent PCE (51.8%), and enhanced PAI capabilities. The NPs demonstrate long tumor retention (>120 h) and induce potent mitochondrial damage, promoting apoptosis in both normoxic and hypoxic conditions 203.

A multifunctional image-guided phototherapy nanoplatform that integrates fluorescence and photoacoustic (PA) imaging with therapy for precise cancer treatment was developed by Kang *et al.* The system is built using a near-infrared AIEgen combined with a hypoxia-responsive paclitaxel (PTX) prodrug, and further coated with a macrophage cell membrane to enhance tumor targeting. The strong optical properties of the AIEgen enable high-quality fluorescence and PA imaging, allowing accurate tumor localization and real-time guidance during treatment. Upon light irradiation, the platform generates ROS, inducing PDT and ICD, while also consuming oxygen and increasing tumor hypoxia. This hypoxic condition then accelerates the release of the PTX prodrug, creating a self-enhancing therapeutic cycle. As a result, the combined effects of imaging-guided therapy, PDT, and CMT trigger strong immune responses, effectively suppressing both primary and distant tumors 204. A TME- responsive, image-guided PTT strategy for HCC was developed by Zeng *et al.* It identifies ATP6V0C, a proton pump subunit, as a key driver of tumor acidity, which is closely linked to tumor growth, metastasis, and poor prognosis. To monitor this, a pH-responsive ratiometric photoacoustic sensor (PPS) was developed, enabling real-time, high-resolution imaging of tumor acidity during cancer initiation, progression, and metastasis. This imaging approach allows precise visualization of tumor boundaries and dynamic pH changes, improving early diagnosis and disease monitoring. Importantly, PPS also shows enhanced photothermal effects in acidic conditions, and when combined with a proton pump inhibitor, it achieves synergistic tumor suppression 205.

Shi *et al.* presented a NIR FLI-guided PTT strategy using a newly designed semiconducting polymer, PF2. By introducing fluorine into the polymer structure, the researchers improved both FLI ability and heat generation, overcoming the usual trade-off between imaging and photothermal performance. Compared with its chlorinated counterpart, PF2 NPs showed stronger NIR fluorescence, a high PCE of 69.8%, and better molecular properties such as improved planarity and light absorption. These features allowed clear tumor imaging and efficient tumor heating under 808 nm laser irradiation. In 4T1 tumor-bearing mice model, PF2 NPs achieved strong tumor suppression (about 80% inhibition) with good biocompatibility and safety 206. Zhao *et al.* developed a multifunctional, image-guided PTT nanoplatform, called ASNP NPs, to improve anti-tumor immunity. The system combines a NIR photothermal agent with sodium nitroprusside (SNP) inside heat-sensitive liposomes. Under NIR laser irradiation, ASNP NPs generate heat for PTT and simultaneously release SNP, which reacts with GSH to produce NO and Fe²⁺. The Fe²⁺ further reacts with H₂O₂ to generate •OH, promoting ferroptosis. This three-way treatment-PTT, NO gas therapy, and ferroptosis—greatly increases the release of DAMPs, which helps reprogram the tumor immune microenvironment and activate stronger tumor-killing immunity. In addition, the NPs show strong fluorescence, allowing better imaging guidance during treatment. In a 4T1 breast tumor model, this strategy significantly suppressed tumor growth 207.

### NIR-II window

As well documented over the past several decades that most photoinduced theranostics operate in the NIR-I window, numerous photoinduced agents in the traditional NIR window have been extensively investigated in both animal and clinical applications. Nonetheless, there remains much room for improvement. In particular, endogenous optical species, including Hb and deoxyhemoglobin, exhibit light absorption in the NIR-I region and, combined with light scattering within the same spectral region, have contributed to significant attenuation of excitation light, which in turn has led to a decrease in imaging sensitivity, spatial resolution, and tissue penetration depth. To further increase tissue penetration depth, techniques using longer wavelengths have also been developed to overcome the limitation of optical imaging depth. In 2009, Smith *et al.* proposed the second “transparent window” with a longer wavelength from 1000 to 1700 nm, which is also known as the NIR-II window 208. Compared with that in the NIR-I window, optical imaging in the NIR-II window can yield significantly deeper penetration depth, with good temporal and spatial resolutions and minimized autofluorescence 209. Using a single component, Ag_2_S QDs with a high fluorescence quantum yield (15%) in the NIR-II range have been reported to detect tumors with a high signal-to-background ratio through passive tumor targeting 210. Subsequently, both *in vitro* and *in vivo* NIR-II imaging results were achieved using Ag_2_S QDs, which were found to be attractive NIR-II fluorescence probes with good biocompatibility and high quantum efficiency. In addition to the individual NIR-II FLI function, Ag_2_S QDs can exhibit photothermal characteristics, which have been used for image-guided PTT 211-213. For instance, Liu *et al.* used the reversal of ACQ strategy to design a pH-responsive photothermal NP for FLI-guided PTT. In their work, the stimulation-responsive assembled Ag_2_S vesicle (Ag_2_S Ve) was proposed by the self-assembly of Ag_2_S QDs coated with pH-sensitive copolymer thiolated polystyrene-co-poly(4-vinylpyridine). The Ag_2_S Ve demonstrated strong fluorescence quenching in the second NIR-II region. Triggered by the acidic environment, transformation of Ag2S QDs from aggregated to disaggregated states promptly mediated the quenched NIR-II fluorescence from “off” to “on.” Due to these properties, the theranostic Ag_2_S Ve can be specifically activated in acidic tumor tissues, whereas it remains nonfunctional in normal tissues. Besides the above-mentioned types of photothermal agents, imaging-guided PTT was successfully realized in tumor-bearing mice using several different types of NIR-II absorbers, including semiconducting polymer NPs 214, lanthanide NPs 215, Au nanostructure 216, silicon oxide NPs (H-SiOx NPs) 217, semimetal NPs 218, metal carbide 219, etc. All these systems demonstrated efficient anticancer ability. Especially, semiconducting polymer NPs are not only capable of photothermal heating and fluorescence/PA imaging of tumors but also possess good biocompatibility and biodegradability, thus providing great promise for translational medicine.

**Figure 6:**
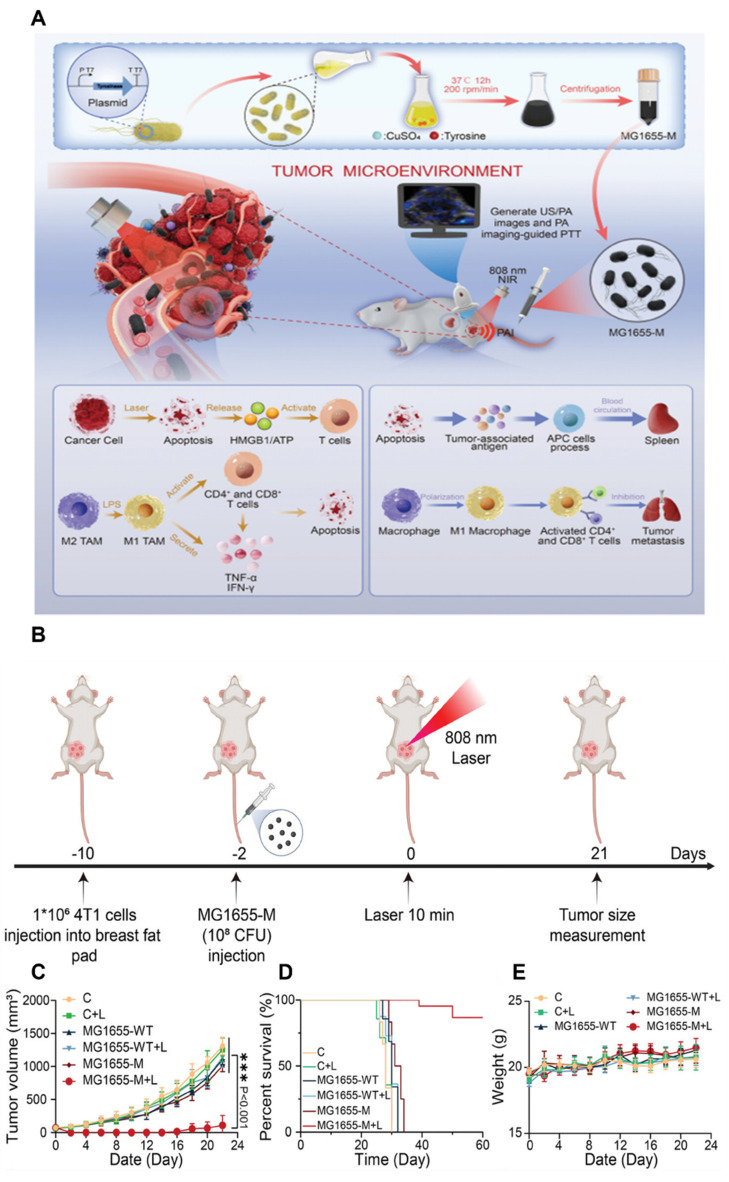
**(A)** Schematic of MG1655-M for photoacoustic image-guided photothermal therapy and its anti-tumor mechanisms, **(B)** schematic of MG1655-M-mediated photothermal immunotherapy in the 4T1 primary tumor model, **(C)** tumor growth curves in mice after different treatments, **(D)** survival curves of mice in each group, and **(E)** body weight curves of mice receiving different treatments. Adapted with permission from 220, Copyright 2024 John Wiley and Sons.

A novel approach to tumor treatment using genetically engineered *Escherichia coli* MG1655 (MG1655-M) that biosynthesize melanin NPs was developed 220. These bacteria target hypoxic tumor regions, where they effectively colonize and deliver melanin. This delivery can be tracked in real-time using PAI, allowing precise timing for PTT. Upon exposure to an 808-nm laser, the melanin induces localized heating, resulting in significant tumor ablation (**Figure 6**). Moreover, the PTT triggers strong anti-tumor immune responses, creating long-term immune memory that helps prevent tumor metastasis and recurrence. This integrated platform provides image-guided, tumor targeting therapy with combined photothermal and immune treatment, presenting a promising strategy for future cancer therapies. A novel hydrogen-based cancer therapy using diameter and shape-controlled magnesium (Mg) NPs, including hexagonal nanosheets, nanoflowers, and small NPs, were investigated. Among them, pH-sensitive polymer-coated Mg nanoflowers (MgNF@PEG/PMMVP) demonstrate significant potential in NIR-II PAI and bubble-enhanced ultrasound imaging (USI) for cancer treatment 221. These nanoflowers, approximately 100 nm in size, have a unique structure that allows strong NIR-II absorption, making them effective imaging contrast agents. When introduced into the acidic tumor environment, the polymer shell disassembles, causing a reaction between Mg and water that generates hydrogen (H₂) bubbles. These bubbles improve ultrasound imaging signals, induce cavitation that ruptures lysosomes, and disrupt cellular energy metabolism, leading to oxidative stress and cancer cell death. This drug-free method effectively combines bubble burst-like action with H_2_ therapy, resulting in significant tumor inhibition with minimal side effects and good biosafety. The approach holds promise for clinical applications due to its selective targeting and non-invasive nature.

However, unsatisfactory imaging depth and signal-to-background ratio remain intrinsic issues for clinical application. A few recent studies have explored the use of lasers in optical subwindows such as 1300–1400 nm (termed the NIR-IIa window) and 1500–1700 nm (termed as the NIR-IIb window) for the better performance of FLI. For instance, Wu *et al.* reported a PEG-stabilized copper sulfide NP (CuS NP) that possesses a broad absorption range, and was used as a PTT agent and excited by either 808- or 1275-nm laser. This multifunctional CuS NP not only serves as a contrast agent for NIR-IIa PAI but also functions as a photothermal agent for PTT. Moreover, the 1275-nm laser possesses deeper tissue penetration capability than the 808-nm laser. The authors also demonstrated that the temperature increments in the CuS-PEG NP solution irradiated by the 1275-nm laser at depths of 2, 5, 10, 15, and 20 mm were 10.5-, 9.1-, 6.5-, 6.2-, and 4.8-fold higher than those observed with the 808-nm laser 222. Besides, semiconducting polymer NPs (SPNs) have been widely used as NIR-II PAI or PTT. Zhang *et al.* used diketopyrrolopyrrole and benzobisthiadiazole (BBTD) as two acceptors and constructed D-A1-D-A2 semiconducting polymer NPs for NIR-IIa PAI-guided chemo-photothermal combination therapy of cancers. BBTD exhibits desperate electron-withdrawing capability in D-A polymers and decrease the bandgap to drive the absorption spectra to the longer wavelength. Therefore, their established two-acceptor semiconducting polymers (SP1-3) with the strongest electron deficient acceptor not only exhibited a high photothermal conversion efficiency (60%) at 1064 nm, but also as an excellent PAI agent with the strong photoacoustic signal at 1280 nm 223. Due to the lack of endogenous fluorescent molecules emitting beyond 1400 nm, the NIR-IIb window provides near-zero autofluorescence and negligible scattering, thus providing an optimal imaging platform for *in vivo* research 224, 225. To date, there have been several efforts toward the design and synthesis of nanomaterials with longer and brighter NIR-IIb imaging. For instance, Huang *et al.* designed a quantum-dot-based nanoprobe with broad NIR absorption and emission tailing beyond 1500 nm and used it for imaging-guided PTT 226. A novel small-molecule NIR-IIb dye IT-TQF with a D–A–D structure was synthesized by Cheng *et al.* The IT-TQF NP-based NIR-IIb imaging exhibited high spatial resolution and high tissue penetration depth, and the extended imaging wavelengths improved the tumor signal-to-background ratio to 9.42 in orthotopic osteosarcoma models. Importantly, the IT-TQF NPs displayed high PTT efficacy (PCE: 47%) for effective tumor treatment in mice 227. Recently, Wang *et al.* reported a TME-responsive hollowed virus-bionic MnO_2_ nanoshell (HvMnO_2_@QDs-IR1061), which loaded IR1061 in the cavity and anchored QDs (PbS@CdS) on the surface. The NIR-IIb fluorescent signal in the HvMnO_2_@QDs-IR1061 can be completely quenched in normal tissues via absorption competition-induced emission mechanism. Subsequently, the pH of the TME triggered NIR-IIb fluorescence recovery and exerted efficient NIR-II photothermal effect for lymphatic metastasis under NIR-IIb fluorescent imaging navigation 228. Despite the large number of strategies used for realizing NIR-IIb imaging-guided PTT, the major absorption peaks are concentrated in the NIR-I region. In general, high excitation power or exposure time is required in the tail NIR-II fluorescence bioimaging. Small organic molecules generally display low fluorescence brightness in water due to the lipid-soluble characteristics. Moreover, the major peaks of absorption/emission rarely reach beyond 1300 nm. Therefore, the development of high-brightness and long-wavelength emissive fluorophores remain a major research challenge.

NP-based PTT has been extensively investigated recently, and the development of depth-independent imaging-guided multifunction nanoplatforms has been well documented in several reviews. Being realistic, the maximum optical penetration depth achievable in human tissues is within the range of a few centimeters. Therefore, therapeutic light-triggered PTT is incompatible with depth-limitless imaging technology for preclinical applications and clinical translation. Instead, several research groups intend to explore novel light delivery systems to circumvent the major limiting issue of deep-tissue treatments. NP-based photothermal treatment of deep-seated tumors is still achievable if an optical fiber delivery is used in combination with depth-limitless imaging-guided techniques. Recently, Ma *et al.* 229 introduced transvascular interventional PTT (Ti-PTT), a minimally invasive approach that integrates endovascular delivery with PTT for precise tumor treatment. A small microcatheter (1.8 Fr) equipped with an ultrafine optical fiber (100 μm) was used to simultaneously deliver ICG-based photothermal agents and 808 nm laser irradiation directly through blood vessels. Two formulations were investigated: ICG solution for direct photothermal heating and ICG–ethiodized oil (ICG–EO) emulsion, which improves retention and acts as a photothermal embolic agent. Under laser activation, localized heat induces embolization, preventing blood supply and causing tissue necrosis even beyond the normal light penetration depth. The ICG–EO formulation showed better retention and more effective embolization than free ICG. In animal models, Ti-PTT achieved precise vascular occlusion and strong therapeutic effects, and its parameters can be adjusted based on vascular structure. Huang *et al.* developed a catheter-based iPTT system that combines real-time imaging and therapy using a single 1310 nm light source for precise treatment of orthotopic colorectal cancer (CRC). A miniaturized catheter (0.9 mm) with an optical fiber enables simultaneous optical coherence tomography imaging and PTT, allowing accurate tumor localization and controlled heat delivery. Folate-modified Bi/Bi₂S₃ NPs act as both imaging enhancers and photothermal agents, showing strong NIR-II absorption, scattering signals, and efficient heat generation due to localized surface plasmon resonance, along with effective tumor targeting. *In vitro* and *in vivo* studies demonstrated effective tumor ablation, high imaging contrast, and minimal toxicity, confirming good biosafety 230. To date, AuNP-based functionalized stents have been introduced for the local treatment of cancer cells or tissue hyperplasia adjacent to stented nonvascular luminal organs 231-233. For instance, Hu *et al.* developed a localized PTA therapy for obstructive rectal cancer, which operates through AuNP-coated stent with NIR irradiation. Aided by the excellent NIR absorption properties of branched AuNPs, the nanotherapeutic stents displayed effective suppression of tumor growth after stent placement by heat-induced tumor necrosis. In the same year, Cho *et al.* used the same concept in a rat esophagus model. Their results demonstrated that the AuNP-coated stent-mediated local PTT protocol could be used to treat not only granulation tissue formation but also tumor ingrowth or overgrowth through the stent meshes. Although further preclinical studies are required to investigate the efficacy and safety of localized heat treatment, the developed nano-functionalized SEMS and localized PTA therapy should be promising for clinical applications.

A multifunctional bionic nanoplatform (Au@MnO₂@PM; AMP) was developed for multimodal imaging and NIR-II PTT of lung cancer 234. The system integrates Au nano-bipyramids (Au NBPs) for photothermal conversion and MnO₂ for enhanced MRI, with a homologous cancer cell membrane coating for precise tumor targeting. AMP enables CT, photothermal imaging (PTI), and MRI, while achieving high photothermal conversion efficiency (52.07%) under 1064 nm irradiation. *In vitro* and *in vivo* studies confirmed effective tumor ablation with no significant systemic toxicity. The tumor-homing ability and microenvironment-responsive MnO₂ release further enhance imaging and therapeutic efficacy, highlighting AMP as a promising nanotheranostic for precise cancer diagnosis and treatment. A next-generation photothermal agent, Stealth NanoBomb (SNB), was developed to enhance mild-temperature (<45 °C) PTT by inhibiting HSPs expression in cancer cells. SNB consists of self-assembled small molecular NIR-II AIEgens and a CO carrier polymer (PLGA(CO)) coated with PEG-lipid, ensuring stability and safe circulation for intravenous administration 235. Upon tumor accumulation, SNB responds to the overexpressed H₂O₂ in the TME, releasing CO gas to suppress HSPs and enhance PTT efficacy. Compared to previous nanobomb generations, SNB offers improved safety, stability, and targeted CO gas/drug co-delivery, representing a promising strategy for more effective, low-toxicity cancer therapy. A novel small-molecule phototheranostic agent, CY-1234, was developed for NIR-II PAI-guided PTT with excellent biocompatibility and tumor-targeting capabilities 236. CY-1234, featuring an extended π-conjugation structure, exhibits strong NIR-II absorption at 1234 nm and is nanoencapsulated (CY-1234 NPs) for aqueous solubility and *in vivo* stability. These NPs achieve high photothermal conversion efficiency (76.01%) under 1064 nm laser irradiation, leading to efficient tumor ablation with a 97% apoptosis rate in HeLa cells. *In vivo* studies confirmed strong PA signals, effective tumor inhibition, and no systemic toxicity. A bismuth-doped iron selenide (BFS) NPs platform was developed to enhance CDT and second NIR-II PTT for CRC. BFS NPs exhibit high photothermal conversion efficiency (31.9%) and accelerate the Fenton reaction under NIR irradiation, increasing ROS generation and improving CDT efficacy 237. Additionally, bismuth doping enables T2-weighted MRI and CT imaging, providing precise guidance for NIR-II PTT. The synergistic CDT/PTT approach effectively inhibits tumor growth while minimizing side effects.

As discussed earlier, a large number of nanomaterials have been developed as NIR-absorbing photothermal agents to serve as heat carriers for local hyperthermia. Local hyperthermia in tumors can not only directly eradicate cancer cells but also exert additive effects that improve the effect of conventional PTT. For instance, PTT has been combined with CMT to compensate for each other’s limitations, e.g., poly(N-isopropylacrylamide) (PNIPAM), a well-known thermally responsive polymer in which temperature-dependent swelling and shrink phase transition behavior can drive actuated spatiotemporal drug release under NIR irradiation. Moreover, several research groups constructed numerous photo-responsive components for synergistic PTT/CMT, including PNIPAM derivative copolymer, poly(ε-caprolactone), and poly(vinyl alcohol) 238-240. In addition to the spatiotemporal- and dose-controlled delivery of CMT for improved therapeutic efficacy and reduced systemic side effects, thermal-sensitive materials were successfully realized in a biodegradable nanoplatform to accelerate NPs elimination from the living body. Specifically, the subunit thermal-sensitive polymer-tethered NPs self-assemble into a larger formulation. With laser irradiation, when the thermal-sensitive polymer exhibits structural damage or change, the building blocks dissociate into their original sizes, thereby facilitating their elimination from the body 241, 242. The degradation and clearance process of self-assembled formulation through the hepatobiliary and renal route was demonstrated, encouraging the translation of NP-based PTT to clinical applications 243, 244.

In addition to the direct efficacy of NP-based PTT, some studies have focused on the indirect influence of NP-based hyperthermia to tackle the surrounding TME sequentially and then the core tumor cells 245. Conversely, in terms of tumor vessels, local hyperthermia could be exploited for increasing the vascular permeability to result in better extravasation (100 nm) of NPs when the heating temperature range is 40 °C–42 °C. In contrast, the effect of PTT also influences tumor stroma in which temperature increase affects the collagen architecture and then remodels the ECM. For instance, the research groups of Bhatia and Gazeau confirmed that PTT may serve as a nano-heater to induce localized denaturation of collagen, especially in the study of Gazeau, through local denaturation of adjacent tissue. In particular, on collagen fibers, light-exposed multiwalled carbon nanotubes induce remodeling and softening of the TME concomitant to tumor regression 246, 247. Remarkably, the structural change of TME after local heating, which favored further penetration of NPs and drug, has the potential to improve the efficacy of current treatments 248. **Table 2** shows the** s**ummary of NIR I and II image-guided PTT nanosystems.

Jiang *et al.* developed an image-guided PTT nanomotor for treating superficial tumors through peritumoral subcutaneous injection. The nanomotor consists of a polydopamine (PDA)-coated spherical core loaded with ICG, allowing it to convert NIR-I light into both heat and NIR-II fluorescence. Under laser irradiation, the generated heat not only enables tumor-killing PTT, but also creates a thermophoretic driving force that actively pushes the nanomotor through subcutaneous tissue toward the tumor. At the same time, the NIR-II fluorescence provides real-time imaging guidance, allowing the movement of the nanomotor to be tracked and directed accurately. The heat also helps reduce tissue barriers by loosening fat and connective tissue, improving delivery efficiency 249. Shi *et al.* reported a NIR FLI-guided PTT platform based on a newly designed photothermal agent, 3TPA. The molecule was engineered using a CF₃-BODIPY core with triphenylamine (TPA) rotors, which enhances light absorption and promotes efficient heat generation through strong nonradiative decay, resulting in a high photothermal conversion efficiency (~57%). When encapsulated into a biocompatible polymer (DSPE-mPEG), the resulting 3TPA NPs (3TPA NPs) show improved tumor targeting, stability, and photostability compared to conventional dyes. Importantly, these NPs enable dual NIR-I/NIR-II fluorescence imaging, allowing precise visualization and guidance during therapy. Under 808 nm laser irradiation, 3TPA NPs rapidly raise tumor temperature to effective levels for ablation, leading to near-complete tumor elimination with no recurrence in animal models, while maintaining good biosafety 250. Patnaik *et al.* developed an Au-coated solid lipid NP (Au-SLN) as a promising image-guided PTT agent for breast cancer. The NPs showed very high photothermal conversion efficiency (about 80%), When exposed to laser, Au-SLNs rapidly raised tumor temperature to above 60 °C, enabling effective thermal ablation. Importantly, treatment response was monitored using multiple non-invasive imaging methods, including infrared thermography for real-time heat tracking, bioluminescence imaging (BLI) and NIR fluorescence for tumor activity, and microCT for anatomical and volumetric assessment. This multimodal imaging approach allowed precise evaluation of both treatment effectiveness and healing 251.

A smart image-guided phototherapy platform, called BOD-D was developed by Sun *et al.*, which can switch treatment modes during therapy to improve tumor killing. Initially, BOD-D works as a NIR-I FLI-guided PDT agent, producing ^1^O_2_ to destroy tumor cells. As treatment continues and the tumor becomes more hypoxic, PDT becomes less effective, so light activation triggers BOD-D to release NO and convert into BOD-T, a photothermal agent with NIR-II fluorescence. This allows the therapy to shift from NIR-I-guided PDT to NIR-II-guided PTT. Importantly, the released NO also acts as gas therapy and helps sensitize both PDT and PTT, making the overall treatment stronger 252. Chen *et al.* introduced a novel image-guided NIR-II PTT based photoimmunotherapy strategy using a membrane-anchoring small molecule, CBT-3, that directly disrupts cancer cell membranes. The molecule was designed to attach to tumor cell membranes and, under NIR-II laser irradiation (1064 nm), generates a mild photothermal effect (~43 °C) that rapidly induces plasma membrane rupture and necrotic cell death. This approach offers strong spatiotemporal control and works efficiently at relatively low doses. Importantly, the induced cell damage triggers ICD and activates inflammatory pathways, reshaping the TME and enhancing immune cell infiltration. As a result, both local tumor destruction and systemic anti-tumor immune responses are achieved 253. Chen *et al.* designed and developed a TME-responsive, degradable phototheranostic nanoplatform, CX@PSS, for NIR-II FLI-guided PTT. The system uses a GSH-sensitive polyurethane carrier loaded with a multifunctional NIR-II cyanine PS (CX), allowing it to specifically degrade in the GSH-rich TME, which may help reduce long-term toxicity. Under 808 nm laser irradiation, CX@PSS provides high-resolution NIR-II FLI for precise tumor visualization while simultaneously generating heat for PTT and ROS for PDT. Importantly, this combined phototherapy also induces ICD, which releases tumor-associated antigens and activates anti-tumor immunity. Both cell and animal studies showed strong tumor suppression, good biosafety, and promising potential to reduce tumor metastasis and recurrence 254.

## Design principles of PTT agents for optimal performance

The photothermal effect in plasmonic NPs is primarily governed by factors that determine how efficiently absorbed light is converted into heat. A key parameter is the LSPR, which occurs when the wavelength of incident light matches the natural oscillation frequency of electrons in the NPs, resulting in strong light absorption and efficient heat generation. The efficiency of this process is further influenced by NPs characteristics such as size, shape, composition, and the dielectric properties of the surrounding environment 17, 18. For example, smaller particles often favor absorption-dominated processes, while anisotropic structures such as nanorods can enhance photothermal conversion through stronger plasmonic oscillations. In addition, optical parameters including the absorption cross-section and the ratio of absorption to total extinction determine the level of incident energy is converted into heat rather than scattered 255. For biomedical applications, the excitation wavelength is particularly important. Early photothermal approaches were limited because visible light is strongly absorbed and scattered by biological tissues, restricting treatment mainly to superficial tumors and causing unwanted heating of nearby healthy tissue. This limitation has been largely addressed by using NIR light within the biological windows (≈700–980 nm and ≈1000–1400 nm), where tissue absorption and scattering are lower, allowing deeper light penetration and more efficient activation of NPs within tumors. By localizing heat-generating NPs in tumor tissues and using appropriate laser wavelengths, stronger and more selective heating can be achieved with lower laser power, reducing damage to surrounding healthy tissue 256, 257. Although light penetration in human tissue is still limited to a few centimeters, deeper tumors can be treated by combining NPs delivery with optical fiber or endoscopic illumination, enabling localized photothermal heating at the tumor site.

### Radionuclide theranostics

Currently, PDT and PTT have demonstrated the potential for effective cancer therapy in preclinical studies. Specifically, an external beam is used to irradiate the tumor site to achieve PDT and PTT. A critical challenge that must be overcome in clinical development is the penetration depth. Currently, RIT is a mature technology for the clinical treatment of cancer by self-emitted radiation. Targeted delivery of radionuclides to the tumor remains a limitation of RIT. Radioisotope-related theranostics, such as α- and β-emitters, as well as CR and proton therapy, will be discussed in the following section.

#### Radionanotheranostics of α- and β-emitters

The high linear energy transfer and the short path length of α-emitters enable them to destroy local tumor cells. These properties are suitable for the treatment of small cell clusters and micrometastases. Targeted alpha particle therapy (TAPT) provides more precise tumor cell killing and reduces damage to the surrounding normal tissues. An α-emitting radionuclide, such as ^213^Bi 258, 259 and ^149^Tb 260, is labeled with a monoclonal antibody or targeting ligand to achieve TAPT.

Targeted alpha therapy, a potent modality in nuclear medicine, uses nuclides that emit alpha particles to exert localized therapeutic effects. Nevertheless, radionuclides such as ^223^Ra and ^225^Ac decay into radioactive progeny, which may leave the original vector and cause irradiation in non-target organs. A previous study investigated the fate of ^211^Pb/^211^Bi progeny from ^223^Ra surface-labeled TiO_2_ NPs *in vitro* and in animal model 261. Stability studies demonstrated that up to 40% of ^211^Bi and 25% of ^211^Pb progeny were released within 48 h under static conditions, with biodistribution data confirming these findings *in vivo*. The ^211^Pb activity was still detectable in blood and kidneys, supporting the theory that daughter radionuclides from alpha decay can migrate to non-target organs, thus increasing radiation exposure. Although some studies suggest that progeny remain in target tissues once internalized, this is not always the case, especially considering factors such as lesion size and tumor perfusion. Therefore, it is crucial to analyze the fate of recoils from alpha decay cascades when introducing new radiopharmaceuticals. Further studies are required to quantify progeny release and migration, although preliminary findings support the hypothesis that significant portions of daughter progeny can be transported to nontargeted organs through the bloodstream. Auger emitters, with their high LET that causes clustered DNA damage and efficient cell death, are ideal for targeting metastasized tumors. Despite their potential, few radiopharmaceuticals using Auger emitters exist due to the need for these electrons to reach critical cell components such as the nucleus. One study combined the Auger emitter ^125^I with ultrasmall AuNPs to create a novel radiopharmaceutical 262. These ^125^I-labeled NPs accumulated in the cell nucleus, demonstrating high tumor-killing efficiency in 2D and 3D models. These findings suggest that ultrasmall NPs, which naturally accumulate in the nucleus, could improve targeted radionuclide therapy, especially when combined with tumor targeting agents. This new radiopharmaceutical approach could provide significant advancements in cancer treatment.

Internal α-therapy is effective for micrometastatic diseases, with ^224^Ra being a promising candidate due to its multiple α-particle emissions and a half-life of 3.6 days 263. However, the use of ^224^Ra has been limited to bone-seeking applications because it cannot be stably bound to targeting molecules. A previous study explored calcium carbonate microparticles as carriers for ^224^Ra, aimed at treating cancers in cavitary regions such as peritoneal carcinomatosis. The ^224^Ra-labeled CaCO_3_ microparticles demonstrated high labeling efficiencies and retention of both ^224^Ra and its daughter nuclide ^212^Pb for up to 1 week *in vitro*. Biodistribution studies in mice revealed that the radioactivity predominantly remained in the peritoneal cavity, with reduced skeletal uptake of ^224^Ra at higher microparticle doses. These results suggest that ^224^Ra-labeled CaCO_3_ microparticles could be a promising new method for localized α-therapy in cavitary cancers. PCa, the second most common cancer in men, often progresses to metastatic castration-resistant prostate cancer (mCRPCa), necessitating new targeted therapies.

Hydroxyapatite and TiO_2_ NPs are promising candidates for carrying medicinal radionuclides due to their large surface area, radiation stability, and low toxicity. In one study, they were radiolabeled with ^99m^Tc for diagnostics and ^223^Ra for therapy using two methods, surface labeling and intrinsic incorporation. Both methods achieved >94% labeling yields. Stability tests in various biological media revealed that ^223^Ra-labeled TiO_2_ NPs had the best stability, with <6% activity release over 59 h and <3% over 55 days 264. Both ^99m^Tc-labeled NPs demonstrated approximately 20% activity release in short-term tests. These data suggest that although HAp is better for local applications and controlled release, TiO_2_ is more suitable for systemic applications due to its superior long-term stability. Surface modifications could further improve the stability of these radiolabeled NPs. An injectable brachytherapy hydrogel based on Fe-tannin NPs was developed, allowing stable and chelator-free radiolabeling of clinically used radionuclides (^131^I, ^90^Y, ^177^Lu, ^225^Ac). Crosslinking FeTA NPs with 4-arm PEG-SH created a biocompatible hydrogel capable of prolonged tumor retention and real-time *in vivo* monitoring via MRI signals from embedded ferric ions. In a CT26 colon cancer model, the ^225^Ac-labeled hydrogel effectively eradicated local tumors and, when combined with immunoadjuvant imiquimod and anti-PD-L1 therapy, significantly inhibited metastatic tumor growth by stimulating robust anti-tumor immune responses without adverse effects 265.

**Figure 7:**
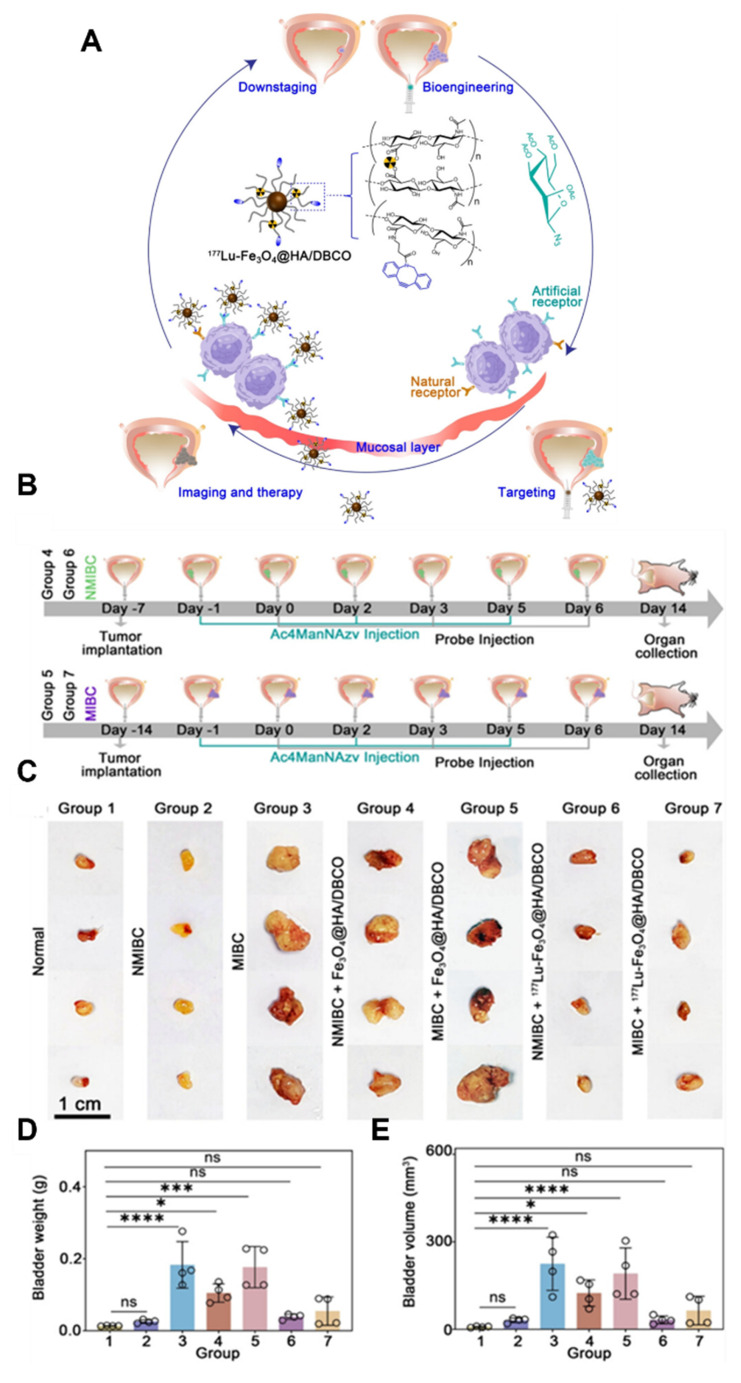
**(A)** Schematic illustration of ^177^Lu-Fe_3_O_4_@HA/DBCO, for MRI and targeted RNT of bladder cancer, **(B)** Treatment schedules for mice bearing NMIBC and MIBC, **(C)** Photographs of bladders from each group, **(D, E)** Bladder weight and volume comparisons among groups. Adapted with permission from 266, Copyright 2024 American Chemical Society.

A multifunctional nanoprobe (^177^Lu-Fe_3_O_4_@HA/DBCO) was developed to enhance bladder-preserving treatment of bladder cancer by integrating mucosal penetration, bioorthogonal targeting, and internal irradiation therapy 266. Iron oxide NPs coated with HA and dibenzocyclooctyne effectively penetrated mucosa and selectively accumulated in azide-expressing cancer cells via bioorthogonal click chemistry (**Figure 7**). Enhanced MRI distinguished non-muscle-invasive from muscle-invasive bladder cancers (MIBC), while internal irradiation by ^177^Lu significantly reduced tumor size.

A multifunctional black phosphorus-based nanosheet (BPNS) system was developed that integrated Cu²⁺ to achieve both rapid degradation and enhanced photothermal stability 267. This BP@Cu structure exhibited superior PTT performance, enabled CDT by generating •OH, and facilitated real-time PET imaging using ^64^Cu²⁺. The approach demonstrated effective tumor-targeting capability and introduced a versatile nanoplatform for PET-guided, CDT-enhanced photothermal cancer therapy. A polymersome-based nanocarrier was developed to modulate innate immunity by efficiently targeting splenic red pulp myeloid cells, significantly reducing tumor growth in a mice melanoma model 268. Utilizing ^89^Zr-radiolabeling and PET imaging, large spherical polymersomes rapidly accumulated in the spleen, effectively delivering β-glucan to hematopoietic organs. Preclinical biodistribution studies in non-human primates confirmed splenic targeting and biocompatibility, demonstrating their translational potential for cancer immunotherapy. GSH-modified GNCs labelled with radionuclides (^99m^Tc@Au NCs and ^177^Lu@Au NCs) were synthesized via a simple chelation strategy, significantly enhancing internal radionuclide therapy (RNT) efficacy. While ^99m^Tc@Au NCs enabled both imaging and radiosensitization, the therapeutic radionuclide ^177^Lu@Au NCs effectively induced ICD, activating DC and synergizing with anti-PD-L1 checkpoint blockade to suppress distant and spontaneously metastatic tumors 269. Radiolabeled, urease-powered mesoporous silica nanobots were evaluated in an orthotopic mice model of bladder cancer, demonstrating significantly enhanced tumor accumulation and penetration. Using PET imaging with ^131^I, nanobots achieved approximately eightfold higher tumor localization, validated by *ex vivo* optical imaging methods 270. This increased accumulation enabled effective radionuclide therapy, reducing tumor size by roughly 90% at notably lower therapeutic doses.

Wang *et al.* developed a radionuclide-assisted chemodynamic therapy platform, ¹²⁵I-MIL-88B(Fe), to improve cancer treatment in pancreatic tumors. The system consists of iron-containing metal–organic framework NPs that can concentrate hydrogen peroxide and promote the Fenton reaction to generate highly toxic •OH. The attached radioiodine-125 continuously produces hydrated electrons in the tumor environment, which help convert Fe³⁺ to Fe²⁺. By accelerating this iron redox cycling, the NPs produce more ROS and achieve stronger tumor cell killing than conventional CDT alone. Both cell and animal studies confirmed enhanced anti-tumor effects, mainly through ROS-triggered activation of the MAPK/p53 apoptosis pathway 271.

A new radioembolic agent, ¹³¹I-labeled methacrylated gelatin microspheres (¹³¹I-GMs) were developed for transcatheter arterial radioembolization (TARE) in HCC. These particles are biodegradable, elastic, and highly uniform in size, which is important for safe and effective arterial delivery. They were synthesized using a microfluidic method that allows precise size control, making them potentially useful for personalized radionuclide therapy. In animal studies, the Ms showed good radioactive stability, remained well retained in the hepatic artery and tumor tissue, and significantly slowed liver tumor progression 272. Jia *et al.* developed a multifunctional NPs system, ¹³¹I-BaGdF₅@PDA-CDDP, to combine transarterial chemoembolization (TACE) and TARE for improved treatment of HCC. The NPs carry cisplatin for CMT and iodine-131 for radionuclide therapy, allowing simultaneous local PTT and CMT directly inside the tumor after injection through the hepatic artery. This is important because RNT can help kill residual tumor cells that often survive after embolization and contribute to recurrence or metastasis. The BaGdF₅ core also provides CT/MRI capability, while ¹³¹I enable SPECT imaging, allowing real-time monitoring of NP distribution and treatment delivery *in vivo*. In animal models, this combined approach reduced tumor metabolism and increased tumor cell apoptosis more effectively than single treatments alone 273.

Shao *et al.* developed ¹⁷⁷Lu-labeled porous granular hydrogels as a new RNT platform for liver tumors. These injectable particles combine embolization and local internal radiation, while also allowing SPECT imaging for treatment tracking. Compared with conventional radioactive Ms, they showed stable radionuclide loading, controllable size, good tumor retention, and reduced reflux into non-target tissues. In animal models, they achieved strong tumor inhibition with good safety, highlighting their promise for precise intra-arterial brachytherapy 274. Xiao *et al.* developed ¹⁷⁷Lu-labeled polymeric Ms for RNT of liver cancer through image-guided intra-arterial brachytherapy. ^177^Lu provides both local radiation treatment and SPECT imaging, allowing real-time tracking after delivery. The Ms showed stable radionuclide loading, uniform tumor embolization, strong anti-tumor effects, and good safety without off-target embolization 275.

Li *et al.* developed a multifunctional biodegradable microsphere (¹³¹I-ICT/R848-MS) for HCC that combines transarterial radioembolization with CMT and immune activation. The RNT component, iodine-131, is important because it delivers local radiation to kill tumor cells and induce ICD, while also enabling SPECT/CT imaging to monitor microsphere retention and distribution after treatment. The Ms also carry icaritin, which further promotes tumor cell death, and R848, an immune stimulator that enhances dendritic cell activation and strengthens T-cell–mediated anti-tumor immunity. In tumor models, the Ms showed strong tumor-specific retention, robust immune activation, and improved anti-tumor effects without relapse 276. This study by Zhao *et al.* highlights a promising RNT strategy using ¹⁷⁷Lu-LNC1004, a fibroblast activation protein (FAP)-targeted radiopharmaceutical designed to deliver radiation directly to tumors and remain there for longer periods. In preclinical models, ¹⁷⁷Lu-LNC1004 showed strong tumor uptake and anti-tumor activity, however it also temporarily increased PD-L1 expression, suggesting that tumors may activate immune escape pathways after radiation. To address this, it was combined with anti-PD-L1 immunotherapy, which resulted in complete tumor eradication and long-lasting immune memory in mice. Mechanistic analyses showed that this combination reshaped the TME by enhancing CD8⁺ T-cell activity, increasing M1 macrophages, improving immune communication, and broadening T-cell receptor diversity. Early clinical data also suggested that ¹⁷⁷Lu-LNC1004 is safe, well-tolerated, and potentially effective in patients with advanced FAP-positive cancers 277.

Zhang *et al.* developed a novel RNT strategy using a ²²³Ra/Ba single-atom nanozyme to induce tumor cell senescence and enhance anticancer immunity. The radionuclide ^223^Ra was delivered efficiently using a barium-based nanozyme carrier, which also has enzyme-like catalytic activity that increases ROS production, leading to tumor cell damage and senescence. However, senescent cells can promote tumor recurrence, the treatment was combined with anti-PD-L1 immunotherapy inducing senescence with radionuclide therapy, then eliminating those cells through immune activation. This combination significantly suppressed both primary and distant tumors while improving immune response 278. Pei *et al.* inactivated *Salmonella* and labeled it with iodine-131 (¹³¹I-VNP) as a tumor-targeting carrier for RNT. The bacteria helped retain radioiodine in tumors, improve internal radiation delivery, and activate both innate and adaptive immunity. Importantly, treatment stimulated the innate immune pathway, promoted dendritic cell maturation, and enhanced T-cell responses. When combined with anti-PD-L1 therapy, it not only controlled primary tumors but also suppressed distant tumors and helped prevent recurrence, showing strong potential for radio-immunotherapy 279.

ATP-responsive nanoassembly, USINAs (¹³¹I-aPD-L1), was developed by Shen *et al.* to improve RNT by combining ferroptosis, radiopharmaceutical therapy, and immunotherapy in one platform. This system exhibited good stability in blood and then selectively disintegrated in the ATP-rich TME, releasing ultrasmall iron NPs and ¹³¹I-labeled anti-PD-L1. The iron NPs induce ferroptosis, while the ¹³¹I component delivers targeted radiation and also blocks PD-L1, creating a dual radioimmunotherapy effect. Importantly, RNT and ferroptosis reinforced each other: radiation increased ROS and sensitized tumors to ferroptosis, while ferroptosis made tumor cells more vulnerable to radiation. This combination also triggered strong ICD, promoted dendritic cell maturation, enhanced T-cell responses, and supported SPECT imaging for treatment monitoring 280.

#### Radionanotheranostics of Cerenkov radiation (CR)

Currently, PET, SPECT, and CT are general translational imaging systems to perform radio nanotheranostics in clinical settings. The high-energy radionuclides release three signals, viz., gamma energy for imaging systems and redshifted emission and blue light for optical imaging and PDT 281. One of the signals, blue light, constitutes a novel avenue for basic and applied science by combining advances in nanotechnology and materials science.

CR, also termed Cherenkov luminescence light (CLI), is an optical light originating from energetic radionuclides, especially β-emitters. In general, CR can be used to initiate photodegradation of compounds and generate fluorescent emission for imaging 282. CR signals are tissue-depth-dependent and entirely restricted by background light. To improve the intensity of CR signals, radiopharmaceutical-excited FLI 283, secondary Cherenkov-induced FLI 284, Cerenkov radiance energy transfer (CRET) 285, radioluminescence imaging 286, and radinuclide energy transfer 287 have been validated. A novel therapeutic approach was developed to improve the effectiveness of CR-PDT and minimize its side effects using ^131^I-labeled ALA-loaded EMs (^131^I-EM@ALA) 288. This multifunctional platform was designed to target tumors through the EPR effect and homologous targeting, where the abundant mitochondria would convert ALA into the active PS PpIX, generating ROS under CR stimulation. The combination of ^131^I RT and CR-PDT was anticipated to exert strong anti-tumor effects with minimal adverse effects due to the low accumulation of PpIX in normal tissues. Results showed that a single injection of ^131^I-EM@ALA significantly inhibited tumor growth in subcutaneous tumor-bearing mice, and the strategy effectively reduced treatment side effects compared with that using PpIX directly. This study presents a promising multitherapeutic strategy for anti-tumor treatment, combining RT and CR-PDT without the need for an external light source.

**Figure 8:**
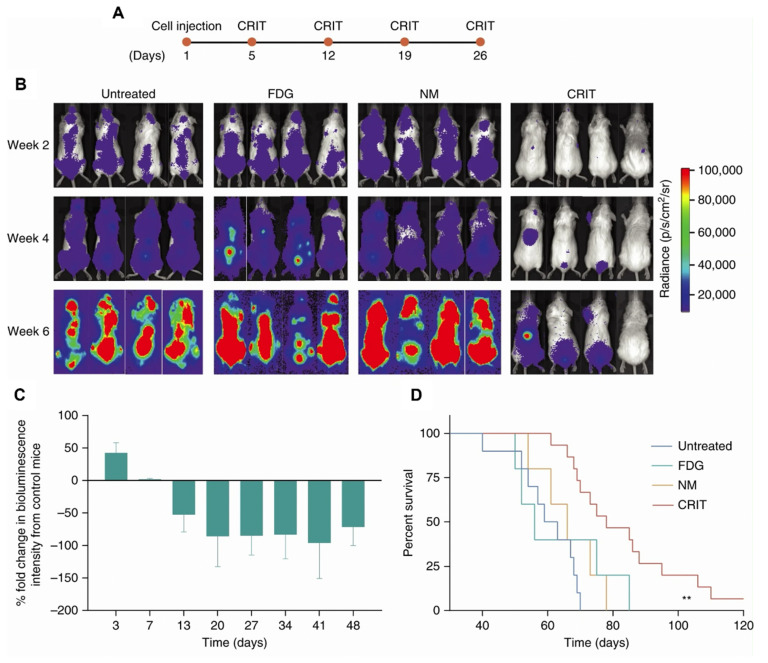
Response of multiple myeloma to CRIT. **(A)** Treatment timeline, **(B)** BLI of representative multiple myeloma-bearing mice under various treatments, **(C)** changes in bioluminescence intensity following treatments relative to untreated control, **(D)** survival comparison showing twofold increase in survival for treated mice versus controls. Adapted with permission from 291, Copyright 2018 Springer Nature.

The therapeutic efficacy of traditional PDT is often limited by the shallow penetration of external light. To address this problem, CR has been investigated as an alternative; however, existing type I PSs for CR-induced PDT (CRIT) have significant limitations. An innovative approach by engineering NH_2_-Ti_32_O_16_ nanocluster-derived ultrasmall nano-PSs (TDPs) using dopamine ligands was designed and developed by Li *et al.* 289. These ligands improved water solubility, photocatalytic properties, and tumor targeting through dopamine receptor binding on cancer cells. Under CR irradiation, TDPs effectively generate •OH by separating electron–hole pairs, resulting in improved type I PDT and augmented CDT. The use of CR not only promotes CRIT but also improves CDT, demonstrating significant *in vitro* and *in vivo* anti-tumor properties with minimal side effects. Another study by Guo *et al.* integrates nanotechnology with nuclear medicine, thereby advancing CR-induced combined therapy and nanocatalytic medicine. The combination of Ce6@GEV and ^18^F-FDG significantly reduced cell viability from 88.02% to 23.79%, demonstrating effective CL-induced PDT 290. The Ce6@GEV + ^18^F-FDG treatment group achieved the highest tumor inhibition rate of 58.02% and the longest survival rate of 35 days (40%) compared with other groups. This approach, by combining Ce6 and ^18^F-FDG, aims to address the limitations of traditional PDT and may extend its application to various tumors. In addition, NPs were combined with CR to improve diagnosis and theranostics. CR-induced PDT can kill cancer cells, and the therapeutic types can be divided into the following three approaches: 1) free radionuclides combined with drug-loaded NPs, 2) radionuclide-modified NP-doped PS, and 3) radionuclides combined with NPs. First, CR generated from ^18^F radionuclides activate tumor-surrounding photosensitive drugs to induce cell death 291 (**Figure 8**). Moreover, chemotherapeutics transforms to radiotherapeutics for the precise treatment of disseminated cancer. The strategy rescues abandoned photosensitive drugs with poor therapeutic outcomes into precision phototherapeutics.

Goel *et al.* developed a multifunctional core–satellite nanostructure by combining copper sulfide (CuS) NPs with [^89^Zr]-labeled hollow mesoporous silica nanoshells filled with porphyrin molecules to improve cancer imaging and therapy 292. It enables simultaneous tetra-modal imaging (PET, fluorescence, Cherenkov luminescence, and CR energy transfer) for accurate tumor detection and multimodal image-guided therapy. The synergy between CuS-mediated PTT and porphyrin-mediated PDT resulted in complete tumor eradication within a day, with no recurrence. The study emphasizes a versatile approach to creating high-performance core–satellite nanohybrids, which can be tailored for various imaging and therapeutic applications. Cerenkov imaging is advancing the use of radiotracers and therapeutic agents in nuclear imaging by shifting Cherenkov light to the red-light region using europium oxide NPs. Traditional CR is limited by its UV/blue emission, which is poorly penetrative *in vivo*. To address this problem, Zhang *et al.* developed ultrasmall Eu_2_O_3_ NPs with improved brightness through a down-conversion technique involving europium oxide and trimethylamine N-oxide 293. These NPs, functionalized with polyethylene glycol and radiolabeled for intravenous injection, enabled more effective cancer imaging in mice, including lymph node and tumor visualization. The high density of emitters and efficient fluorescence of europium make it ideal for improving Cherenkov light and achieving sensitive, multimodal imaging. This approach not only improves the *in vivo* imaging capabilities of europium-based NPs but also lays the groundwork for further development of smaller, biocompatible particles for potential clinical applications and advanced diagnostic technologies.

Radionuclides have distinct patterns of emission for imaging and RT. Some CRIT involves radiation emission from α- and β-emitters, as well as a contrast agent for SPECT or PET imaging, and can serve as radiotheranostics for the precise treatment of cancer. Furthermore, the low range and high energy of α-emitters can treat lung tumor colonies. β-emitters can treat a wide range of cancers, such as hepatocarcinoma and ovarian and head and neck cancers. CR is an optical light generated from energetic radionuclides and restricted by tissue depth. The participation of NPs can improve CR application from diagnosis to theranostics. In addition to treating solid tumors, CR-combined NPs have the potential to treat disseminated cancers.

Su *et al.* introduced a strategy using CR from the clinical radiotracer [⁶⁸Ga]Ga-FAPI as an internal light source to activate TiO₂ NPs for CR-mediated PDT (CR-PDT). Since [⁶⁸Ga]Ga-FAPI specifically targets CAFs, it enables localized CR generation within tumors. The activated NPs produce ROS that damage both tumor cells and CAFs, reduce collagen formation, and affecting the ECM structure. This remodeling relieves vessel compression, improves drug penetration, and enhances CMT delivery. Additionally, CR-PDT consumes oxygen, increasing tumor hypoxia, which further activates hypoxia-sensitive drugs for greater therapeutic effect 294. Zhu *et al.* developed a biohybrid system made of the probiotic bacterium *E. coli* Nissle 1917 loaded with an aggregation-induced emission PS. After administration, these biohybrids preferentially accumulate in tumors with the tumor-seeking radiopharmaceutical ¹⁸F-FDG, whose CR activates the PS directly inside the tumor. This internal activation triggers CR-PDT, leading to tumor cell damage and ICD. Importantly, the bacteria also act as immune stimulators, improving DC maturation and CD8⁺ T-cell activation. As a result, the combination of CR-PDT and immune activation produced much stronger tumor inhibition and longer survival than CR-PDT alone, while maintaining good safety 295.

A tumor-selective CR-PDT system (⁸⁹Zr-ALA-Liposome-ART) that uses ⁸⁹Zr as an internal CR source to activate protoporphyrin IX (PpIX) inside tumor cells was developed by Liu *et al.* The acidic tumor environment triggers ALA release, while the conversion of PpIX to heme is further used to activate artemisinin. This design improves the selectivity and effectiveness of CR-based therapy, leading to strong tumor inhibition and enhanced anti-tumor immunity. When combined with anti-PD-L1, it also helped suppress tumor recurrence 296. Jo *et al.* developed a precise Cerenkov luminescence energy transfer-based PDT for HER2-positive cancers by combining two clinically used components: 5-aminolevulinic acid (5-ALA) and ⁶⁴Cu-DOTA-trastuzumab. After administration, 5-ALA is selectively converted inside cancer cells into the PS protoporphyrin IX (PpIX), while ⁶⁴Cu-DOTA-trastuzumab specifically accumulates in HER2-expressing tumors. When both localize in the same tumor, Cerenkov light emitted by ⁶⁴Cu excites PpIX directly inside the cancer, producing a highly tumor-selective PDT effect 297. Rosenkrans *et al.* developed semiconducting polymer NPs (SPNs) to improve CR-induced therapy (CRIT). Since CR is a weak but internally generated light source, the SPNs were designed to efficiently capture and amplify it, then transfer the energy to a PS for stronger PDT effects. The NPs also showed good tumor uptake on PET and optical imaging, supporting both therapy and imaging. 298. Herein, we list α-emitters, β-emitters, and CR to compare their characteristics (**Table 3**).

### Comparison of proton therapy and intensity-modulated radiation therapy (IMRT)

RT is commonly applied to cancerous tumors due to its ability to control cell growth. Radionuclides that emit gamma rays or positrons can provide diagnostic information and identify the functions of specific organs and are also used for tumor therapy by systemic or targeted circulation. Compared with radionuclide-associated nanotheranostics, proton therapy is another method of external beam RT that utilizes ionizing radiation in clinical settings 299, 300. Proton therapy represents a type of radiation that uses a particle, the proton, to deliver radiation while minimizing the dose to nearby organs. Protons have mass and a positive elementary charge, and with high momentum to a specific depth that depends on both the initial speed of the protons and the density of tissue. At that specific depth, protons rapidly decelerate, and this rapid deceleration deposits the dose with a steep falloff phenomenon, known as the Bragg peak 301. These charged particles damage the DNA of cells, ultimately killing the cells by blocking reproduction. Cancerous cells are particularly vulnerable to attacks on DNA because of their high rate of division and reduced ability to repair DNA damage. The physical processes produced by protons that generate positron emitters can be monitored during or after irradiation by PET. Furthermore, CR is used to measure the nuclear cross-sections of protons, and research suggests that the method is convenient and widely applicable for high-precision proton therapy 302. To improve the effect of proton therapy, metallic NPs could be used to augment efficacy through the particle-induced X-ray emission effect. The metallic NPs generate secondary electrons and characteristic X-ray to cause tumor cell death after proton irradiation. In addition to therapeutic improvement, secondary radiation also exhibited high contrast in a multispectral imaging system 303. In clinical settings, the latest generation of proton treatment methods is known as intensity-modulated proton therapy (IMPT), which adjusts the precision, depth, and intensity of a proton beam to complex spider-like tumors, simultaneously avoiding healthy tissue. Compared with RT, IMPT significantly reduces the dose in surrounding normal tissues 304. Although proton therapy is a good method to treat patients with cancers, there are three major challenges, viz., 1) its limited availability, which may delay or preclude treatment for patients who require expedited treatment, 2) the high cost of proton therapy, i.e., some insurance companies may not approve payment of this treatment, and 3) inapplicability to patients with disseminated tumors. Because of these challenges, other methods of radiation therapy are more widely utilized.

Proton therapy combined with bismuth NPs (Bi NPs) was evaluated for enhanced treatment of deeply-seated, unresectable tumors 305. Bi NPs synthesized via pulsed laser ablation in liquids and coated with Pluronic-F127 polymer showed good colloidal stability and moderate toxicity below 25 μg/mL. At the Bragg peak, proton irradiation significantly increased apoptosis, disrupted cell membranes, reduced clonogenic activity up to 97%, and notably suppressed primary tumor growth by 60% and metastatic spread in S37 sarcoma-bearing mice, demonstrating substantial promise for improved cancer RT. Proton-based RT combined with noble metal NPs was evaluated to determine factors influencing ROS generation. Ligand-free colloidal platinum (Pt) and gold (Au) NPs, synthesized without organic stabilizers to avoid interference, were irradiated, generated ROS, which depend mainly on total particle surface area rather than mass or size 306. Pt NPs consistently exhibited higher ROS yields compared to Au and Au-Pt alloy NPs, emphasizing the significant role of surface atom chemistry in ROS generation. These findings clarified that surface characteristics of noble metal NPs substantially influence proton-induced ROS production.

**Figure 9:**
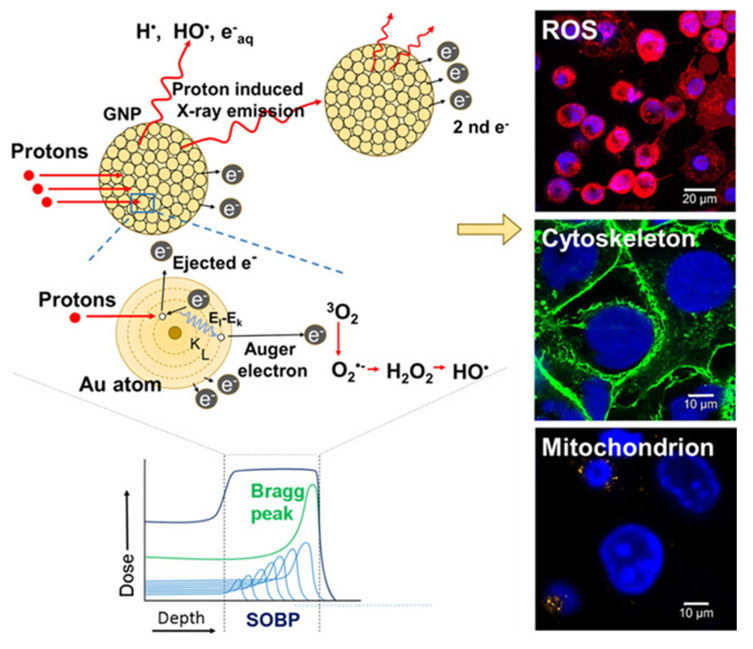
Mechanism of ROS production by incident protons interacting with Au atoms in a GNP, releasing primary electrons, X-rays, and Auger electrons, which may react with surrounding water molecules to generate ROS. Adapted with permission from 308, Copyright 2023 American Chemical Society.

Simulations evaluating GNPs as RSs in proton therapy revealed that ROS generated upon irradiation diffused several hundred nanometres away, maximizing enhancement around 50 nm from the NPs, particularly towards the proton source 307. Although proximity between NPs significantly elevated local doses, it also reduced overall reactive species yield by up to 60% due to increased absorption. These findings emphasized balancing dispersed NP distributions to enhance total radiolysis yield with clustered arrangements strategically positioned near biological targets, guiding the optimal use of GNPs in proton therapy (**Figure 9**). A recent study evaluated GNPs as RSs in proton beam therapy (PBT) by investigating their ability to enhance ROS generation in tumor cells irradiated with a 230 MeV proton beam at the spread-out Bragg peak. Proton irradiation triggered electron emission from GNPs, leading to increased ROS production and severe mitochondrial and cytoskeletal damage within 48 h post-irradiation, resulting in a radiosensitization enhancement factor of 1.24 at 30% cell survival after 8 days 308.

Lee *et al.* developed a three-dimensional (3D) spheroid-based high-throughput screening platform to evaluate how head and neck cancer cells respond to PBT and drug combinations. Using two cancer cell lines, the system allowed precise delivery of different proton doses and testing of multiple drugs at the same time. The results showed that both proton radiation and certain drugs reduced tumor cell viability in a dose-dependent manner. Importantly, combination therapy with olaparib showed a strong synergistic effect 309. Tudor *et al.* showed that iron oxide NPs loaded with doxorubicin can sensitize chondrosarcoma cells to proton therapy, and can overcome chemo- and radioresistance. The combination reduced cell survival, increased DNA damage, and was more effective with low-LET protons than with high-LET protons. It also caused measurable changes in cell and nuclear features, suggesting possible markers for treatment response 310. Silva *et al.* showed that porphyrin-coated Au NPs can improve treatment of triple-negative breast cancer by combining PDT with RT. After light activation, they increased ROS and ^1^O_2_, disrupted mitochondrial function, inhibited thioredoxin reductase, and enhanced cancer cell killing. The combination reduced tumor cell survival more effectively than radiation alone, with proton therapy showing the strongest effect 311.

IMRT is an advanced mode of high-precision photon RT that uses computer-controlled linear accelerators to safely and painlessly deliver precise radiation doses to a malignant tumor or specific areas within the tumor. IMRT allows for the radiation dose to conform more precisely to the three-dimensional shape of the tumor by modulating or controlling the intensity of the radiation beam in multiple small volumes. IMRT delivers the same photons to treat a tumor but with the potential to lower the high doses of radiation received by healthy structures. Currently, IMRT is being employed most extensively to treat PCa, the head and neck, and the central nervous system. IMRT has also been used in limited situations to treat breast, thyroid, lung, as well as in gastrointestinal, gynecologic malignancies, and certain types of sarcomas. IMRT may also be beneficial for treating pediatric malignancies 312. The radiotherapeutic management of cancers is rapidly changing, with IMRT now being the standard of care. Moreover, AuNPs are used to increase the therapeutic effect of IMRT and improve X-ray imaging in brain and PCa 312, 313. Metallic NPs are agents that can potentially improve diagnostic imaging and IMRT and selectively improve RT effectiveness. In clinics, the challenges of IMRT include disseminated tumor treatment and that a larger range of normal organs receive a low dose of radiation.

A clinical trial evaluated NBTXR3 (Hafnium oxide NPs) in combination with external beam radiotherapy in patients with advanced soft tissue sarcoma prior to surgical intervention. Of twenty-two patients who finished therapy were monitored for a duration of up to 40 months. Tumors diminished by approximately 40% at elevated dosages. Results indicated that NBTXR3 was safe, effective in diminishing tumor size, and potentially suitable for preoperative use in advanced sarcoma patients. Preoperative chemo-RT, a standard treatment for locally advanced rectal cancer, was administered in conjunction with PEP503 (NBTXR3), which has demonstrated the ability to maintain therapeutic efficacy at diminished radiation doses 315. A prospective, single-arm Phase Ib/II clinical trial evaluated the safety and efficacy of PEP503 administered via intratumoral injections in conjunction with concurrent chemoradiotherapy in patients without metastatic disease. In Phase Ib, dose escalation established the appropriate dosing, whereas Phase II assessed anti-tumor response rates. This groundbreaking study confirmed PEP503's ability to safely enhance the effectiveness of radiation in advanced rectal cancer treatment. The Act.In.Sarc trial tested NBTXR3, combined with RT in patients with locally advanced sarcoma 316. Results showed the formulation significantly improved complete tumor removal and did not increase serious side effects compared to radiation alone. Treated patients had fewer serious adverse events and reported better quality of life scores. NBTXR3 was tested for the first time in a patient with non-operable PDAC. The NPs were safely injected into the tumor using an endoscope, followed by RT (45 Gy delivered in 15 fractions) 317. The treatment caused no immediate side effects, and imaging showed effective local tumor targeting. These initial results indicated that endoscopic delivery of NBTXR3 with radiation was safe and feasible, offering a potential new approach for treating patients who cannot undergo surgery. A phase I study tested the safety of the radioenhancer NBTXR3 in patients with advanced tumors, given by injection at increasing doses followed by RT. NBTXR3 was safe, causing no serious side effects, and patients tolerated it well, even at higher doses. The study did not reach a maximum tolerated dose, and early signs suggested that NBTXR3 could effectively slow tumor growth. These results supported further testing of NBTXR3 at a recommended dose combined with RT 318.

Chajon *et al.* evaluated NBTXR3, a radioenhancer injected directly into liver tumors, in combination with stereotactic body RT for HCC and liver metastases. NBTXR3 was designed to improve the effect of radiation specifically within the tumor. In this trial, the treatment showed a manageable safety profile, with no dose-limiting toxicities and no treatment-related deaths. Most serious side effects were not directly linked to NBTXR3, and the recommended dose was established at 42% of the gross tumor volume. Early results also showed encouraging anti-tumor activity, with objective responses seen in about half of treated lesions in both liver cancer and metastatic cases 319. We summarize proton therapy and IMRT with NPs for theranostics in **Table 4**. Some NPs participate in proton therapy and IMRT to improve localized therapeutic efficacy in preclinical studies.

### Advancing cancer immunotherapy with NPs

NP based phototherapies, including PDT and PTT, provide several advantages, such as minimal invasion, high efficacy, and minimal side effects, compared with traditional anti-tumor therapies. PDT and PTT ablate tumors through ROS and heat. Moreover, the consequent tumor cell necrosis or apoptosis after PDT or PTT provides a potential antigen pool to induce an immune response 320, 321. Compared with other anti-tumor therapies, such as PDT, PTT, and CMT, the target in cancer immunotherapy is not tumor cells directly but rather the body’s immune system. The tactics of cancer immunotherapy include triggering the natural capacity of the immune system to attack tumor cells. Studies in cancer immunology and cancer immunotherapy, including artificial antigen-presenting cell-based therapy 322, and cell-based therapies, such as DC, adoptive T-cell transfer, and checkpoint blockade therapy 323-325, have suggested promising clinical applications in the past decade, especially for checkpoint blockade therapy. For instance, in 2018, a few Nobel laureates established a whole new strategy for cancer therapy. James P. Allison investigated a known protein, termed cytotoxic T-lymphocyte-associated antigen 4 (CTLA-4) 326, and Tasuku Honjo found a protein, called PD-1 327, on immune cells. Both these proteins operate as a brake on the immune system, but with a different mechanism. They demonstrated a different operation by releasing these brakes on the immune system, which could trigger immune cells to attack tumors 328. Such improvement has provided various combined treatments, such as PTT and immunotherapy. PTT not only ablates the tumor by hyperthermia-induced cell apoptosis and necrosis but also triggers immunotherapy to treat both primary and metastatic tumors by killing tumor cells, releasing TAA and then inducing a systemic anti-tumor immune response. In fact, this method is already being used in preclinical and clinical studies 329.

#### NPs combined with ICB

Ionizing RT is a functional tool for local tumor treatment, but it is restricted to symptom mitigation. Recently, a crucial approach to improve cancer treatment has been to combine RT with checkpoint blockade to induce not only a local tumor treatment effect but also systemic regression of metastatic disease, known as the abscopal effect 330. Further research has demonstrated that when anti-CTLA-4 antibody is combined with RT for tumor treatment, fractionated, rather than a single-dose, RT induces higher efficacy of the abscopal effect 331. For successful checkpoint blockade therapy against cancer, it is necessary to reduce immune-related cytotoxicity. Targeted delivery of checkpoint inhibitors to specific locations is also critical. Numerous NPs have been investigated to provide controlled release to the tumor site. Several preclinical studies have shown that NP-based targeted delivery of anti-CTLA-4 antibody augments tumor destruction efficacy and reduces cytotoxicity. Nevertheless, there are still no clinical studies to estimate the NP-mediated delivery of checkpoint inhibitors combined with RT. NP-mediated ICB treatment combined with RT may provide another opportunity to achieve optimal anti-tumor efficacy with specific targeting and decreased cytotoxicity 332.

In traditional cancer CMT, the most crucial problem is tumor metastasis due to its significant effects and restricted efficacy 333. A probable option is to trigger the immune system to treat metastatic tumors. NPs combined with immunoadjuvants not only induce an innate immune response but also increase immune cell infiltration in the TME to release inflammatory cytokines, such as IL-6, IL-12, IL-1β, TNF-α, and IFN-γ 334. PTT-induced hyperthermia can inhibit tumor growth and metastasis by upregulating the expression of HSPs 335. NPs-based PTT can achieve a higher concentration of particle accumulation in the tumor site because of active or passive targeting and kill tumors via a thermal effect. Cell debris and TAA are released and further activate the immune system to kill metastatic tumors. Nevertheless, a major challenge exists not only in PTT but also in immunotherapy. Specifically, tumor cells are tolerant to the immune system under certain conditions. Some anti-tumor negative immune regulatory signals, such as CD47 signal regulatory protein-α (SIRPα) on macrophages 336, CTLA-4 on regulatory T cells 337, and PD-1 on T cells 338, block the immunotherapy of immune cells, which causes treatment failure. Therefore, PTT combined with immunotherapy can improve treatment efficacy. For instance, Chen *et al.* developed a combined adjuvant NPs-mediated PTT with checkpoint blockade immunotherapy. ICG, a photothermal agent, and a toll-like receptor seven agonist (R837), were loaded in PLGA, which, when combined with anti-CTLA-4 exerted a markedly suppressive effect not only on primary tumors but also on distant tumors. Furthermore, this combined treatment resulted in long-term survival by inducing the memory of T cells, thus inhibiting tumor recurrence. Anti-PD-L1 combined with AuNS-mediated PTT was found to be an effective treatment for primary tumors and distant tumors via heat ablation-induced immune response in a bladder cancer animal model 339. In recent years, a large number of clinical trials have demonstrated that combined treatment with anti-CTLA-4 and anti-PD-1 can achieve better treatment efficacy than monotherapy of anti-CTLA-4 or anti-PD-1 in sarcoma and melanoma 340, 341.

PDT that kills cancer cells using PSs to generate ROS under irradiation is a valid therapeutic modality that provides minimal invasiveness 6. PDT can induce anti-tumor immune responses by producing tumor-derived protein antigens from dying tumor cells. Nevertheless, traditional PSs used in PDT have limited tissue penetration depth because of the short excitation wavelength. Although PDT may induce immune responses, such effects are generally not sufficiently powerful for inhibiting tumor growth after PDT 342. PDT causes Treg cell infiltration, which causes overexpression of PD-L1 to inhibit tumor-killing by immune cells, such as T lymphocytes 343. Therefore, combination with ICB therapy to improve the anti-tumor immune response of PDT constitutes a better strategy.

A multifunctional NP system (PIC), composed of polylysine, iron oxide, and CpG, was designed to enhance the in-situ vaccine effect of radiation therapy (RT) and improve tumor response to ICB. RT alone has limited efficacy in optimizing tumor antigen presentation and modulating the tumor immune microenvironment, but the addition of PIC increases antigen presentation, shifts TAMs towards an M1-dominant phenotype, and stimulates a type I interferon response. In immunologically “cold” murine tumor models, the combination of RT, PIC, and ICB significantly improves tumor control, extends survival, and induces tumor-specific immune memory. This approach offers a scalable and readily translatable strategy to transform tumors into sites of adaptive immune activation, enhancing responsiveness to ICB and potentially other immunotherapies. Given the broad applicability of RT and PIC across solid tumor types, further preclinical and clinical investigations are warranted to evaluate their potential in metastatic cancer treatment 344. A nanomaterial-based approach to enhance PDT and anti-tumor immunity using a 3-bromopyruvate (BrP)-anchored nanoscale metal–organic layer (BrP@MOL) 345. By inhibiting mitochondrial respiration and glycolysis, BrP@MOL reprograms tumor metabolism, increasing oxygen availability and reducing lactate production, thereby alleviating hypoxia and immunosuppression in the TME. This metabolic modulation enhances ROS generation during PDT, leading to over 90% tumor growth inhibition, with 40% of treated mice achieving complete tumor regression and resistance to re-challenge, as well as prevention of lung metastasis. When combined with αPD-L1, the therapeutic efficacy further improves, resulting in >98% tumor inhibition and complete tumor clearance in 80% of mice.

A novel therapeutic approach for oral squamous cell carcinoma (OSCC) was developed by integrating NIR-II PTT with systemic immune activation. Using polymer-based NPs with strong electron donor-acceptor structures, this strategy enhances photothermal conversion, stability, and biocompatibility, enabling deep tumor ablation with minimal off-target effects. RNA sequencing reveals that PTT-treated cancer cells upregulate apoptosis-related and antigen-presenting pathways, leading to ICD, increased TAA release, and activation of DAMPs. This, in turn, stimulates dendritic cell activation and an adaptive immune response, effectively targeting both primary tumors and lymph node metastases. Compared to conventional ICG dyes, PNPs demonstrate superior photostability and photothermal efficiency, further amplifying the immunogenic response. The study underscores the potential of NIR-II photothermal immunotherapy as a paradigm shift in OSCC treatment, offering a promising strategy for tumor eradication and metastasis prevention. 346.

#### NPs enabled ICD

ICD is a regulated form of cell death that has the potential to elicit antigen-specific immune response. There are several determinants for ICD to occur that includes cellular stress, cell death, antigenicity, and adjuvanticity. Briefly, unregulated, accidental cell death cannot be immunogenic, dying cancer cells must express antigens to be recognized and taken up antigen-presenting cells of adaptive immune response such as DC or macrophages. To boost the adaptive immune response, ICD requires robust adjuvants such as HMGB1, extracellular ATP and translocation of calreticulin (CRT) from ER to plasma membrane. NP-based ROS-inducing therapies are well known to induce ICD through oxidative stress or proteotoxic stress and are summarized in this section 347.

Triple negative breast cancer** (**TNBC) often resists immunotherapy due to its immunologically unresponsive microenvironment. Wang *et al.* developed a gas nanoadjuvant using a virus-mimicking hollow mesoporous silica doped with tetrasulfide, which co-encapsulates AIE-active luminogen and manganese carbonyl for the immunotherapy of breast tumors 348. This nanosystem releases hydrogen sulfide and carbon monoxide in response to tumor-specific conditions, enhancing PDT and activating an innate immune pathway. Upon NIR laser irradiation, the AIEgen-mediated therapy causes mitochondrial damage and mitochondrial DNA leakage, which, along with the released CO and Mn^2+^, activates an innate immune pathway. This strategy effectively improves the photoimmunotherapy of poorly immunogenic TNBC, resulting in significant tumor inhibition, and metastasis elimination in female mice. In this study, Turubanova *et al.* investigated two novel porphyrazine-based PSs, pz I and pz III, for their ability to induce ICD 349. Both PSs, at an optimal light dose of 20 J/cm², effectively induced cell death in cancer cells, with pz I localizing in the Golgi apparatus and lysosomes and pz III in the ER and lysosomes. Cell death induced by pz I-PDT was inhibited by an apoptosis inhibitor but not by ferroptosis or necroptosis inhibitors, while pz III-PDT-induced cell death was affected by both apoptosis and necroptosis inhibitors. Both PSs led to the release of key DAMPs, which triggered the maturation of DC *in vitro* and provided protective immunity in an animal model.

The following study by Li *et al.* presents a dual-targeting nanosystem designed for effective PDT and PTT combined with immunotherapy by targeting the ER. The nanosystem, comprising ER-targeting pardaxin (FAL) peptide-modified ICG conjugated hollow Au nanospheres (FAL-ICG-HAuNS) and an oxygen-releasing hemoglobin (Hb) liposome (FAL-Hb lipo), addresses hypoxia and enhances treatment efficacy 350. The ER-targeting approach induces significant ER stress and CRT exposure on the cell surface under NIR light, which triggers immune reaction leading to effective tumor growth inhibition and prolonged survival in animal models. Yang *et al.* developed a smart nanovesicle platform (pRNVs/HPPH/IND) to enhance cancer immunotherapy, combining pH-responsive nanovesicles with a PS (HPPH) and an IND 351. These nanovesicles not only deliver therapeutic agents but also induce ICD by exposing CRT, enhancing anti-tumor effects. In a B16F10 melanoma model, this system achieved significant tumor reduction and abscopal effects through ^1^O_2_ generation, improved dendritic cell recruitment, and modulation of the TME by IND, which boosts CD8^+^ T-cell development.

To enhance RT-induced immunity, a biomineralization strategy was developed to synthesize αPD-L1@MnO_2_ NPs, which encapsulate ICB and reprogram the TME 352. The acidic TME triggers the release of Mn²⁺, activating an innate immune pathway to promote dendritic cell maturation and type I interferon production, enhancing tumor-specific immunity. Simultaneously, the released αPDL1 blocks PDL1-PD1 interactions, increasing cytotoxic T lymphocyte (CTL) infiltration and boosting systemic anti-tumor responses. This strategy effectively inhibits primary and metastatic tumors by reversing the immunosuppressive TME, promoting CD8⁺ T cell infiltration, and triggering a robust abscopal effect.

RT has the potential to induce ICD and *in situ* vaccination (ISV) to activate systemic anti-tumor immunity, but its effectiveness is limited by insufficient X-ray deposition and an immunosuppressive TME. Nanoscale coordination NPs (AmGd-NPs) were developed by combining high-Z gadolinium (Gd) for radiosensitization and the CD73 inhibitor AmPCP to improve the immune response towards tumors 353. AmGd-NPs enhance RT by generating ROS to promote ICD, while gradually releasing AmPCP to inhibit CD73 activity. This prevents the conversion of extracellular ATP to adenosine, creating a proinflammatory microenvironment that drives DC maturation and primes CD8⁺ T cell-dependent immune responses. When combined with RT, AmGd-NPs induced potent ISV, inhibited both primary and metastatic tumor growth, and prolonged survival in mice models. This effect was further enhanced by ICB, demonstrating the potential of AmGd-NPs as a promising adjuvant for RT in cancer immunotherapy. To improve the systemic anti-tumor immune response by RT, gadolinium-hemin-based nanoscale coordination polymers (H@Gd-NCPs) were developed as multifunctional RSs 354. These NPs combine high-Z gadolinium for enhanced X-ray deposition and Hemin for GSH depletion and peroxidase-like catalytic activity, amplifying radiation-induced oxidative stress and ICD. H@Gd-NCPs also utilize tumor-overexpressed H₂O₂ to produce ROS, further enhancing their radiosensitization effects. Additionally, the system serves as a MRI contrast agent and can potentiate ICB therapy, strengthening systemic anti-tumor immunity against primary, distant, and metastatic tumors.

**Figure 10:**
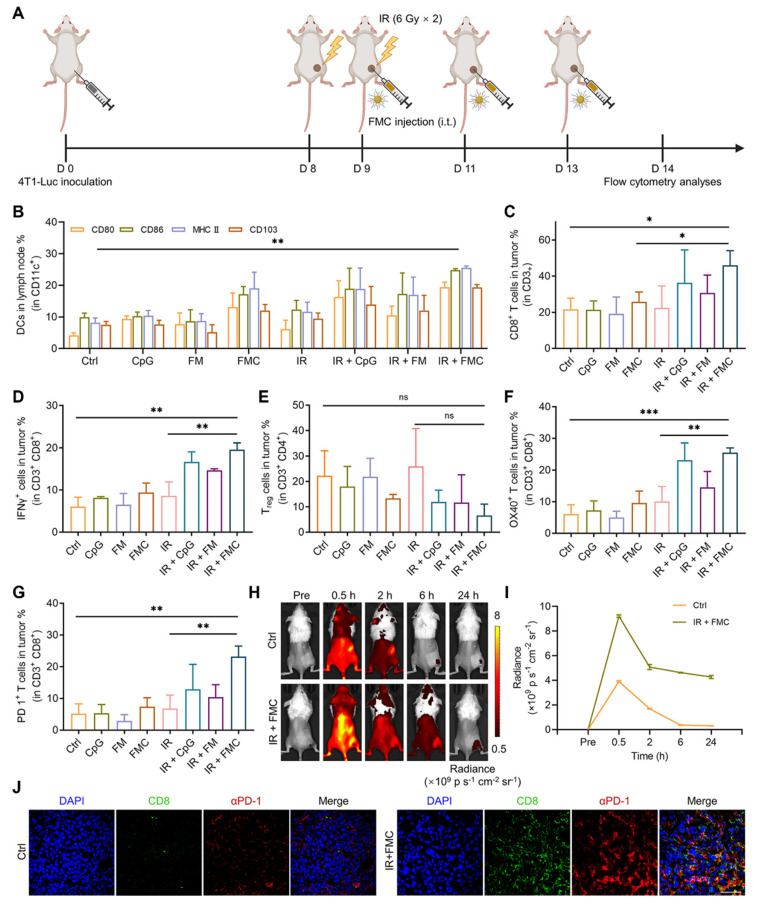
Immune activation effects of FMC-based ISV *in vivo*. **(A)** Experimental setup to assess immune responses triggered by FMC-based ISV, **(B)** percentage of CD11c^+^ dendritic cells in tumor-draining lymph nodes after different treatments, **(C–G)** flow cytometry analysis of tumor-infiltrating T cell subtypes after treatment: CD8^+^ T cells **(C)**, IFN-γ^+^ CD8^+^ T cells **(D)**, Tregs **(E)**, OX40^+^ T cells** (F)**, and PD-1^+^ T cells **(G)**, T cells were identified as CD3^+^CD8^+^, **(H)** *in vivo* FLI of Cy5-labeled αPD-1, **(I)** changes in tumor-site fluorescence intensity over time, **(J)** fluorescence images of tumor sections showing CD8^+^ cells (green, FITC), αPD-1 (red, Cy5), and nuclei (blue, DAPI). Scale bar: 50 μm. Adapted with permission from 356, Copyright 2024 American Chemical Society.

RT-induced abscopal effects are rare in clinical settings due to tumor hypoxia-related radioresistance, insufficient immune stimulation, and an immunosuppressive TME. As an attempt to overcome this, a RT-immunomodulated nanoplatform (THUNDER) was developed to synergize with RT by co-encapsulating TPZ and imiquimod 355. THUNDER targets both hypoxic and normoxic tumor cells, enhancing TAA release and promoting DC maturation. Under RT, hypoxic tumor cells are precisely destroyed by hypoxia-activated TPZ, while normoxic cell death further aggravates hypoxia, amplifying TPZ activation and TAA generation. Simultaneously, R837 enhances immune stimulation in tumors and the spleen, leading to robust systemic immunity and immune memory. In murine models, the combination of THUNDER, RT, and ICB effectively suppresses tumor metastasis and recurrence, demonstrating significant potential for radioimmunotherapy. ISV with intratumorally injected adjuvants offers a promising approach to enhancing the abscopal effects of RT, but its clinical efficacy is limited by insufficient antigen availability from RT-induced ICD. To address this, a novel nanoadjuvant (FMC) was developed, comprising CpG-modified Fe_3_O_4_ NPs with maleimide residues designed to capture TAA via sulfhydryl groups. FMC maximizes antigen bioavailability and immune activation, while its magnetic properties enable tumor visualization through MRI (**Figure 10**). Combined with ICB, FMC-based ISV reverses T-cell exhaustion, promotes DC maturation, expands CD8⁺ T-cells, and reduces suppressive immune cells, effectively suppressing local and distant tumors. Mechanistic studies reveal that FMC disrupts redox homeostasis and alters amino acid metabolism in the TME, inducing ferroptosis and enhancing immune responses 356.

#### NPs enabled PCD pathways

##### Ferroptosis

Ferroptosis is a form of PCD that differs from pyroptosis, apoptosis, and necroptosis, characterized by iron-dependent lipid peroxidation 357, 358. Key parameters that help us understand ferroptosis include iron homeostasis, ROS biology, immunogenicity, amino acid and lipid metabolism, all of which are intricately linked in the process of ferroptosis. Type I ferroptosis involves the inhibition of cystine antiporter system Xc^-^ and type II involves the direct inhibition or degradation of glutathione peroxidase 4 (GPX4). NP mediated ROS based therapies that orchestrate ferroptosis are summarized below 359, 360.

**Figure 11:**
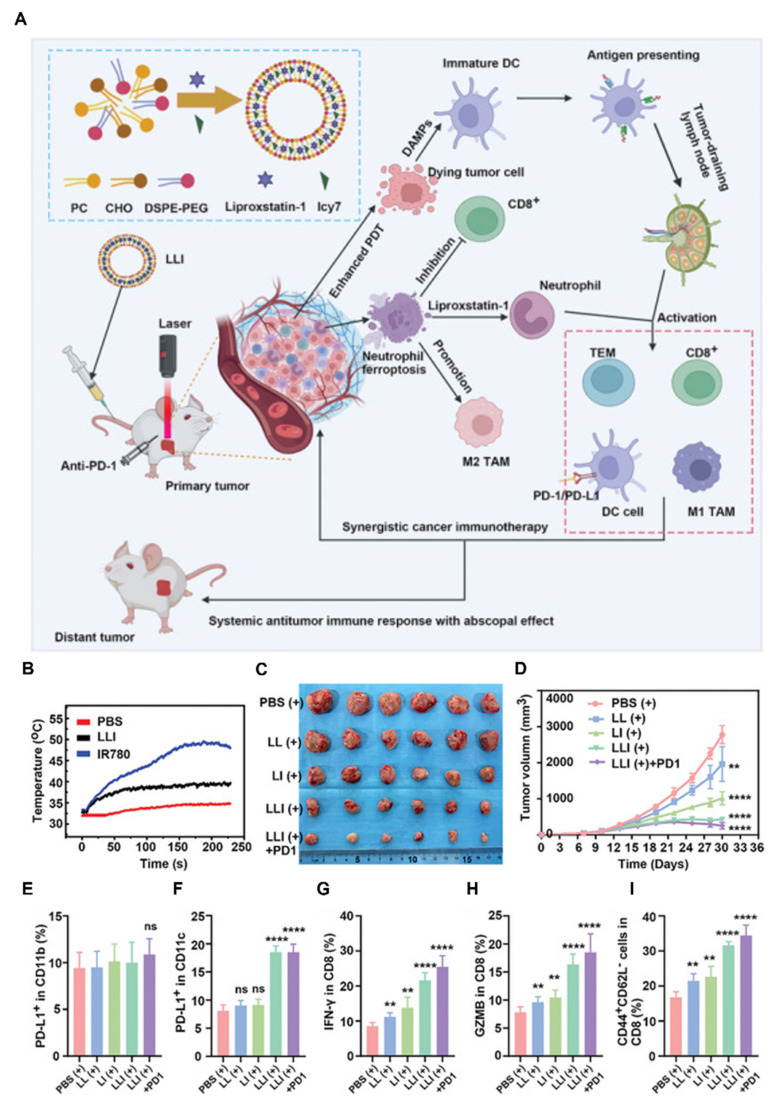
**(A)** Schematic illustration of the preparation of multifunctional nanodrug LLI, its immunogenic PDT, and systemic anti-tumor immunity with abscopal effects, **(B)** tumor temperature changes at different time points after intravenous injection of various solutions followed by laser irradiation (808 nm, 0.5 W·cm⁻²), **(C)** macroscopic images of tumors from mice receiving different treatments, **(D)** tumor growth curves in mice treated with various nanodrugs, **(E, F)** statistical analysis of PD-L1 expression on CD11b⁺ and CD11c^+^ cells in tumor tissues after different treatments, **(G, H)** statistical analysis of IFN-γ and GZMB expression in CD8⁺T cells after treatments, and **(I)** statistical analysis of TEM percentages in spleens after different treatments. Adapted with permission from 361, Copyright 2024 John Wiley and Sons.

For gastric cancer (GC) treatment, targeting the TME has demonstrated promise. Improving the efficacy of PDT using di-iodinated IR780 (Icy7) increases ROS production, thereby inducing ICD and triggering neutrophil ferroptosis (**Figure 11**). A novel nanodrug, LLI, combining a ferroptosis inhibitor (liproxstatin-1) and Icy7, has demonstrated significant inhibition of tumor growth. This approach restores T-cell function by reducing oxidized lipids and boosts anti-tumor immunity when combined with ICB therapy. The synergistic treatment effectively decreased immunosuppression in the TME, promoted DC maturation, increased CD8^+^ T-cell infiltration, and exhibited strong systemic anti-tumor responses, including abscopal effects, emphasizing its potential for GC immunotherapy 361.

To mitigate hypoxia, researchers developed TPEQM-DMA, an organic NIR-II PS with strong type I phototherapeutic efficacy 362. TPEQM-DMA emits in the NIR-II region (> 1000 nm) and produces superoxide anions and •OH through a low-O_2_-dependent process under white light, specifically targeting the mitochondria of cancer cells. This process disrupts cellular redox balance, causes mitochondrial dysfunction, and increases lethal peroxidized lipid levels, resulting in apoptosis and ferroptosis by the downregulation of GPX4. Encapsulating TPEQM-DMA in NPs improved its pharmacological properties, allowing for effective tumor suppression and NIR-II FLI-guided PDT *in vivo*. A core-shell-structured Cu_2_O@Mn_3_Cu_3_O_8_ (CMCO) nanozyme has been developed to serve as a TME-activated copper ionophore for safe and efficient cuproptosis in CRC therapy 363. The Mn_3_Cu_3_O_8_ shell protects normal tissues from Cu_2_O toxicity and improves enzyme-mimicking activities, which deplete GSH levels and generate O_2_, favoring cuproptosis. Furthermore, the Fenton-like reaction from Mn ion release and GSH elimination triggers ferroptosis by the downregulation of GPX4, increasing lipid peroxidation, ROS production and boosts cuproptosis. Mild photothermal effects further improve these enzyme-mimicking activities. CMCO nanozymes exhibit strong catalytic activity, biocompatibility with normal cells, and significant tumor inhibition *in vivo*. ICD through apoptosis or necroptosis improves cancer treatment by triggering anti-tumor immunity; however, tumor resistance to these processes hinders effectiveness. A new photothermal NP (TPA-NDTA NP) has been designed to address this issue, improving ferroptosis induced ICD through excited-state intramolecular motion 364. These NPs, exhibiting strong photothermal conversion and stability, were tested in 4T1 cancer cells and mouse models with poor immunogenic tumors, where significantly improved therapeutic effects through ferroptosis were observed. That study emphasizes the potential of TPA-NDTA NPs in boosting the immunogenicity of ferroptosis.

A pH-responsive nanomedicine (DP-HBN/RA) was designed to act as a RS by amplifying ferroptosis and stimulating the immune system. This nanomedicine uses hollow Bi_2_Se_3_ NPs co-loaded with RSL3 and the STING agonist diABZi, modified with DSPE-PEoz for targeted delivery in acidic TME. It sensitizes tumors to X-rays, generating ROS that induce lipid peroxidation and ferroptosis. RSL3 further intensifies ferroptosis by inactivating GPX4, overcoming RT resistance. Concurrently, the DNA damage and fragments generated by DP-HBN/RA activate the cGAS-STING pathway. DiABZi amplifies this response by increasing the sensitivity of cGAS to DNA fragments, increasing immune reaction towards tumor 365. Nasopharyngeal carcinoma, a head and neck malignancy, often exhibits resistance to RT, resulting in poor prognosis and treatment failure. One study identified circADARB1, a circular RNA significantly upregulated in NPC, as a key driver of RT resistance by inhibiting ferroptosis through the stabilization of SLC7A11 and GPX4. To overcome this resistance, Fe@Pdots-siRNA nanomaterials were developed that combine siRNA-targeting circADARB1 with iron ions, effectively suppressing circADARB1 expression, increasing ferroptosis via lipid peroxidation, and improving radiosensitivity in NPC cells. This nanocomposite not only demonstrated improved tumor targeting efficiency and anti-tumor activity *in vitro* and *in vivo* but also confirmed its biosafety and potential for clinical applications 366.

The clinical application of ferroptosis-inducing drugs remains limited because of safety concerns. Recent research emphasizes the synergistic potential of combining X-ray irradiation with HA-based NPs containing iron (FHA-NPs), which effectively induce ferroptosis by lipid peroxidation. FHA-NPs are internalized via CD44 receptors overexpressed in cancer cells and degraded in lysosomes, thereby triggering lipid peroxidation and ferroptotic cell death. Simultaneously, X-ray irradiation induces DNA damage, with Monte Carlo simulations confirming improved dose delivery in the TME. Both *in vitro* and *in vivo* studies have demonstrated significantly improved tumor control through this combination therapy, providing valuable insights into the mechanisms and therapeutic potential of ferroptosis in cancer treatment 367.

##### Cuproptosis

Cuproptosis is distinct from other forms of oxidative stress-related cell death, such as apoptosis and ferroptosis. It is characterized by mitochondrial stress resulting from the accumulation of lipoylated mitochondrial proteins and the depletion of Fe–S cluster proteins 368-370. CMT for breast cancer often fails due to drug resistance, driven by endogenous and exogenous factors. Cuproptosis, a newly identified form of PCD triggered by copper ion accumulation, provides an alternative approach. However, achieving effective intracellular Cu ion levels is challenging; therefore, researchers have developed PEG@Cu_2_O-ES, a nanocomposite combining elesclomol and cuprous oxide 371. This composite can efficiently enter breast cancer cells, releasing ES and Cu_2_O, which is further accelerated by the NIR-II region PTT effects. Cu_2_O releases substantial Cu ions that participate in the TCA cycle, inducing cuproptosis by the downregulation of DLAT, FDX1, and LIAS proteins. The PTT-enhanced Fenton-like reactions generate ROS, inhibiting the ATP-Cu pump, amplifying cuproptosis. In addition, ES promotes Cu entry from the TME into cancer cells. *In vivo* studies have demonstrated that PEG@Cu_2_O-ES not only exerted strong anti-tumor effects and reprogrammed the TME but also increased sensitivity to αPD-1 immunotherapy. Cuproptosis faces challenges in minimizing Cu ion release in normal tissues and maximizing its therapeutic effect at cancer sites. A photothermally triggered nanoplatform (Au@MSN-Cu/PEG/DSF) was developed for on-demand delivery and synergistic therapy 372. This nanoplatform releases disulfiram to chelate Cu^2+^ and form cytotoxic CuET, inducing apoptosis and promoting mitochondrial protein aggregation, resulting in cuproptosis evidenced by the loss of DLAT and LIAS proteins. Combined with PTT, this approach effectively kills tumor cells and inhibits tumor growth by up to 80.1%. The multifunctional platform ensures controlled payload release at tumor sites, favoring instant intratumoral reactions upon NIR laser irradiation, with negligible effects on healthy tissues.

**Figure 12:**
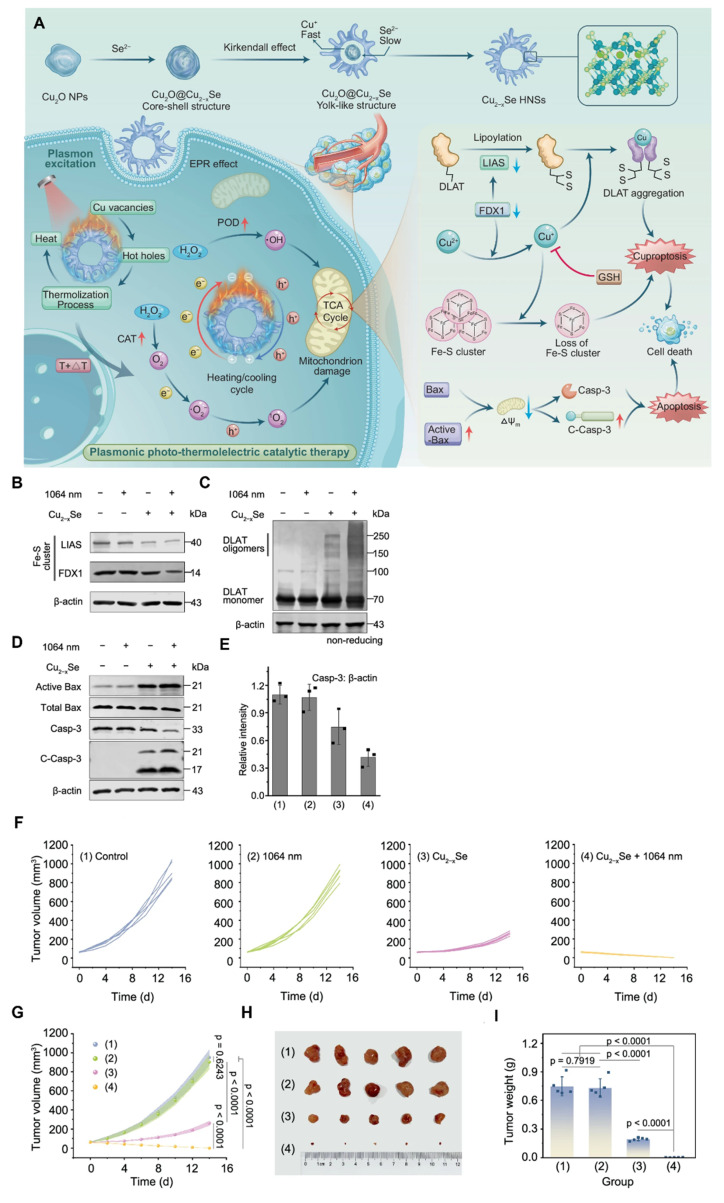
** (A)** Schematic illustration of the synthesis of Cu_2-x_Se HNSs, and anti-tumor mechanism by synergistic therapies, **(B,C)** Western blotting analysis on the expression levels of cuproptosis-related proteins in CT26 cells treated with various conditions, **(D)** the expression levels of apoptosis-related proteins in CT26 cells treated with various conditions examined by western blotting, **(E)** Western blotting intensity ratio of Casp-3 and β-actin, tumor volume **(F, G)** individual and consolidated tumor growth curves of mouse undergoing various treatments, **(H)** photograph of the dissected tumor, and **(I)** tumor weight. Adapted with permission from 375, Copyright 2024 Springer Nature.

Single-atom nanozymes with atomically dispersed active sites are better alternatives to natural enzymes; however, their full potential remains to be studied. A facile synthesis method was developed for sputtered SAzymes, creating copper-based SAzymes (CuSA) with a unique planar Cu-C3 configuration. By improving tumor targeting through a bio-orthogonal approach, the engineered CuSACO demonstrates minimal off-target toxicity and improved multienzyme activities, generating a ROS storm for effective tumor destruction. CuSACO also releases Cu ions in the presence of GSH, inducing cuproptosis by the loss of LIAS, and oligomerization of lipoylated DLAT and improving tumor treatment efficacy. Furthermore, the photothermal properties of CuSACO enable precise PTT, significantly inhibiting orthotopic breast tumors, gliomas, and lung metastasis through combined enzyme-like catalysis, cuproptosis, PTT, and immunotherapy 373. Residual tumors that develop after RT often acquire resistance to radiation, resulting in recurrence, metastasis, and challenges in re-irradiation. FDX1 and LIAS, key regulators of cuproptosis, are upregulated in these tumors, increasing their sensitivity to this form of cell death. To exploit this vulnerability, an interesting radiosensitization strategy was developed using copper-containing nanocapsule-like polyoxometalates, which release copper ions in a controlled manner upon X-ray irradiation. The released Cu⁺ ions trigger cuproptosis by binding to lipoylated proteins in the TCA cycle, resulting in Fe–S cluster protein loss and protein aggregation. This approach effectively reverses radiation resistance, improves tumor immunogenicity, and activates a robust abscopal effect, achieving significant therapeutic efficacy in both local and metastatic tumors in radioresistant and reirradiation murine models 374. Urchin-like Cu_2_–xSe hollow nanospheres enriched with copper vacancies were developed for high-performance plasmonic-thermoelectric catalytic therapy 375 (**Figure 12**). These nanostructures exhibit strong plasmonic absorption and a good PCE (67%) under 1064-nm laser irradiation, generating a localized temperature gradient that activates thermoelectric catalysis. This process improves peroxidase- and CAT-like activities, producing ROS to induce apoptosis in cancer cells. Moreover, the accumulation of copper ions within cells triggers cuproptosis via the loss of FDX1 and LIAS proteins, and promote DLAT oligomerization, generating a synergistic therapeutic effect. Density functional theory calculations reveal that the VCu in Cu2–xSe HNSs improve carrier concentration, electrical conductivity, and enzymatic activities, further increasing therapeutic efficiency. In tumor-bearing mice, this approach demonstrated significant anti-tumor efficacy, using dual pathways—membrane potential reduction and mitochondrial dysfunction—within a single nanostructure. This strategy emphasizes the potential of energy-converting nanomedicines for effective cancer therapy.

A X-ray-activated HfO_2_-based RS (ES@HM-HfO_2_: Cu) designed to enhance tumor RT while minimizing toxicity to normal tissues 376. This nanocapsule system incorporates Cu ions doped into the shell and elesclomol (ES), within its mesoporous structure. Upon X-ray irradiation, Cu ions are specifically released at the tumor site, triggering cuproptosis, this dual sensitization strategy significantly enhances radiosensitivity, leading to a 77.9% tumor inhibition rate. The Cu ions generated during irradiation amplify proteotoxic stress by promoting mitochondrial lipoylated protein aggregation and DLAT accumulation, further amplifying tumor cell death. Compared to conventional HfO_2_ sensitizers, ES@HM-HfO_2_:Cu demonstrates better efficacy and safety, providing a strong rationale for clinical applications.

##### Pyroptosis

Pyroptosis is a type of RCD triggered by disturbances in innate immunity. Pyroptosis is characterized by its dependence on inflammatory caspases (CASP) for its execution. This form of cell death can be initiated by various inflammatory or apoptotic caspases, such as CASP1, murine CASP11, human CASP4 and CASP5, and CASP3. Pyroptosis begins with the assembly of an inflammasome, a multiprotein complex that includes a sensor, the adaptor ASC, and CASP1. This complex facilitates the activation of CASP1, which then processes its substrates, including pro-IL-1β and pro-IL-18, into their active forms, and GSDMD, which forms pores in the plasma membrane 377, 378.

A novel nanoplatform integrating AIE luminogen (AIEgen) and a pyroptosis inducer into hypoxia-responsive covalent organic frameworks (COFs) was developed for improved image-guided cancer immunotherapy 379. By synthesizing and comparing donor-acceptor type AIEgens, it was found that incorporating two acceptor units provided the longest response wavelength and most effective PDT. The constructed COF-based nanoplatform facilitates hypoxia-triggered COF degradation, improving fluorescence and PDT properties and accelerating the release of the pyroptosis drug. This system enables precise tumor imaging and effective tumor inhibition through synergistic PDT and pyroptosis by the upregulation of cleaved caspase-3 and the cleavage of GSDME into GSDME-N, leveraging exceptional ROS production and controlled drug release. In 4T1 tumor-bearing mice, the nanoagent effectively inhibited primary and distant tumor growth, demonstrating its potential as a self-synergistic cancer immunotherapy approach. The absence of tumor antigens, a challenge in ICB therapy, can be addressed by inducing tumor-specific pyroptosis. To achieve this, an ROS/GSH dual-responsive nanoprodrug loaded with paclitaxel and the PS purpurin 18 (P18) was designed 380. This chemo-PDT system responds to the high ROS/GSH levels in the TME, enabling controlled drug release and inducing pyroptosis through increased levels of GSDME-N. Pyroptotic tumor cells release DAMPs, increasing ICB efficiency, initiating adaptive immunity, and preventing tumor recurrence. MCPP exhibits high tumor retention and penetration, promoting DC maturation and T-cell expansion and generating immunological memory.

**Figure 13:**
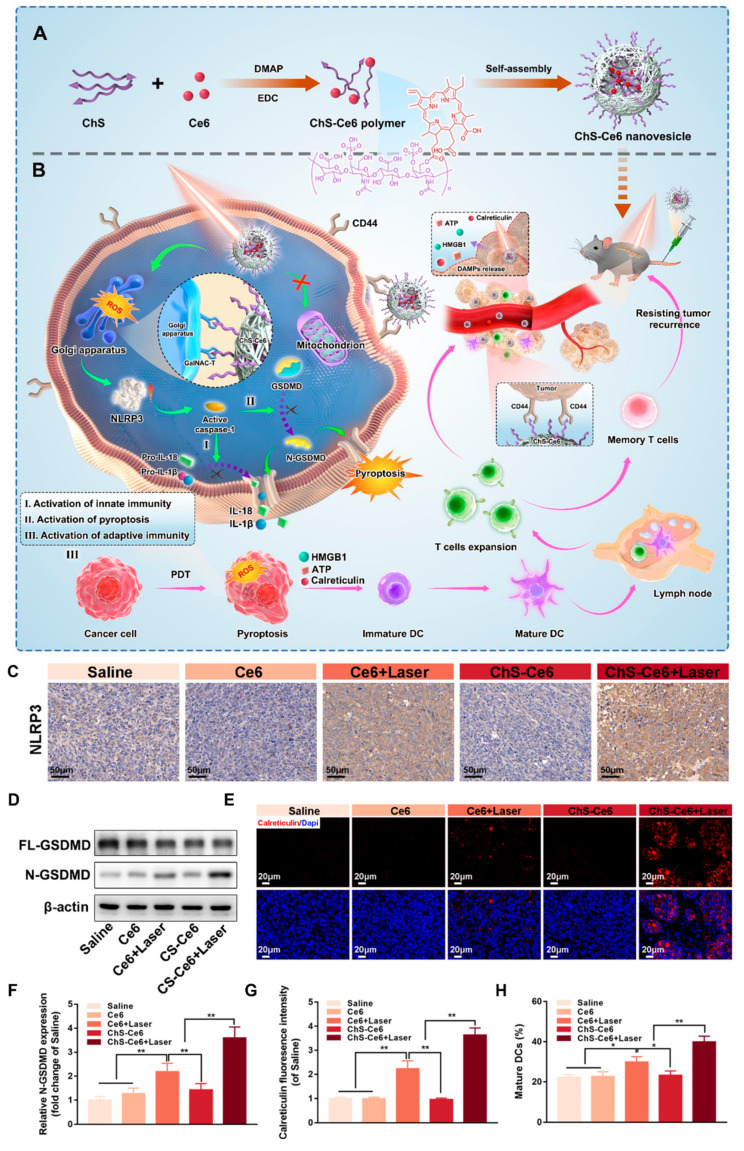
**(A,B)** Schematic illustration of the synthesis and application of Golgi apparatus-targeted ChS-Ce6 nanovesicles for NLRP3-dependent immunogenic pyroptosis to remodel the tumor microenvironment, **(C)** representative immunohistochemical staining of NLRP3 in tumor tissues, **(D, F)** Western blotting and quantification of N-GSDMD expression in tumor tissues **(E, G)** immunofluorescence staining and quantification of CRT expression in tumor tissues and **(H)** flow cytometry quantification of mature DC in tumor tissues. Adapted with permission from 381, Copyright 2023 American Chemical Society.

Golgi apparatus plays a vital role in NLRP3 activation. A Golgi apparatus-targeted photodynamic strategy was developed using self-assembled ChS-Ce6 nanovesicles to induce NLRP3 activation precisely 381. This approach significantly upregulated NLRP3, resulting in an inflammatory response that improved innate immunity and triggered downstream caspase-1-dependent pyroptosis. This process promotes ICD, DC maturation, and robust anti-tumor immunity and inhibits distant cancer development (**Figure 13**).

To improve pyroptosis-mediated PDT, Z1, an NIR-II PS with a thiopyrylium framework and high benzene ring count, was developed. Z1 exhibits superior type I ROS generation, and avid mitochondrial-targeting capabilities. Encapsulated into biocompatible NPs (Z1 NPs), this system demonstrates precise tumor targeting, guided by NIR-II FLI and PAI. Upon 808-nm laser irradiation, Z1 NPs induce mitochondrial dysfunction, triggering both pyroptosis by the upregulation of cleaved caspase-3 and GSDME-N and apoptosis to effectively suppress tumor growth. *In vivo* studies confirmed the high therapeutic efficacy of Z1 NPs with minimal side effects 382. Macrophage-based biohybrid microrobots (IDN@MC) present a promising strategy for breast cancer treatment by inducing targeted pyroptosis and improving anti-tumor immunity 383. These microrobots combine macrophages with pH-sensitive decitabine-loaded MOFs (DZNPs) and fluorescent PS, thus enabling photothermal conversion, fluorescence navigation, targeted drug delivery, and controlled release. The microrobots accumulate at tumor sites, where DZNPs release decitabine to polarize macrophages into the M1 phenotype for tumor cell phagocytosis and direct delivery of therapeutic agents. Under laser irradiation, the microrobots induce pyroptosis via the upregulation of cleaved caspase-3 and GSDME-N, releasing inflammatory factors, promoting DC maturation, T-cell expansion, and immune microenvironment modulation. This robust immune activation inhibits tumor growth, prevents metastasis, and primes adaptive immunity.

A novel nanocomposite RS, DAC@O-HONs, composed of ultrasmall HfO_2_ NPs loaded with decitabine to transform radiation-induced apoptosis into pyroptosis for treating TNBC was designed and developed 384. The HfO_2_ NPs improve tumor targeting through efficient osmosis and retention along with generating ROS under ionizing radiation to induce apoptosis. Simultaneously, DAC reverses GSDME silencing by inhibiting DNA methyltransferase, converting apoptosis into pyroptosis. This process releases TAA and mounts immune reaction towards tumor to inhibit tumor metastasis and recurrence.

While initial nanomedicine research focused primarily on the induction of apoptosis, contemporary evidence suggests that advanced nanosystems often elicit a hybrid cell death landscape. For instance, while ROS-generating NPs may trigger rapid necrosis, the same system at a modulated concentration might initiate ferroptosis via GPX4 inhibition or pyroptosis through NLRP3 inflammasome activation. The emerging paradigm of cuproptosis specifically induced by copper-ionophores and copper-based NPs further complicates this hierarchy by targeting mitochondrial respiration (**Table 6**). Understanding this link is essential, as the simultaneous activation of multiple RCDs by a single NP platform can prevent the compensatory survival mechanisms typically employed by heterogeneous tumor populations.

#### NP promoting STING pathway

Recently the significance of the cyclic GMP-AMP synthase (cGAS)–STING pathway in activating anti-tumor immunity and transforming immunologically inactive ("cold") tumors into responsive ("hot") tumors has inspired researchers. Cytosolic double-stranded DNA (dsDNA), typically associated with damage, is detected by cGAS, leading to the production of cyclic GMP-AMP (cGAMP) 385, 386. cGAMP acts as a second messenger to activate STING, prompting the release of type I interferons (IFNs), crucial for stimulating both innate and adaptive immune responses. Beyond its established role in microbial defense, recent studies highlight the importance of the cGAS–STING pathway in cancer progression, genomic instability, and shaping the TME. Therapeutically targeting STING with agonists has demonstrated promising preclinical anti-cancer efficacy, highlighting its potential in cancer immunotherapy strategies. Here, we introduce the design and applications of nanomedicine that could either deliver cGAS-STING activators or possess intrinsic capability to induce STING 387, 388.

One study introduced novel bimetallic nanopolymers (TA-Fe/Mn-OVA@MB NPs) that combine PTT and PDT to target tumors 389. These nanopolymers, formed by assembling tannic acid, bimetals, and ovalbumin, demonstrate excellent dispersity and biocompatibility. Under specific laser irradiation, they effectively destroy tumor cells and promote the release of tumor antigens. Furthermore, they improve the immune response by activating the cGAS-STING pathway, resulting in the maturation of DC and the activation of CTL, thus providing a potent strategy for combining localized phototherapy with robust anti-tumor immune activation. A bioactive injectable hydrogel (BG-Mngel), composed of manganese-doped bioactive glass and sodium alginate, was developed as a versatile platform for simultaneous anti-tumor immunotherapy and wound healing. The Mn^2+^ in BG-Mn activates the cGAS-STING pathway, eliciting a robust immune response, whereas its photothermal properties improve NP uptake in tumor cells and facilitate STING activation under NIR light.

This synergistically inhibits tumor growth and prevents recurrence, particularly when combined with the α-PD-1, which strengthens long-term immune memory. Moreover, BG-Mngel promotes angiogenesis and tissue regeneration by releasing bioactive ions such as SiO_4_^4-^, Ca^2+^, and Mn^2+^, enabling rapid wound healing in melanoma models 390. The cGAS-STING pathway plays a vital role in tumor immunotherapy; however, the lack of tumor-specific targeting by STING agonists has limited its clinical application. A photodynamic polymer was designed to encapsulate a cationic Pt (II) agent (56MESS), forming NPs (NPPDT-56MESS) 391. These NPs accumulate at tumor sites and, upon 808-nm laser irradiation, generate ROS that disintegrate the NPs and release 56MESS. The ROS and 56MESS synergistically damage DNA and mitochondria, which activates the cGAS-STING pathway to induce robust anti-tumor immune responses. In mouse models of uveal melanoma, NPPDT-56MESS demonstrated effective tumor targeting, reduced systemic toxicity, and induced systemic anti-tumor immunity, including immune memory, which prevented tumor recurrence and metastasis. Researchers have developed a 2D nanoplatform, cGAMP/MOL, where cGAMP is conjugated to a nanoscale MOL 392. This design improves both STING activation and radiosensitization within tumors. Upon X-ray irradiation, cGAMP/MOL induces ICD and improves the maturation of APCs, resulting in a “hot” tumor immune environment rich in inflammatory cytokines and type I IFNs. This environment fosters local innate and adaptive immune responses. When combined with ICB, cGAMP/MOL overcomes immune suppression, reinvigorates T cells, and bridges innate and adaptive immunity, resulting in systemic anti-tumor effects and tumor regression.

This study introduces an innovative NP-based immunotherapy designed to target pulmonary APCs and improve anticancer responses against lung metastases. In mouse models, inhaling phosphatidylserine-coated liposomes loaded with the cGAMP allows for rapid distribution across the lungs, where the NPs are rapidly absorbed by APCs without triggering harmful immune responses 393. The formulation is optimized for efficient cytosolic release of cGAMP, which activates STING signaling and promotes the production of type I IFNs in APCs. This activation transforms the TME into a proinflammatory state across multiple lung metastases. In addition, when fractionated radiation is applied to one of the tumor-bearing lungs, synergy was established with the NP-cGAMP, resulting in a robust systemic anticancer immune response, even when exposed to repeated tumor challenges.

An ECM-degrading nanoagonist (dNAc) was designed for targeted breast cancer therapy. The dNAc contains FeS_2_ NPs that serve as NIR-II photothermal converters and Fenton catalysts, along with a STING agonist and an ECM-degrading enzyme 394. Mild heat generated by the dNAc after irradiation improves the Fenton reaction, inducing tumor cell death, ICD, and STING activation. Robust immune reactions effectively inhibited both primary and distant tumors in, along with suppressing liver and lung metastasis. A smart nanosystem (PBM) was developed based on Prussian blue NPs doped with Mn(III). This system improves the photothermal ablation-induced activation of the cGAS-STING pathway by generating cytosolic DNA damage and continuously releasing Mn(II) in response to the GSH-rich environment of the tumor 395. The PBM nanosystem not only improves local tumor ablation but also triggers a strong systemic immune response, effectively reducing the risk of tumor recurrence and metastasis.

**Figure 14:**
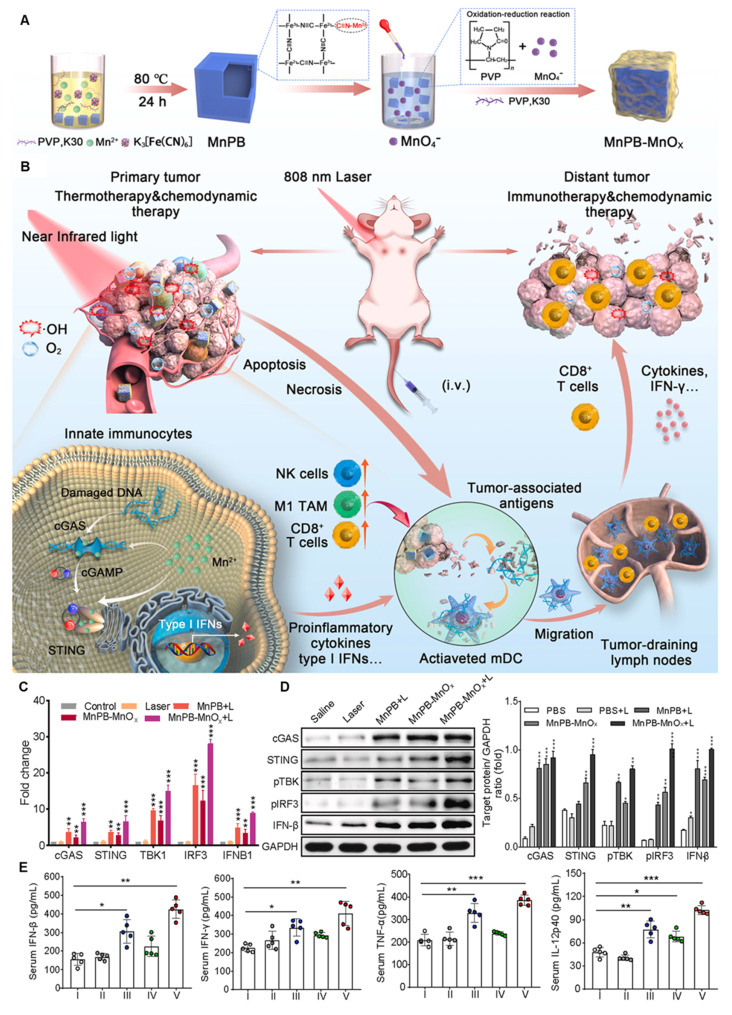
** (A)** Schematic illustration of the preparation of MnPB-MnOx nanosystems, **(B)** mechanism of anti-tumor immune responses induced by combined noninvasive photothermal ablation with Mn²⁺-augmented cGAS-STING pathway activation for effective cancer immunotherapy, **(C)** QRT-PCR analysis of cGAS, STING, TBK1, IRF3, and IFNB1 expression in primary tumors after the indicated treatments, **(D)** expression levels of cGAS-STING-related proteins, and **(E)** cytokine levels in the serum of mice in each group on day 10 after various treatments. Adapted with permission from 396, Copyright 2023 Elsevier.

This study presents a novel approach for treating TNBC by combining PTT with Mn^2+^- to activate the cGAS-STING pathway. The researchers developed a manganese-enriched nanoplatform that integrates Mn-doped Prussian blue NPs with MnOx 396. This platform leverages Mn^2+^ to amplify the cGAS-STING pathway and uses hyperthermia and ROS to elicit immune response against tumor. Experiments using 4T1-bearing mice demonstrated that this treatment effectively suppressed both local and distant tumors by promoting innate and adaptive immunity (**Figure 14**). In another study, a multifunctional bionic nanoplatform was designed to improve the treatment of PCa by combining PDT, CMT, and immunotherapy. The nanoplatform incorporates the AIE PS TPAQ-Py-PF6 and PTX, in a macrophage cell membrane for active targeting 397. Upon laser irradiation, the platform generates ROS that cause DNA damage, triggering ICD and activating the cGAS-STING pathway to mount a robust systemic immune response. The combination of PDT and CMT not only improves the anti-tumor effects but also transforms the TME from “cold” to “hot,” boosting cytotoxic T-cell infiltration and establishing lasting immune memory.

A polymeric metal-organic framework (PMOF) NPs was designed to improve cancer immunotherapy by combining PDT with STING activation 398. The porous structure of PMOF NPs, could accommodate a STING agonist (SR-717) and coated with a PEG shell. Upon intravenous injection and tumor targeting, light irradiation at the tumor site triggers the production of ROS cleaves thioketal bonds within the PMOF releasing SR-717, which activates the STING pathway. This dual action not only reverses the immunosuppressive TME but also improves anti-tumor immunity, effectively suppressing both primary and metastatic tumors. A manganese-doped layered double hydroxide (Mn-LDH) nanoplatform was developed using a hydrothermal method to reprogram the TME and potentiate anti-tumor immunity. Mn-LDH neutralizes protons and consumes hydrogen peroxide to produce oxygen, generating a positive feedback loop that releases Mn²⁺ and Mg²⁺ 399. These ions activate the cGAS-STING pathway and sustain CD8⁺ T-cell cytotoxicity, triggering a cascade of innate and adaptive immune responses. Locally administered Mn-LDH transformed the TME into an immune-supportive state, forming a network of mature DC, M1 macrophages, and cytotoxic and helper T cells, thereby effectively inhibiting tumor growth and metastasis in breast and colon tumor models. Furthermore, its photothermal conversion capability improved the therapeutic efficacy in large tumor models with a single administration and irradiation. To mitigate hypoxia-associated radioresistance in rectal cancer (RC), researchers have developed a catalytic RS, DMPtNPS, combined with a STING agonist, to improve RC radiosensitivity 400. The combination of DMPtNPS@cGAMP, RT, and ICB resulted in a durable complete response at the primary tumor site and an improved abscopal effect on distant tumors in a bilateral tumor model.

A nanotheranostic platform combining PDT and epigenetic therapy to improve cancer treatment by inducing pyroptosis and activating the cGAS-STING pathway in a light-controlled manner was developed. The system involves two types of NPs, viz., NP1, which is ROS-sensitive and loaded with the PS TBE, and NP2, which contains the epigenetic drug decitabine. NP2 reverses promoter methylation, restoring STING and gasdermin E (GSDME) expression, whereas NP1 generates excessive ROS under 808-nm laser exposure, causing mitochondrial damage and the release of DNA fragments. This stimulates the cGAS-STING pathway and activates caspase-3 to cleave GSDME into its pore-forming fragment, initiating pyroptosis^401^. A strong anti-tumor immune response is then triggered by the release of inflammatory cytokines, which enhance T-cell-mediated immunity and long-term immunological memory. Additionally, the system uses NIR fluorescence bioimaging to provide TME regulation and guided photoimmunotherapy. SAPTN was designed and developed to improve cancer immunotherapy by targeting the STING pathway. The SAPTN is engineered with NIR-II properties for precise imaging and PAI-guided therapy. It delivers the STING agonist diABZIs in a sustained manner within the tumor’s ECM, ensuring durable drug activity. When combined with mild PTT, which triggers neutrophilic inflammation, SAPTN improves tumorous retention and stimulates a robust and long-lasting immune response. This strategy achieved tumor-free survival for at least two months in preclinical studies, when nearly 100% of mice with breast cancers had their tumors completely eliminated. When given locally, SAPTN also had an abscopal impact, slowing the growth of distant tumors. Additionally, NIR-II imaging makes it possible to clearly identify the tumor margin for improved treatment guidance.^402^. A MOF-based nanoagonist (DZ@A7) for precise tumor-specific activation of the cGAS-STING pathway and improved photodynamic–metalloimmunotherapy was developed 403. Inside the cancer cells, when exposed to NIR irradiation, DZ@A7 performs several functions such as i) generates mitochondrial-targeted ROS, ii) releases oxidized mitochondrial DNA (mtDNA) into the cytoplasm, activating the cGAS-STING. Simultaneously, hypoxia-responsive drugs inhibit DNA repair, resulting in increased cytosolic damaged DNA, further boosting immune activation. Furthermore, NIR-accelerated zinc ion overloading improves cGAS enzymatic activity, amplifying STING activation. The combination of these effects promotes DC maturation and cytotoxic T-cell infiltration, causing the eradication of primary tumors and establishing long-term anti-tumor immunity to prevent metastasis. **Table 5** shows NP mediated cell death pathways and innate immune system activation.

#### Toxicity of NPs

Nanotoxicity can be ascribed to their rate of elimination. Glomerular endothelial cells (GECs) is a part of RES, which is integral to the glomerular filtration mechanism. GECs contain numerous fenestrae with diameters ranging from 60–80 nm, which facilitates rapid exchange of macromolecules between the vascular and extravascular spaces 404, 405. Inorganic NPs with a size less than 6 nm and organic NPs with size cutoff 8-10 nm are eliminated from the body through renal mechanism 406, 407, while large sized NPs is trapped in the GECs base membrane 408. NPs can also be eliminated through bodily secretions such as saliva, breast milk and sweat, but this warrants large-scale studies for better understanding 409-411. NPs that do not undergo renal elimination obliges to hepatobiliary excretion, which is size and charge dependent. As demonstrated in a study, both MSN with more charge (+34.4 mV) and less charge (-17.6 mV) are accumulated in the liver, former undergoes hepatobiliary excretion into gastrointestinal tract while latter remains sequestered 412.

Metabolism of NPs strongly depends on their organic and inorganic composition, among them, inorganic NPs show a high propensity to liver accumulation causing toxicity 413. Inorganic NPs tend to degrade and release constitutive ions in the acidic lysosome compartment, which react with biomolecules, altering their biological activity 414. Whereas organic counterparts, including lipid, polymeric NPs, show biotransformation rates, the resulting products may be more reactive or present low clearance rates. In addition, biotransformation rates are correlated to drug release profile. Accelerated biodegradation triggers untimely drug release, while slow degradation can lead to prolonged tissue accumulation and potential toxicity. Orally administered TiO₂ NPs interact with the gut microbiota affecting gut-associated metabolism, altering several metabolites and metabolic pathways. In particular, the levels of metabolites such as N-acetylhistamine and glycerophosphocholine increased, while others including L-histidine and L-ornithine decreased. This may lead to enahnced lipopolysaccharide production, which subsequently triggers oxidative stress and inflammatory responses in the intestine 415. *In vitro* studies using TM-4 Sertoli cells and GC2-spd spermatocyte cells demonstrated that ZnO NPs can penetrate cells, disrupt cell membranes, and generate oxidative stress, that activates the ROS–ERK–TNF-α signaling pathway, leading to impairment of the blood–testis barrier by reducing junction proteins in Sertoli cells. In germ cells, ROS generation causes DNA damage, reduced DNA repair capacity, and S-phase cell cycle arrest, contributing to genotoxic effects 416. Small amorphous silica NPs (~70 nm) can trigger strong coagulation responses after systemic exposure. In mice, i.v. injection caused platelet depletion, liver injury, and fatal coagulation reaction. This effect occurs through activation of the intrinsic coagulation pathway via interaction with coagulation factor XII, which is enhanced by the large surface area of smaller NPs 417.

## Conclusions and outlook

This review has comprehensively summarized the development of nanomaterials for precise nanotheranostics from optical to radioactive modalities. Imaging-guided photoinduced treatment has achieved enormous advancements in recent decades and plays an increasingly vital role in the global war against cancer. Photo-based therapies for cancer that exert minimal side effects and high specificity can effectively decrease side effects. Considering the tissue penetration limitation of PDT and PTT, tremendous attempts have been devoted to exploring deep light delivery techniques for treating orthotopic tumors inside patients. Very recently, compared with conventional NIR-I imaging, imaging in the NIR-II region has demonstrated superior performance to that in the NIR-I region due to its higher spatial resolution, deeper penetration depth, and lower optical absorption and scattering from biological substrates with minimal tissue autofluorescence. With this strong motivation, several novel NIR-II imaging systems have been applied to assist surgeons in precision surgery.

Moreover, due to the high tissue-permeability of X-ray radiation and interstitial fiberoptic light sources, several investigations on orthotopic tumor models were performed within the past 2 years. Consequently, clinical applications of X-ray PDT or interventional PTT are reasonable in the near future. The incorporation of imaging techniques into RT has the potential to allow dose painting with minimal toxicity in the surrounding normal tissue and maximum therapeutic dose in localized tumors. Currently, NPs, such as gadolinium-based NPs (AGuIX) and HfO_2_ NPs crystalline NPs (NBTXR3), in conjunction with RT are being used in clinical trials in imaging-guided RT. In particular, NBTXR3 and HfO_2_ NPs, combined with IMRT, for the treatment of PCa are under Phase I/II investigation (NCT02805894). Furthermore, the clinical applications of IMPT, as well as IMRT, are rapidly increasing for the treatment of various cancers. Although IMPT has achieved encouraging results and cost-effectiveness in several cancers, additional clinical data are necessary. Apart from extrinsic RT, another methodology used to achieve deeper tissue penetration is the utilization of radionuclides as an internal activation light source. In 2015, Achilefu *et al.* demonstrated that tumors could be eliminated through CR-triggered NPs. To date, CLI, which combines nuclear and optical imaging, has been used in intraoperative FLI-guided surgery, including breast cancer, PCa, gastrointestinal cancer, and metastatic lymph nodes. Furthermore, NPs and microparticle-based imaging-guided theranostic agents have entered clinical oncological applications. For instance, liver radioembolization has achieved successful clinical trials. To date, ^90^Y Ms are approved in the European Union, Asia, and the USA for the treatment of unresectable liver tumors, and more than 40,000 treatments have been performed using ^90^Y-labeled Ms in hepatic solid tumors. Numerous novel particles are currently under development for next-generation imaging-guided theranostic agents, and successful clinical cases will accumulate with time. Nanotechnology has greatly advanced imaging-guided treatment of deep-seated tumors, improving both tumor detection and targeted therapy. Emerging imaging systems such as total-body PET and MARS-CT are enhancing sensitivity, quantification, and scan speed, which may allow earlier and more accurate diagnosis. On the therapeutic side, photoresponsive and radioactive NPs have shown strong potential for highly specific tumor ablation.

NPs have emerged as powerful tools due to their ability to interact with cellular components and modulate various biochemical pathways. We emphasized their role in inducing specific cell death mechanisms such as ferroptosis, pyroptosis, and cuproptosis, each characterized by distinct molecular processes orchestrated by PDT, PTT, and RT. These cell death modalities instigated by either oxidative or mitochondrial proteotoxic stress have the potential to induce an immune response by promoting the release of tumor cell–specific antigens and cause ICD that could improve cancer immunotherapies. NPs have also emerged as potent modulators of the innate immune system, and are designed to overcome the delivery issues of STING agonists such as low bioavailability, poor *in vivo* circulation times and modest uptake by the tumor cells due to their negative charge 418-420. To overcome the challenges associated with the i.v. delivery of STING agonists, Dosta *et al.* conjugated STING agonist CDN to poly(β-amino ester) (CDN NPs) through a cathepsin-sensitive linkage. The synthesized polymer-drug conjugate showed improved drug loading, good serum stability and low drug dose, which was sufficient to exhibit robust anti-tumor therapeutic response across various syngeneic tumor models and elicit immune memory. Tumor acts as a drug depot for CD NPs and releases them to nearby immune cells to activate STING and inhibit tumor growth 421. Another interesting approach is leveraging the potential of Mn²⁺ ions, which can activate monomeric cGAS even without dsDNA and does not require cGAS oligomerization. Mn²⁺ and cytosolic dsDNA together enforce each other’s binding to cGAS, markedly lowering the activation threshold of the STING pathway. Mn²⁺ increases the enzymatic activity of dsDNA-bound cGAS, leading to higher production of the second messenger cGAMP. Mn²⁺ can also trigger phosphorylation of TBK1 and p65 independently of STING and, together with STING agonists, promotes enhanceosome formation and stronger IFN-β production 422-424. In a recent study, blackphosphorous nanosheets were modified with Mn^2+^/CpG oligodeoxynucleotides (BPNS@Mn^2+^/CpG) through coordination bond. In TME, Mn^2+^/CpG were released in a pH responsive manner activating immune cells to prime cytotoxic and helper T cells via Toll-like receptor 9 and STING pathways. Phototherapy combined chemodynamic therapy of tumor by BPNS@Mn^2+^/CpG induced ICD that not only suppressed the primary tumor but also inhibited the distant tumor growth 425. A polymer based universal STING mimic (uniSTING) was developed that can self-assemble into active STING moieties and trigger STING signaling independent of endogenous STING expression. When delivered as mRNA through lipid NPs, uniSTING effectively activated the TBK1–IRF3–IFN-I pathway in both tumor cells and dendritic cells. It also promoted dendritic cell maturation and stronger CD8⁺ T-cell responses, leading to significant tumor suppression in several cancer models 426.

A plethora of NPs have been synthesized, optimized, and demonstrated potential in preclinical levels. Nevertheless, their clinical translation is severely hampered by both immune and physiological barriers. Inside the body, based on the surface composition, NPs are covered by serum proteins known as opsonin, which are readily recognized and cleared from circulation by mononuclear phagocytic systems or reticuloendothelial systems. The transport of NPs to tumors occurs through inter-endothelial gaps in tumor blood vessels and retained by a poor lymphatic system, termed as the EPR effect. Nevertheless, the existence of the EPR effect is heterogeneous and dependent on tumor types. A relatively novel mechanism for NP entry into tumors, termed active transcytosis, has been proposed using Au NPs. This process involves NPs transport through endothelial cells and overexpression of transport pathway–related genes. Once inside the tumor, the NPs are retained through interactions with TME components, such as TAMs and ECM, rather than depending on poor lymphatic drainage. Furthermore, extensive investigations are required to validate the mechanism using soft NPs and various tumor models. Active tumor targeting involves engaging receptors expressed on tumors through ligand-modified NPs. The expression level of receptors and ligand density on NPs plays a vital role in cancer cell–specific uptake of NPs. To accelerate the clinical translation of NPs, regulatory bodies such as the FDA USA and the European Medical Agency have issued guidelines to ensure the safety, efficacy, and quality of NPs and emphasize the characterization of physicochemical properties, pharmacokinetics, and potential toxicological effects; however, current frameworks face challenges in addressing the complexities of nanoscale materials. Advances in computational tools, particularly AI-based platforms such as AlphaFold and Rosetta, have transformed the landscape by enabling precise protein structure prediction and novel protein design. These innovations provide critical insights into protein–ligand interactions and facilitate the development of targeted nanomedicine therapies with optimized safety and efficacy. The use of human-relevant experimental systems, including patient-derived organoids, 3D tumor models, and organ-on-chip platforms, can provide more realistic biological conditions than conventional 2D cell culture or murine models 427-429. Improved animal models, such as orthotopic tumors, genetically engineered mouse models and patient-derived xenografts, can better mimic disease complexity and drug responses 430-432. Improving patients’ quality of life can expand the number of people willing to receive treatment, which also boosts the practical and commercial value of a therapy. For example, treatments that provide better effectiveness, cause fewer side effects, require fewer doses, and can be given through convenient routes of administration are more satisfactory to patients. These factors can improve patient adherence to therapy and ultimately allow a larger patient population to benefit from the treatment.

Toxicity of the NPs is a crucial parameter impeding their clinical translation, profound accumulation of NPs in undesired vital organs is a major contributor to toxicity. NP often accumulates in organs such as the liver, lungs, kidneys, spleen, and bones. This is mainly because these organs contain cells of the reticuloendothelial system that capture and remove foreign bodies from the blood 433. The extent of this accumulation depends on the NPs properties, including their size, surface charge, stiffness, and other physicochemical characteristics 434. PEGylation is commonly used to give NPs a “stealth” property. It reduces opsonization by proteins in the bloodstream and prevents the particle aggregation 435, 436. In addition, nanomedicines should be screened using human blood or serum complement assays, cytokine profiling, and more predictive models such as the porcine CARPA model, which has been widely used to study acute cardiopulmonary infusion reactions 437-439. From the industry point of view, NPs bulk production is challenging due to their complex design, composition, and multistep synthesis that increases the production cost and time. Preclinical purification methods, such as ultrafiltration and evaporation, are often not efficient during large-scale production and may leave behind toxic solvent residues 440, 441. By incorporating such tools into regulatory processes, the evaluation of nanomedicine interactions and off-target effects can be significantly improved, ultimately enhancing the therapeutic outcomes and advancing regulatory science.

## Figures and Tables

**Table 1:** Deep tissue PDT nanosystems and their experimental parameters.

Energy modes	NPs (Size, zeta potential and morphology)	Treatment condition, λ power	Biological experiment	Ref.
Upconversion	UCNPs@mSiO_2_-ZnPc-RBS, ~200 nm, hexagonal shape	1550, 808 nm and 980 nm; 1 W•cm^-2^	4T1 subcutaneous xenograft (i.t.), tumor inhibitory effect	60
	NaGdF_4_:Yb Tm@CaF2:Eucore@shell, ~150 nm, spherical shape	980 nm; 2 W•cm^-2^	GBC orthotopic xenograft (i.v.), tumor inhibitory effect	61
	PB@VA, 100.37 ± 4.05 nm, ~-30 mV	980&808 nm; 1 W•cm^-2^	HeLa subcutaneous xenograft (i.t.),tumor inhibitory effect	63
TP	Emo/HSA NPs (E/H NPs), 315 ± 158.1 nm, spherical shape	800 nm; 2 W.cm^-2^	MCF7 subcutaneous xenograft (i.v.), tumor inhibitory effect	73
	NIR-TADF NPs, ~140 nm, -46.1 mV, spherical shape	800 nm; 2 W.cm^-2^	A549 subcutaneous xenograft (i.v.), tumor inhibitory effect	75
	DSPE-PEG-Biotin@2-O-IrAn, ~116 nm, spherical shape	750 nm, 50 mW.cm^-2^	A549 subcutaneous xenograft (i.v.), tumor inhibitory effect	76
X-PDT	ITC NPS, 47.2 ± 0.8 nm, -1.0 mV, spherical shape	0.4 Gy; 50 kV	4T1 subcutaneous xenograft (i.t. and i.v.), tumor inhibitory effect	89
	Sc-D@Lip/Pt, 73 nm, hexagonal shape	2 Gy	4T1 subcutaneous xenograft (i.v.), tumor inhibitory effect	90
	NSs-RB-F/cRGD, 274.2 nm, -40.3 mV, Hexagonal shape	0.025 Gy	NMIBC (intravesical fusion), tumor inhibitory effect	93

**Table 2:** NIR-I and II image-guided PTT nanosystems.

Energy modes	Imaging technique	NPs (Size, zeta potential and morphology)	Exposer conditions for PTT	Biological experiment	Ref
NIR-I	FL	Water heating NIR NP, 18.5 ± 1.7 nm, +29.1 mV, spherical shape	808 nm; 1.5 W•cm^-2^	GBM orthotopic model (i.v), tumor inhibitory effect	200
	FL/US	PFC@PEPP-Fe, ~180 nm, +35.6 mV, spherical shape	808 nm; 1 W•cm^-2^	4T1 subcutaneous xenograft (i.v), tumor inhibitory effect	201
	PA	GNDs@gelatin, 120.4 ± 26.6 nm, Multibranched spherical shape	808 nm and 1160 nm; 1 W•cm^-2^	C6 subcutaneous xenograft (i.v), tumor inhibitory effect	202
	FL/PA	Organic TTNH, 90.3 nm, -19.89 ± 2.93 mV	808 nm; 1 W•cm^-2^	CT26 orthotopic breast cancer xenograft (i.v.), tumor inhibitory effect	203
NIR-II	CT/MRI/PTI	Au@MnO_2_@PM 246.12 nm, -27.10 mV, core/shell shape	1064 nm; 1 W•cm^-2^	A549 subcutaneous xenograft (i.v), tumor inhibitory effect	234
	FL	SNB, 190 nm, -180 ± 0.3 mV,spherical shape	808 nm; 1.5 W•cm^-2^	4T1 subcutaneous xenograft (i.v), tumor inhibitory effect	235
	PA	CY-1234, 55 nm, spherical shape	1064 nm; 1 W•cm^-2^	4T1 subcutaneous xenograft (i.v), tumor inhibitory effect	236
	MRI/CT	BFS, 9 nm, -17.08 ± 2.13 mV, spherical shape	1064 nm; 1 W•cm^-2^	HT29 subcutaneous xenograft (i.v), tumor inhibitory effect	237

**Table 3:** Radionuclides loaded NPs for radionuclide therapy.

Therapy	Energy (keV)	Half-life	Emission type	NPs-Isotopes (Size, zeta potential and morphology)	Diseases	Ref.
Radionuclide	192	8.02 to 8.04 days	β and γ	^131^I-ICT/R848-MS 48.59 ± 7.2 µm, spherical shape	Orthotopic HCC, (hepatic artery injection) tumor inhibitory effect	276
	5.79 MeV	11.435 days	α	^223^Ra/Ba SAE 310 NM, -13 ± 1.11 mV, nanosheets	Subcutaneous LLC tumor (i.t.) tumor inhibitory effect	278
	192	8.02 to 8.04 days	β and γ	^131^I-VNP+αPD-L1 Nano and micro sized bacterial vectors	Subcutaneous CT-26 tumor (i.t.), tumor inhibitory effect	279
	192	8.02 to 8.04 days	β and γ	USINAS (^131^I-+αPD-L1) 139.31 ± 24.89 nm, spherical shape	Subcutaneous 4T-1 tumor (i.v.), tumor inhibitory effect	280
Cherenkov radiation	897	78.4 h	Blue light	^89^Zr-aEuNP@PEG ~11 nm, square plate	Subcutaneous CT-26 tumor imaging (i.v.), tumor inhibitory effect	293
	511	67.6 to 68 min	Blue light	TiO2 NP-[^68^Ga]Ga-FAPI+TPZ 60 nm, spherical shape	Subcutaneous Panc-1 tumor (i.v.), tumor inhibitory effect	294
	633	109.8 min	Blue light	ECN@TTVP-^18^F-FDG 2067.5 ± 59.3nm, -28.5 ± 0.5 mV, bacteria biohybrid	Subcutaneous CT-26 tumor imaging (i.v.), tumor inhibitory effect	295
	897	78.4 h	Blue light	89ZrALA-liposome-ART 136.5 ± 6.5 nm, spherical shape	Subcutaneous 4T-1 tumor (i.v.), tumor inhibitory effect	296

**Table 4:** Therapeutic NPs used in IMRT and proton therapy.

Therapy	Dose	NPs (Size, zeta potential and morphology)	Diseases	Ref.
Proton	0.5, 1, 2 and 4 Gy	IONP_DOX_ 369.1, 20.9 mV, spherical shape	SW1353 3D spheroids, chemo and radio sensitization effects were observed	310
	2 Gy	Au 25.12 ± 0.79 nm, -18.55 ± 0.15 Mv, spherical shape	MDA-MB 231 3D spheroids, proton therapy effectively reduced cell viability	311
IMRT	50 Gy in 25 fractions	NXTXR3 Hafnium oxide 50 nm, -50mV	Rectal cancer patients, protocol of a phase Ib/II study to investigate the safety profile, dose-limiting toxicity and anti-tumor activity of (NBTXR3) RT+Chemo	315
	50 Gy	NBTXR3 Hafnium oxide 50 nm, -50mV	Locally advanced soft tissue sarcoma patients, NBTXR3 did not negatively affect safety or quality of life and long-term safety	316
	45 Gy in 15 daily fractions	NBTXR3 Hafnium oxide 50 nm, -50mV	Pancreatic ductal adeno carcinoma patients, initial feasibility of local endoscopic delivery of NBTXR3 activated by radiation therapy for patients with pancreatic cancer	317

**Table 5:** NPs mediated cell death pathways and innate immune system activation.

Cell death pathways	NPs (Size, zeta potential and morphology)	Therapy	Therapeutic conditions	Mechanism	*In vivo* cancer therapy		Ref
Ferroptosis	TPEQM-DMA 50 nm, spherical shape	PDT	White light; 100 mW•cm^-2^	GPX4 downregulation	CT26 subcutaneous xenograft (i.t.), tumor inhibitory effect		362
	TPA-NDTA-NP 132 nm, -16.9 mV, spherical shape	PTT	808 nm; 0.3 W•cm^-2^	GPX4 downregulation	Bilateral 4T1 subcutaneous xenograft (i.t. and i.v.), tumor inhibitory effect		364
	DP-HBN/RA 34.26 nm, +21 mV, hexagonal shape	RT	6 Gy	SLC7A11/GPX4 downregulation	4T1 subcutaneous xenograft (i.v.), strong inhibitory effect		365
Cuproptosis	Au@MSN-Cu/PEG/DSF ~250 nm, ~20 mV, core/shell shape	PTT	808 nm; 1 W•cm^-2^	DLAT, LIAS and NPL4 downregulation	4T1 subcutaneous xenograft (i.v.), tumor inhibitory effect		372
	CuSACO 104 ± 9.5 nm, -21.5 ± 29 mV, nanocapsule shape	PTT	1064 nm; 0.4 W•cm^-2^	LIAS downregulation	Orthotopic GL261glioma xenograft (i.v.), tumor inhibitory effect		373
	ES@HM-HfO_2_:Cu 100 ± 5 nm, hollow spherical nanocage	RT	6 Gy (2Gy × 3)	Fe-S cluster proteins downregulation	4T1 subcutaneous xenograft (i.t.), significant tumor inhibitory effect		376
Pyroptosis	ChS-Ce6 74.4 ± 10.2 nm, -22.3 ± 3.6 mV, vesicular shape	PDT	660 nm; 500 mW•cm^-2^	GSDMD	LLC spinal metastasis xenograft (i.v.), strong tumor inhibitory effect		381
	Metal organic framework (IDN@MC) 130 nm, 24 ± 1.27 mV, spherical shape	PTT	808 nm; 1 W•cm^-2^	GSDME	4T1 breast cancer lung metastasis xenograft (i.v.), tumor lung metastasis inhibition		383
	DAC@O-HONS 4.5 nm, spherical shape	RT	6 Gy	GSDME	4T1 orthotopic xenograft (i.t.), strong tumor inhibitory effect		384
STING	BG-Mn ^gel^ hydrogel	PTT	808 nm; 1 W•cm^-2^	pSTING, pTBK1 and pIRF3	B16F10 subcutaneous xenograft (i.t.), tumor inhibitory effect		390
	NP PDT-56 MESS 109.4 nm, -13.9 mV, spherical shape	PDT	808 nm; 1 W•cm^-2^	pSTING, pTBK1 and pIRF3	Melanoma orthotopic xenograft (i.v.), tumor inhibitory effect		391
	Phosphatidyl serine-liposome loaded cGAMP 118.8 nm, -40.7 mV, spherical shape	RT	8 Gy × 3	pTBK1 and pIRF3	4T1 breast cancer lung metastasis xenograft (inhalation), strong tumor inhibitory effect		393

**Table 6:** Comparison of various cell death pathways and their representative nanosystem.

Cell death pathway	Primary inducer	Molecular mechanism	Morphological features	Representative nanosystems
Apoptosis	Caspase activation	Caspase-3/7/9, Cytochrome c, Annexin V+	Cell shrinkage, chromatin condensation, apoptotic bodies	Drug-loaded polymeric NPs, Gold NPs, TiO_2_ NPs
Necrosis	Severe ATP depletion, membrane mechanical or thermal damage	HMGB1 release, random DNA fragmentation	Cell swelling, membrane rupture, organelle breakdown	PTT NPs, Magnetic hyperthermia
Ferroptosis	Iron-dependent lipid peroxidation; GPX4 inhibition	Lipid ROS, GPX4 downregulation, SLC7A11 downregulation	Mitochondrial shrinkage, membrane rupture	Iron oxide NPs, Fe-doped MOFs
Pyroptosis	Inflammasome activation; Gasdermin-mediated pore formation	GSDMD/E cleavage, NLRP3, IL-1β	Cell swelling, large bubbles (pores), membrane rupture	NLRP3-activating silica NPs, LDH-coated NPs
Cuproptosis	Cu-induced lipoylated protein aggregation; TCA cycle stress	Lipoylated DLAT/DLST, FDX1, HSP70	Mitochondrial proteotoxic stress, membrane damage	Copper-sulfide NPs, Cu-doped MOFs, Cu-ionophores

## Abbreviations

PDT: photodynamic therapy
PTT: photothermal therapy
RT: radiotherapy
NPs: nanoparticles
UCNP: upconversion nanoparticle
AuNP: gold nanoparticle
MSN: mesoporous silica nanoparticle
WHO: World Health Organization
PS: photosensitizer
ROS: reactive oxygen species
UV: ultraviolet
NIR: near infrared
FRET: fluorescence resonance energy transfer
Vis: visible
CT: computed tomography
MRI: magnetic resonance imaging
RIT: radionuclide therapy
CR: Cerenkov radiation
FA: folic acid
HA: hyaluronic acid
DOX: doxorubicin
MDR: multidrug resistance
AuNR: gold nanorod
Ce6: chlorin e6
ADR: adriamycin resistance
TP: two-photon
RB: rose bengal
NGO: nanographene oxide
AuNEs: gold nanoechinus
TME: tumor microenvironment
MB: methylene blue
HIF-1α: hypoxia inducible factor-1α
PC: perfluorocarbon
APC: albumin-coated PC nanodroplets
RBC: red blood cells
TPZ: tirapazamine
HAdase: hyaluronidase
SCNP: scintillating nanoparticle
PLNP: persistent luminescent nanoparticles
MOL: metal organic layers
LSPR: localized surface plasmon resonance
PCE: photothermal conversion efficiency
BGV: biodegradable gold nanovesicles
DPP: diketopyrrolopyrrole
SWCNT: single-walled carbon nanotubes
ND: nanodots
HSA: human serum albumin
IPTT: interventional photothermal therapy
TNP: theranostic nanoparticles
N–isopropylacrylamide): PNIPAM, poly
PET: positron emission tomography
LET: linear energy transfer
BBB: blood brain barrier
SPECT: single photon emission computed tomography
ACE: artificial compound eye
CLI: Cherenkov luminescence light
REFI: radiopharmaceutical-excited fluorescence imaging
SCIFI: Cerenkov-induced fluorescence imaging
RET: radionuclide energy transfer
PIXE: particle-induced x-ray emission, IMPT, intensity-modulated proton therapy
IMRT: intensity-modulated radiation therapy
ICG: indocyanine green
NCP: nanoscale coordination polymer
CSiNP: cationic silica nanoparticle
cGAMP: cyclic GMP-AMP cGAMP
STING: stimulator of interferon genes
